# Describing Excited States of Covalently Connected Crystals with Cluster and Embedded Cluster Approaches: Challenges and Solutions

**DOI:** 10.1021/acs.jctc.5c00539

**Published:** 2025-07-24

**Authors:** Michael Ingham, Marcus Brady, Rachel Crespo-Otero

**Affiliations:** Department of Chemistry, 4919 University College London, 20 Gordon Street, London WC1H 0AJ, U. K.

## Abstract

Understanding excited-state processes is essential for designing new functional organic materials. Modeling excited states in organic crystals is challenging due to the need to balance localized and delocalized processes and the competition between intramolecular and intermolecular interactions. Cluster models have proven highly effective for describing weakly interacting organic crystals; however, nonperiodic calculations on periodic systems must account for mechanical and electrostatic coupling to the crystal lattice, particularly in cases of extended coordination where covalent bonds are severed, such as in organic polymers and metal–organic frameworks (MOFs). Point charge embedding is a low-cost method for incorporating long-range electrostatics, enabling the consideration of long-range interactions using Ewald embedding. Small clusters have been effective for modeling excited-state processes in MOFs, yet embedding has rarely been included in such studies. In this work, we examine some of the challenges in describing excited states in covalently connected organic crystals using ONIOM­(QM:QM’) embedding techniques across systems with increasing coordination: diC_4_–BTBT (an organic molecular crystal), polythiophene (an organic polymer), and two MOFs (QMOF-d29cec2 and MOF-5). We analyze the effects of using different electronic structure methods, including TDHF, TDDFT, ADC(2), and CC(2). One of the main challenges is that embedded cluster models are susceptible to overpolarization near the QM:QM’ boundary. To address this, we assess the impact of different charge redistribution schemes (Z-*N* (*N* = 0, 3), RC, and RCD) and implement them in fromage. Additionally, we compare cluster and periodic models. We find that localized models effectively reproduce excited states in both nonconnected systems (diC_4_–BTBT) and fully connected MOFs, whereas polythiophene remains the most challenging due to band conduction. The accuracy of vertical excitations, oscillator strengths, and simulated spectra is strongly influenced by model size, boundary charges, redistribution schemes, and level of theory. We further analyze the effect of vibrational broadening using the nuclear ensemble approach to predict the absorption and emission spectra of MOF-5. Our results provide a heuristic guide for nonperiodic studies of crystalline excited states, highlighting the remarkable relationship between molecular crystals and MOFs, which will be explored in the future work.

## Graphical abstract

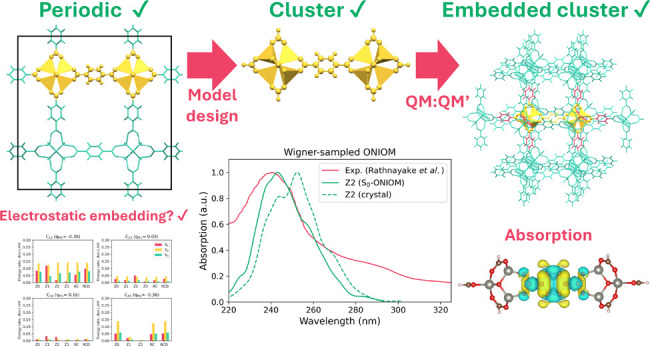

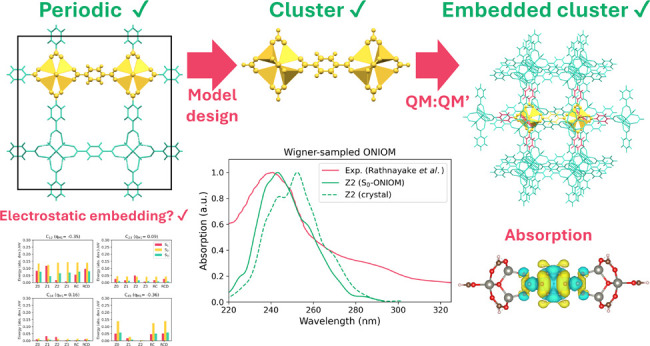

## 1 Introduction

Characterizing the excited-state potential energy surface (PES) of crystalline materials is challenging.^1,2^ The periodic arrangement of molecules in a crystal lattice results in interactions across long spatial distances, not present in the gas phase. Significant delocalization in a molecular crystal makes modeling electronic structure, particularly excited states, difficult. In some cases, the restriction of intramolecular motion by the crystal controls processes such as aggregate-induced emission (AIE) and aggregate-caused quenching (ACQ), even if the relevant excited states are localized.^3,4^ Consequently, individual molecules are both mechanically and electrostatically coupled to the molecular crystal environment.^5^ In extended coordination polymers, such as metal–organic frameworks (MOFs) and organic polymers, the complexity of this problem is compounded by the increased formation of electronic and excitonic bands.^6^ Indeed, many polymer studies require extrapolation protocols in the prediction of vertical excitations and oscillator strengths.^7,8^ However, much of the intriguing photophysics in MOFs, which are composed of modular secondary building units (SBUs), organic linkers and transition metal nodes joined to form a highly porous crystal, arises from direct excitation of the framework, on or between SBUs. As such, MOF excited states are typically characterized as either local excitations centered on the linker (LC) or node (MC), or as charge transfer states between these units (LLCT, LMCT or MLCT).^9,10^ Accordingly, calculations on these building units are often fruitful models for exploring their excited states, similar to molecular crystals.^11–13^

Long-range interactions in crystals are naturally described by quantum chemical methods with inherent periodicity, using periodic boundary conditions. Periodic Kohn–Sham density functional theory (KS-DFT) is the workhorse of the solid-state community; however, in materials that are more molecular in nature, periodic models using plane waves are not necessarily the best approach to describe excited states. In both organic crystals and MOFs, electron and charge transport can be understood through two extreme mechanisms: incoherent hopping and band transport. In molecular crystals with small exciton couplings compared to their reorganization energies, excited states do not significantly delocalize beyond the absorbing molecule or its immediate neighbors, causing incoherent hopping to dominate.^2^ Similarly, many MOFs exhibit minimal band dispersion, also favoring hopping mechanisms.^14^ Additionally, defects within the crystal arrangement disrupt periodic symmetry, influencing the availability of charge transfer states, or other excitations.^15,16^ Consequently, excitations are often better understood using calculations on individual molecules within the crystal in so-called cluster models,^1,17^ rather than on the unit cell itself, because the excited states often localize to within a few molecular units at room temperature.^18^ Cluster models extract a molecule, or a collection of molecules, from the periodic crystal, on which excited-state calculations are performed using molecular codes. In crystals with extended connectivity, such as polymers and MOFs, cluster models must cut covalent bonds, and these dangling bonds must be saturated with hydrogen “link atoms”. Chemical intuition during model construction is required to properly encapsulate the electronic structure of the crystal.

The use of cluster models yields several advantages. First, many high-level methodologies are available, including post-Hartree–Fock (post-HF) and multireference methods, which are imperative when the target wave function is highly correlated and multiconfigurational in nature, for instance, at nonadiabatic regions of the PES.^19^ Second, excited-state methods, although still computationally expensive, come with lower overhead than their plane-wave counterparts. In recent years, efficient periodic implementations have emerged for time-dependent density functional theory (TDDFT);^20^ however, for large crystals such as MOFs (with unit cells exceeding 1000 atoms), even hybrid KS-DFT is challenging. In contrast, TDDFT in molecular codes routinely affords long-range corrected hybrid functionals, which are mandatory for reliable Rydberg and CT states, response properties, and van der Waals interactions.^16,21^ Finally, this favorable cost-to-accuracy trade-off facilitates exploratory methods on the excited-state PES, such as excited-state geometry optimization, conical intersection searching,^22^ and nonadiabatic dynamics.^23^

In turn, cluster models are limited by the omission of long-range interactions. The poor scaling of quantum chemical methods prohibits their application to clusters with 1000s of atoms, the order on which long-range interactions are fully represented. Indeed, TDDFT is generally limited to a few hundred atoms, and only tens of atoms for multiconfigurational methods, depending on the size of the active space. Partitioning a model into regions of different levels of quantum mechanical theory seeks a compromise. In QM:QM’ embedded cluster models, a small region is treated using a high-resolution excited-state (QM) method, and the remaining atoms are treated using a cheaper QM’ method, such as extended tight-binding (xTB),^24,25^ with some coupling energy between levels of theory. This facilitates embedded cluster models with 1000+ atoms and environmental constraints, incurring only a small increase in computational cost.^5^ A myriad of multilayer approaches have been developed in this picture, but the simplest approach to implicitly couple the QM and QM’ calculations is via the ONIOM method (*Our own N-layered Integrated molecular Orbital and molecular Mechanics*).^26–28^ Previously, the ONIOM method has been used extensively to study molecular crystal excited states, in processes such as AIE,^29,30^ clusterization-triggered emission (CTE),^3,4^ excited-state intramolecular proton transfer (ESIPT),^1,31^ and ultralong organic phosphorescence (UOP).^23,32^ However, few studies have explored embedded cluster approaches in the excited states of MOFs,^9^ despite the success of vacuum models in these systems.^11–13^

The success of QM:QM’ in crystal excited states is partly due to the suite of electrostatic embedding (EE) methods used to accurately polarize the QM wave function.^1,2^ Point charge embedding (PCE) is an affordable but accurate alternative to more costly approaches, such as wave function-in-DFT based on frozen density embedding.^33^ In our ONIOM­(QM:QM’)-EE code, fromage, long-range interactions are explicitly evaluated using point charges based on the electrostatic potential (ESP). However, at short-range, the Coulombic potential can become unphysical if there are large point charges situated close to the QM:QM’ boundary. These problematic charges can cause charge density to “leak” into the QM’ region, known as *overpolarization*.^34^ In connected systems, link atoms are necessarily prone to overpolarization due to their proximity to the charge distribution.^35^ The simplest and most widely used method is the Z-*N* scheme (*N* < 3),^36^ which modifies the charge distribution by deleting charges close to the QM:QM’ boundary, providing a practical, low-cost approach to mitigate overpolarization. Various more sophisticated methods have been tried and tested in the ground- ^36–39^ and excited-states;^40,41^ however, Z3 remains the most commonly used in commercial codes. Here, we implement and test the Z-*N* scheme (*N* = 0 to 3), and the RC and RCD schemes from Truhlar and coworkers (see Section 2.2),^36^ in fromage.

In this contribution, we employ cluster, embedded QM:QM’ cluster, and periodic models to study excited states in a range of crystals with varying degrees of connectivity. Using a new implementation in fromage, we perform ONIOM calculations going from molecular crystals to MOFs, taking covalent bond cuts in all cases. In particular, we focus on the role of overpolarization, its relationship with the charge distribution close to the QM:QM’ boundary, and how it can be mitigated using various charge redistribution schemes. We utilize a range of levels of theory and basis sets, and discuss the choice of DFT functional in detail. First, we benchmark our charge redistribution schemes in the nonconnected scenario, a weakly bound organic molecular crystal of symmetric dialkylated benzothieno­[3,2-*b*]­[1]­benzothiophenes (diC_4_–BTBT), making systematic cuts along aliphatic group covalent bonds (Section 3.1). Second, we use polythiophene to illustrate the challenges in cases of strong through-bond conjugation, and the interplay between inter- and intramolecular effects (Section 3.2). Third, we use QMOF-d29cec2, a fully connected but hypothetical MOF, to compare cluster, Ewald-embedded cluster, and periodic excited-state models (Section 3.3) in MOFs. Finally, we collect these findings to compare directly to the experiment and simulate accurate, vibrationally broadened absorption and emission spectra for MOF-5 (Section 3.4), speculating on the excitation mechanism in both cases. Overall, this work seeks to provide a practical overview of the challenges and solutions associated with ONIOM­(QM:QM’)-EE methods in the context of covalently bonded crystals, and provide a perspective on the remarkable similarities between weakly- and strongly bound crystals.

## 2 Background Theory

### 2.1 The ONIOM­(QM:QM’) Method and Link Atoms

Hybrid QM:QM’ methods couple two or more quantum chemical levels of theory to enable combined expressions for the energies, gradients, and Hessians of complex systems.^27^ This is pertinent to excited states, where the chromophoric region often contains far fewer atoms than the molecule of interest but requires a more demanding level of theory (e.g., CASSCF). As the excited-state calculation cannot be performed on the entire cluster (the *’real region’*), the cluster is partitioned into the photoactive region (the *’model region’*) to be modeled at the excited-state level (QM), and the remaining atoms in the system (the environment) at a lower level of theory in the ground state (QM’). The calculations are then coupled either through an explicit interaction energy in the QM:QM’ Hamiltonian or implicitly through a subtractive energy expression.

Here, the ONIOM­(QM:QM’)-EE method is used, where the total energies are obtained using the subtractive equation:

1 $$E_{\mathrm{ONIOM}}^{\mathrm{PCE}}\left(r_{\mathrm{real}}\right)=E_{\mathrm{QM}}^{\mathrm{PCE}}\left(r_{\mathrm{model}}+L\right)+E_{{\mathrm{QM}}^{\prime }}\left(r_{\mathrm{real}}\right)-E_{{\mathrm{QM}}^{\prime }}^{\mathrm{PCE}}\left(r_{\mathrm{model}}+L\right)$$

where **r** denotes the atomic coordinates of the model (**r**_model_) and real regions (**r**_real_), QM denotes the high-level calculation, QM’ the low-level calculation and *L* the inclusion of link atoms in the model region. By subtracting the QM’ calculation of the model region, the double-counted contributions approximately cancel.^1,2,35,42^ PCE refers to the use of electrostatic embedding in QM and QM’ model-region calculations. Accordingly, the individual terms in eq 1 are referred to as *model-high*, *real-low*, and *model-low*. Indeed, the formal objective of the ONIOM equation is to approximate the real-region at the high-level of theory, the *real-high* calculation $\left(E_{\mathrm{ONIOM}}^{\mathrm{PCE}}\left(r_{\mathrm{real}}\right)\approx E_{\mathrm{QM}}\left(r_{\mathrm{real}}\right)\right)$ .

The electrostatic interaction between the QM and QM’ wave functions and the charge distribution of the environment models the region through the Coulomb equation. In conventional QM/MM simulations without electrostatic embedding, the interactions with the environment are at the molecular mechanics level, and the model is therefore said to be mechanically embedded (ME). Indeed, an advantage of QM:QM’ is that the underlying embedding is quantum mechanical, not requiring specific system-dependent parameterizations beyond those already involved in the QM’ method, which is typically a semiempirical approach.

Importantly, the QM:QM’ boundary is the spatial partition between levels of theory (regions); in molecular crystals, the QM:QM’ boundary cuts through space, whereas in connected systems it necessarily cuts through chemical bonds. In the latter scenario, this creates dangling bonds at the QM:QM’ boundary, which must be saturated to avoid creating radical species and a change in multiplicity in the QM calculation, which would introduce a substantial change in electronic structure. Typically, bond cuts are saturated using hydrogen “link atoms”,^26^ although sometimes fluorine atoms, pseudopotentials, or some small group (methyl-, amine-, etc.) may be used. In the present work, hydrogen link atoms are generated using the parameterization from Plett and coworkers (see †ESI).^28^

### 2.2 Charge Redistribution Schemes

Overpolarization may arise from electronic embedding due to the functional form of the Coulomb potential, which becomes infinitely attractive at very short-range, giving rise to the unphysical overlap between the QM densities and the point charges in the QM’ region.^35^ In covalently bonded systems, link atoms are inherently susceptible to overpolarization as the link atom used to saturate the bond lies very close to the partial charge of the link atom host at the QM:QM’ bond. Equally, overpolarization has been observed through nonbonding interactions in the condensed phase. For instance, in our previous embedded CC(2)/aug-cc-pVDZ calculations of the molecular crystal of cytosine, overpolarization via a hydrogen bond (≈ 1.8 Å) resulted in S_1_ energies over 1 eV lower than experiment and periodic references.^17^ Additionally, charge density was observed outside the QM region. When overpolarization arises, modification of the charge distribution is mandatory. The simplest approach is to remove problematic charges near the QM:QM’ boundary and redistribute them.^36,43^ Figure 1 shows how charges are redistributed in the Z-*N* scheme.

**Figure 1:**
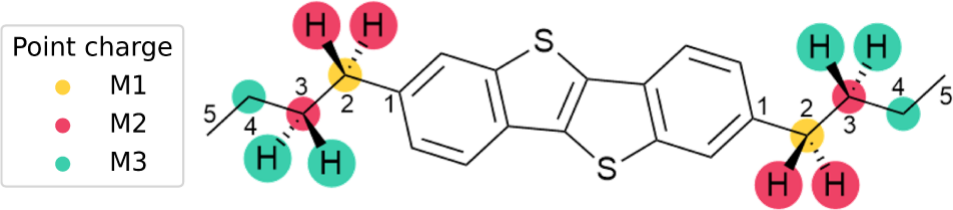
Schematic of the charge redistribution schemes for the diC4-BTBT model, in which the bond cut is made at the C_12_ position. For each Z-*N* scheme, charges up to M-*N* are removed, and the total charge removed is redistributed across the remaining point charges. For the RC and RCD schemes, the M_1_ charge is redistributed to the midpoint of the M_1_-M_2_ bond.

In this example, the QM:QM’ cut is made along each C_1_–C_2_ bond, where C_1_ is the atom in the QM region, termed the link atom connect (LAC), and C_2_ is the atom in the QM’ region that is to be replaced with a hydrogen link atom, termed the link atom host (LAH). Cutting covalent bonds means link atoms must be placed along the C_1_–C_2_ (LAC-LAH) single bond (see †ESI). The potentially problematic point charges in the QM’ may then be defined relative to the QM:QM’ cut, according to the local bonding. The M_1_ charge is on the atom bonded directly to the QM region, M_2_ are the QM’ atoms bonded to the M_1_ atom, and similarly M_3_ to M_2_. Under this definition, all charges up to N bonds are removed. For instance, in the Z2 scheme, M_1_ and M_2_ charges are removed. In all schemes, net charge is conserved by uniformly redistributing the cumulative deleted charge to the rest of the charge distribution.^35^ The Z3 scheme (deleting up to M_3_) is the largest cutoff used. In this work, we implement three commonly used schemes, Z1, Z2, and Z3, to redistribute these charges. In addition to the Z-*N* schemes, we implement the redistributed charge (RC) and redistributed charge and dipole (RCD) schemes of Truhlar and coworkers.^36^ In the RC scheme, each deleted M_1_ charge is redistributed equally to the midpoint of each M_1_-M_2_ bond. In this way, it removes the most problematic M_1_ charge while preserving the M_2_-M_3_ dipole and net charge. The RCD scheme also redistributes charges to the same midpoint but scales the magnitude of the charges to account for the 50% reduction in the M_1_-M_2_ bond length. Consequently, the RCD method preserves the M_1_-M_2_ dipole, rather than M_2_-M_3_, as it lies closer to the model region.

Finally, other sophisticated schemes have been developed, which are not implemented in this work. First, the multilink F* scheme by Truhlar enables cuts with highly polar bonds, and uses tuned pseudopotentials to improve the quality of the charge distribution.^44^ This method provides a useful protocol for MOFs with metal nodes containing hundreds of atoms, and M-L or M-M bonds must necessarily be cut. Here, we take examples of MOFs in which an effective cut can be made through the linker. Second, the ONIOM-CT scheme uses nuclear charges on link atoms to reproduce the electrostatic potential (ESP) of the *real* region.^37^ Finally, the EE­(L1) and EE­(L2) methods from Caricato et al. use constrained Restrained Electrostatic Potential (RESP) fitting to self-consistently tune the charge distribution with respect to the ESP of the entire cluster.^41^ The RESP fit for the MOF real-low calculation was prohibitively expensive for large clusters, despite the availability of improved ESP fitting implementations.^45^

### 2.3 Population Analyses and Charge Assignment

The quality of the population analysis used to generate the PCE is important for the accuracy of the embedded cluster calculation. The method used to generate the charge distribution differs between the molecular crystal and the fully connected systems. In the molecular crystal ONIOM calculations, PCE is generated using the standard methods implemented in fromage.^2^ First, a population analysis is performed on an S_0_ or S_1_ (TD-)­DFT calculation on the QM model region to obtain the atomic charges. Of course, charge is not a quantum mechanical observable and therefore introduces a degree of subjectivity in how it is calculated. Generally, methods that calculate the ESP provide a favorable trade-off between accuracy and cost, removing the dependence on basis set present in more basic schemes, such as Mulliken charges.^1^ As such, we use the RESP and the Repeating Electrostatic Potential Extracted ATomic (REPEAT) charges for the molecular crystal calculations. The charges on the QM model are then rapidly redistributed across the shell region, where the connectivity of the molecule is used to average charges on atoms in chemically equivalent environments.

Additionally, the modified Ewald method in fromage can be used for the exact treatment of long-range interactions, where summation can be used to approximate the true Madelung potential of the crystal.^1,2,46^ In the summation, the full electrostatic potential is divided into two rapidly converging short- and long-range series,

2 $$V_{\mathrm{Ewald}}\left(r\right)=\underset{Ls}{\sum }q_{s}\frac{\mathrm{erfc}\left(\gamma |r-L-R_{s}|\right)}{\left|r-L-R_{s}\right|}+\frac{4\pi }{v_{c}}\underset{G\neq 0}{\sum }\frac{1}{G^{2}}e^{-G^{2}/4\gamma^{2}}\left[\underset{s}{\sum }q_{s}e^{iG(r-R_{s})}\right]$$

where **R**_s_ is the unit cell lattice site, *q*_s_ is the charge at each site, γ is the Ewald constant, *v*_c_ is the unit cell volume, and **L** and **G** are the real (**L**) and reciprocal (**G**) lattice translations.^1^ From this, an array of around 10,000 point charges can be generated to approximate the exact long-range potential without creating artificial dipoles, as implemented by Derenzo and co-workers.^46,47^ In our previous studies, the defect is selected as a specific configuration of QM molecules (e.g., a dimer) or isomers.^15,30,48^ Ciofini et al. have used self-consistent Ewald embedding in photophysical studies on organic crystals^49–51^ and organic–inorganic perovskites,^52^ where the Ewald embedding was used to generate the condensed phase (i.e., periodic) electrostatics in an otherwise nonperiodic calculation. Recently, we have evaluated the performance of ground- and excited-state charges, self-consistent charges, and Ewald-embedded cluster models on a subset of the X23 molecular crystal database,^32^ where overpolarization of some Ewald-embedded calculations was observed. The Ewald calculation still requires an underlying population analysis, which is assigned to the (neutral) unit cell as before. Ewald embedding can be used on both molecular crystals and crystals with extended coordination. Ewald was tested only for QMOF-d29cec2 here, as we have established its efficacy in organic molecular crystals previously;^1,2,17,32^ however, the methodology can be rapidly deployed to other MOFs.

Finally, the charge assignment approach for molecular crystals does not straightforwardly extend to covalent systems due to the introduction of link atoms. Any isolated population analysis cannot be extrapolated to the full cluster because the link atoms are not present, meaning some charge will be lost in the process. This gives no guarantee of neutrality (or integer charge) and would require ad-hoc correction. Instead, we can use the Mulliken population analysis provided by the real-low xTB calculation. Mulliken charges are not optimal due to the small basis set used in xTB, which describes atoms with a minimal basis and adds polarization basis functions for heavier atoms (*Z* > 9). However, they are included automatically with each xTB calculation and can therefore be readily applied to geometry optimizations of a large embedded cluster model.

## 3 Results

### 3.1 diC_4_–BTBT

First, we demonstrate the effect of overpolarization on the vertical excitations and oscillator strengths in crystalline diC_4_–BTBT. This organic semiconductor crystal, a symmetric dialkylated benzothieno­[3,2-*b*]­[1]­benzothiophene, has been investigated due to its applications in high-performance organic thin-film transistors.^53^ The chemical structure is shown in Figure 1 and the model is shown in Figure 2.

**Figure 2:**
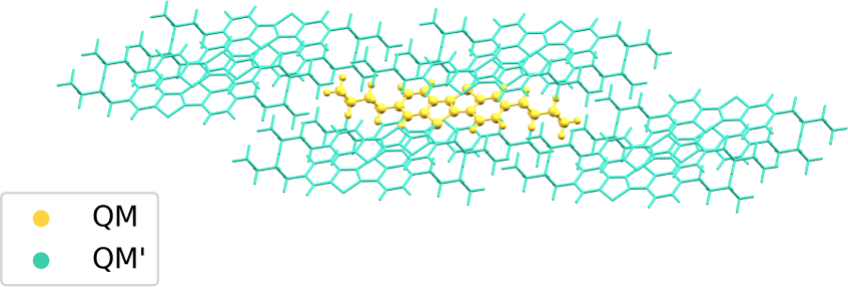
The diC_4_–BTBT cluster model used in terms of its *model* (yellow) and *env* (green) regions. In this case, the QM:QM’ boundary does not cut through any bonds. In our C_*nm*_ models, we cut the alkyl chain at the corresponding *n* and *m* carbons.

Cutting along the aliphatic group in diC_4_–BTBT is favorable because the single C–C bonds are nonpolar and are not part of the chromophore.^35^ The minor inductive effects of the chain only minimally perturb the corresponding excited states of the π-electrons, thereby making this a useful test system. Figure 3 shows the excited states and oscillator strengths of each model at the CC(2)/def2-SVP level of theory. The absolute deviation from the full ONIOM calculation shows that model size and the M_1_ charge value control the accuracy of both the excited-state energy and oscillator strength in the truncated model. In C_12_, the smallest model with the link atom directly bonded to the chromophore, S_2_ has the largest absolute error, showing deviations of 0.15 eV across each charge distribution. Smaller errors were observed for S_3_ (under 0.09 eV). Interestingly, S_1_ shows a large dependence on charge redistribution, where the deviation is high under Z1 (0.124 eV) but very low for Z2 (0.017 eV) and Z3 (0.008 eV). The poor performance of this model is due to both the bad position of the bond cut, near the chromophore, and the large size of the M_1_ charge at −0.35 e, resulting in overpolarization. In contrast, the largest model, C_45_, is very close in size to the full ONIOM reference but has a similar M_1_ charge, isolating the effect of the charge distribution. This shows that the M_1_ charge dominates the polarization of the QM region and is therefore the most important to remove.^34^ As such, we see the dependence on charge redistribution, arising due to overpolarization, present for all three states, with Z2 providing the best performance, with errors of 0.003 to 0.004 eV, very close to that of the reference. Similarly, the second-largest model, C_34_, has the opposite effect: the model is sufficiently large but has a smaller M_1_ charge at −0.35 e at 0.16, resulting in a much smaller overpolarization effect. Consequently, the deviations are generally below 0.01 eV, regardless of the charge scheme. Finally, in C_23_, the bond cut is within two single bonds from the chromophore,^35^ and consequently, model size starts to play a role with errors approaching 0.05 eV.

**Figure 3:**
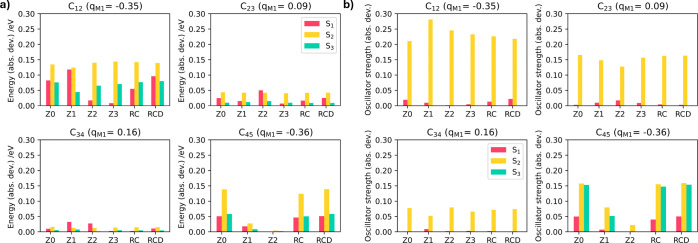
a) Absolute deviation in S_1_–S_3_ energies (CC(2)/def2-SVP) for each truncated model compared to the full molecule embedding in RESP charges at the periodic DFT geometry and redistributed via each charge scheme. b) Absolute deviation in S_1_–S_3_ oscillator strengths (CC(2)/def2-SVP) for each truncated model compared to the full molecule embedded in RESP charges at the periodic DFT geometry and redistributed via each charge scheme.

Similar patterns emerge in the calculated oscillator strengths, but the case is more complex due to the sensitivity of the transition dipole moment, which determines the brightness of the state, to polarization. In the embedded CC(2) reference (Table S1, see †ESI), S_1_ has low strength (*f* = 0.1), S_2_ is bright (*f* = 0.7), and S_3_ is dark (*f* = 0.0). Similar to the excited-state energies, in C_12_, C_23_, and C_34_, the relative errors are most significant in S_2_ due to a poor prediction of the oscillator caused by the size of the model, with only a small dependence on the charge distribution. This seems to suggest that, in this case, the oscillator strength is most dependent on the length of the chain, and indeed variation is also seen in S_1_. However, due to the low strength of the state, the absolute deviation is much smaller. In all three models, S_3_ is consistently predicted to be dark, resulting in no error. Interestingly, this scenario changes entirely for C_45_ because, although there are enough carbons to correctly model the state, a similar pattern to the excited-state energies emerges, with a much stronger dependence on the local charge distribution. This is significant, as in this case, overpolarizing charges cause S_3_ to be bright (*f* ≈ 0.15) under the Z0, Z1, RC, and RCD schemes, a qualitative change in the character of the state. For our CC(2)/def2-SVP calculations, only C_45_ under the Z2 scheme correctly predicts the first bright state, and consequently, the error is almost entirely removed.

The RC and RCD schemes perform less well than anticipated, generally displaying overpolarization similar to that of the Z0 scheme, indicating that overpolarizing charges have not necessarily been redistributed. The poor performance is likely due to the charges not being moved sufficiently far from the model region. In the past, these methods have been effective in ground-state electronic structure; however, further tuning of the position of the *q*_0_ charge is likely required, depending on the diffusivity of the excited states. The *q*_0_ charges were redistributed to the midpoint of the M_1_-M_2_ bond to simplify the dipole correction in the RCD scheme. Moreover, the simplicity of the Z-schemes is advantageous. The relationship between diffusivity and overpolarization is important in the modeling of Rydberg states, where one would anticipate optimal performance for Z3.

Similarly, overpolarization depends on the choice of quantum chemical method (Figures S2 and S3, see †ESI). The deviation of S_1_ energies calculated from TD-HF, TD-PBE, and TD-PBE0 in the TZVP basis set highlights this relationship. Indeed, these two properties are related. For instance, TD-HF/TZVP shows almost no dependence on charge redistribution or the size of the M_1_ charge in models C_12_ to C_34_, indicating minimal overpolarization, unlike PBE and PBE0, suggesting that model size is the most important factor. In C_45_, the Z1 and Z2 schemes correct before. This implies that the error in TD-HF is primarily due to the model size, and electrostatic embedding is less effective as a correction. In contrast, TD-PBE is the most overpolarized. For instance, in C_12_, there are deviations of over 0.04 eV for the Z0, Z1, RC, and RCD schemes, with minimal deviation only for the Z2 and Z3 schemes. Similarly, very large deviations of over 0.2 eV are seen for the Z0, RC, and RCD schemes in C_45_. Surprisingly, however, TD-PBE0/TZVP shows a similar but slightly smaller error to TD-PBE, except in C_45_, where the errors are as much as 0.1 eV smaller. This difference is explained by the inclusion of exact HF exchange in the functional. Proper accounting of nonlocal effects through exchange in PBE0 results in a greater degree of localization in the excited state, reducing susceptibility to overpolarization compared to the GGA calculations.

In turn, the small deviations in TD-HF are unsurprising, as 100% of the exchange integral is included, resulting in highly localized states and minimal dependence on the charge distribution. Generally, we do not observe a strong dependence on basis set (Figure S2, †ESI), at least for TD-PBE0. We must emphasize that the smaller error is relative to the respective full ONIOM calculation, and not indicative of the quality of the underlying quantum chemical theory. For instance, the energies of the S_1_ state vary widely: 4.50, 3.47, 4.00, and 4.32 eV for TD-HF, TD-PBE, TD-PBE0, and CC(2)/def2-SVP, respectively. Moreover, diC_4_–BTBT is an organic semiconductor and is therefore subject to additional challenges, as we will see in polythiophene (Section 3.2).

Overall, the results for diC_4_–BTBT suggest that redistribution under the Z-scheme is judicious and our implementation is valid, at least in the case of cutting carbon single bonds. These findings hold true for a range of basis sets and methods. Consequently, these approaches will be used in our ONIOM­(QM:QM’) calculations going forward. The neutrality of the bond, as indicated by the size of the M_1_ charge, also plays a critical role. These findings support those established in the QM/MM literature, which particularly focus on a ground-state model region.^35,36^ Here, we show that the Z-scheme extends to excited states. Throughout the diC_4_–BTBT results, the perturbation is generally small (<0.2 eV), reflecting that these QM:QM’ studies are less susceptible to overpolarization by PCE. This supports our previous work on the molecular crystal of cytosine, where overpolarization could be corrected using the Z_int_ scheme.^17^

In the past, similar problems have been solved for ADC(2) within the FDET approach, where the approximate kinetic-energy functional does not properly account for Pauli repulsion.^54^ In that case, all-electron pseudopotentials were necessary to correct the charge spill-out in a set of chromophores embedded in different chemical environments. Moreover, this suggests that these findings extend beyond single excitations. This is important because methods containing doubly excited determinants, like CC(2), are essential in systems such as polyenes, where a double excitation from the HOMO to LUMO can significantly influence the excited-state properties.^55^

### 3.2 Polythiophene

This section demonstrates the challenges of truncating a conjugated π-system, polythiophene, and the limitations of using electrostatic embedding *in lieu* of a sufficiently large model. The models of the QM regions are shown in Figure 4; more details about the models can be found in the SI. The number of thiophene units in the model has a profound influence on the excited state than the nature of the PCE, and in particular, the through-bond conjugation controls the vertical excitations and oscillator strengths (Figure S4, see †ESI for detailed discussion).

**Figure 4:**
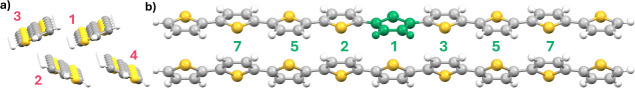
Visualization of the polythiophene aggregate real-high model (C: gray, H: white, S: yellow) containing four chains of nine thiophene units (4–9) from the top (a) and side (b) perspectives. An example thiophene unit is shown in green. The indices in red show the order in which new chains are added to the model, and the indices in green show the order in which thiophene units are added, to ensure a balanced charge distribution in each cluster model. A terminating hydrogen link atom is added to each embedded cluster.

The aggregate model for polythiophene enables investigation of the balance between intramolecular and intermolecular interactions. The real-high reference (4 chains of 9 thiophene units, see Figure 4) has a dark S_1_ state at 2.22 eV with ωB97X-D/cc-pVDZ, approaching the experimental literature value of ≈ 2 eV.^56^ The first 5 singlet excited states, S_1_ to S_5_, are predicted to lie within a narrow energy range (2.22 to 2.92 eV), where S_1_ (2.22 eV) is very weakly allowed (f = 0.003) and S_4_ (2.56 eV) is a very intense bright state, with exceptionally large oscillator strength (*f* ≈ 12). This is a feature of extended conjugated polymers, arising from the Thomas-Reiche-Kuhn (TRK) sum rule and the dense first absorption band. For instance, in a similar polymer, PPV, very large oscillator strength of the 1^1^B_*u*_ was observed with RI-ADC(2) on long-chain cluster models, where S_3_ (1^1^B_*u*_) is very bright in the infinite chain extrapolation.^8^ These results suggest that TD-ωB97X-D/cc-pVDZ provides a balanced description of the electronic structure, compared to crystalline polythiophene, and therefore the (4–9) model serves as a reasonable “exact” reference model.

The performance of the embedded cluster models is measured in two ways. First, for energies, we compare the absolute error between the S_1_ energy of each model to the (4–9) reference. Second, for oscillator strengths, we compare the absolute error in oscillator strength between the bright state of each model to the bright state (S_4_) of the (4–9) reference. This is because the bright state changes with the number of polythiophene chains in the model. The resulting error matrices (Figure 5) gauge how the intramolecular effects improve along the *x*-axis and intermolecular effects along the *y*-axis.

**Figure 5:**
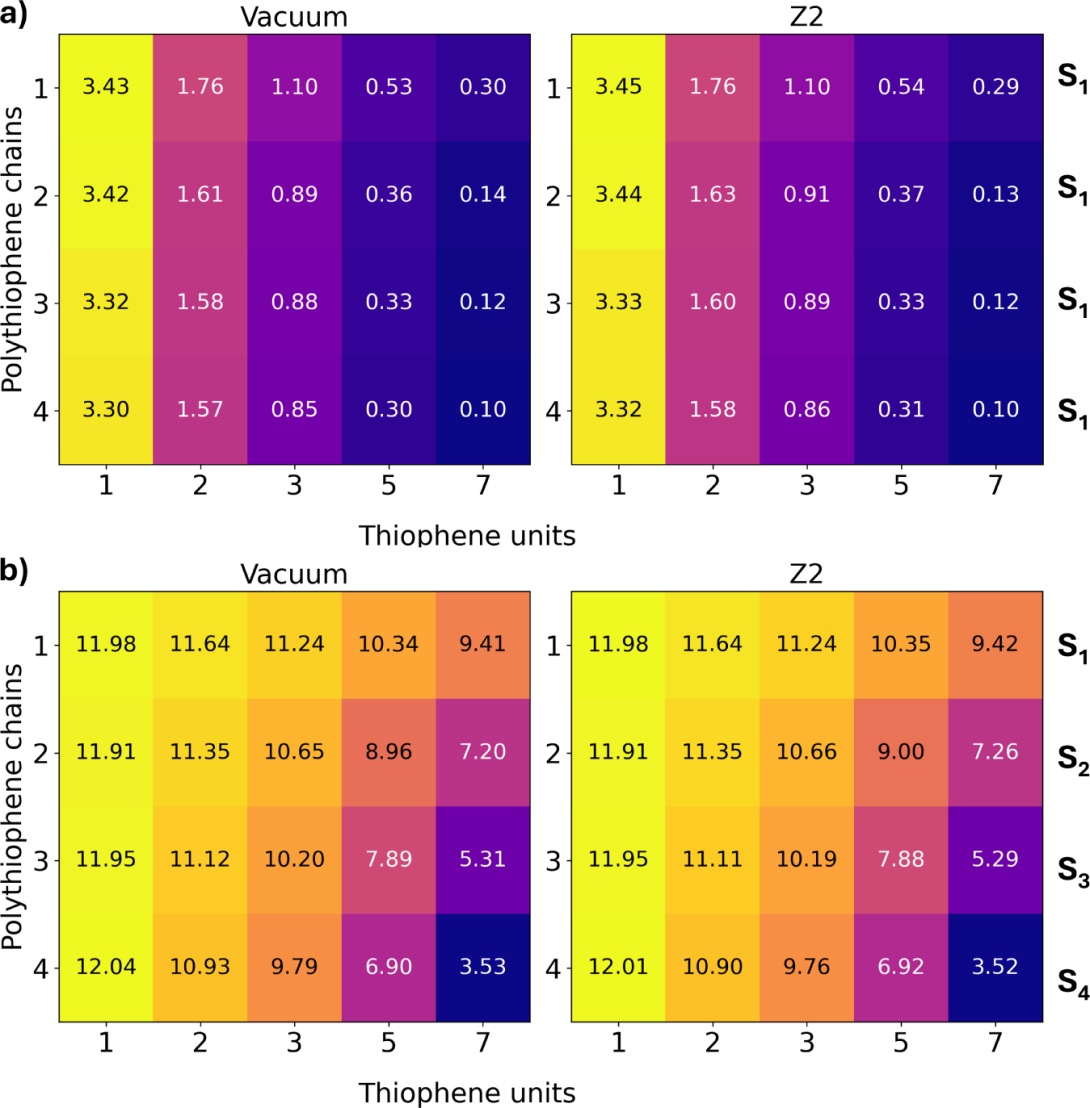
Absolute error between the (4–9) reference model and each of the aggregate models: a) S_1_ energy compared to the (4–9) S_1_ reference, b) oscillator strength of the model bright state compared to the (4–9) S_4_ reference. The state of the model is indicated next to each matrix. All calculations were performed using TD-ωB97X-D/cc-pVDZ.

Clearly, from the figure, a critical dependence is observed on both the number of chains and the number of units in each chain, with and without point charge embedding. Figure 5 shows that increasing the number of chains has a smaller effect on the S_1_ energy than increasing the number of units in the chain. For instance, the 1-unit chains show errors in the range 3.30–3.45 eV, significantly overestimated with respect to the reference. Going from 1 to 4 chains results in a stabilization of 0.13 eV in the vacuum case, indicating the stabilization from aggregation (0.13 eV) is far smaller than the error from too small a chain (3.3–3.4 eV). As such, extending the chain from 1 to 7 thiophene units results in errors of 0.10–0.30 eV, very close to the (4–9) reference. At this resolution, the aggregation effect is substantial, as the (4–7) over 0.2 eV more stable than the (1–7) model (twice the error 0.1 eV absolute error for the (4–7) model). In other words, when the through-bond effect is sufficiently accounted for, the intermolecular effect must now be carefully considered.

The case for oscillator strengths is interesting (Figure 5b), because both intra- and intermolecular effects strongly affect the oscillator strength. For instance, the 1-unit chain shows deviations from 11.98 to 9.42, meaning even though the S_1_ energy is well-described, the oscillator strength is massively underestimated. For the seven-unit models, increasing the number of chains drastically improves this from deviations of 9.42 to 3.52. The (4–7) model provides the closest oscillator strength, but is still underestimated by 3.52, showing it is a challenging property to predict from the cluster model. For both energies and oscillator strengths, the model itself is most important, and cannot be corrected by point charge embedding alone.

As such, polythiophene serves as a highly delocalized example, in which aggregation leads to the formation of a dense excitonic band structure. In essence, this is the limit where cluster models break down due to band conduction. However, in cases where the electronic structure is localized and the excitonic coupling is low, these bands become far more separated, enabling a complete description of the excited states from modestly sized cluster models. Clearly, careful model benchmarking is required depending on the desired property. Remarkably, in previous nonadiabatic simulations on polythiophene, just four oligomer units were sufficient to recover accurate time constants for the periodic crystal.^57^ This raises the question: in the absence of band conduction, how small can a model be to characterize the key bands of a crystal? This will be explored further in QMOF-d29cec2 in the next section.

### 3.3 QMOF-d29cec2

Having now investigated weakly bound molecular crystals (diC_4_–BTBT) and crystals with extended conductivity in one direction (polythiophene), we can now explore MOFs as fully connected systems. As an example, QMOF-d29cec2 has a composition similar to other useful MOFs, such as MOF-5, but with a more manageable unit cell.^58,59^ The structure was predicted from a high-throughput study, so we use a periodic reference at a comparable level (TDA-ωB97X-D) to our embedded cluster calculations. Ground-state Ewald charges were used because we are investigating vertical excitations of the framework, using a geometry that has been shown to reproduce the fundamental band gap accurately compared to periodic data.^9^ Figure 6 shows the Ewald embedded cluster model. The charge distribution near the QM:QM’ boundary, as demonstrated for diC_4_–BTBT, determines the extent of overpolarization. The periodic population analyses (see Table S8 and †ESI for detailed analysis), used to generate Ewald embedding, showed that M_1_ carbons near the linker are essentially neutral, making the C–C bond a suitable QM:QM’ cut, although the larger node M_1_ charges will be seen to be more challenging. Nevertheless, this is preferable to the M-L bond, which would require careful tuning due to the high polarity of the bond.^44^

**Figure 6:**
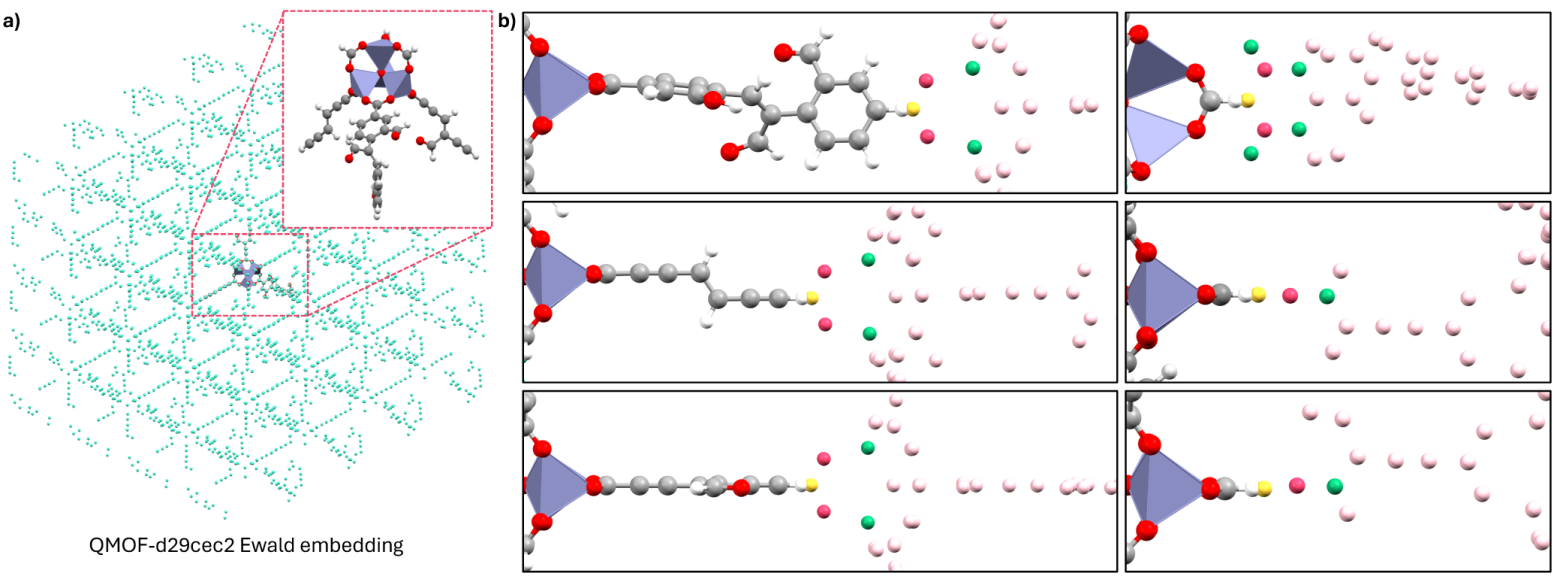
a) Visualization of the QMOF-d29cec2 Ewald embedded cluster model (Zn: purple, O: red, C: gray, H: white). b) The local charge distribution at each QM:QM’ cut possible. The M_1_ (yellow), M_2_ (pink), and M_3_ (green) are highlighted while the remaining Ewald charges are shown in light pink.

First, we compare periodic and cluster models in vacuum within a common periodic TDA-DFT framework using the GPW method,^20,60^ at a common geometry, providing a very close comparison between models. The coordinates in each model are the same as those in the relaxed unit cell, with hydrogen link atoms introduced to the cluster model. Figure 7 shows TDA-ωB97X-D vertical excitation energies (up to S_3_) alongside the dominant natural transition orbitals (NTOs) for each state. For the 1 × 1 × 1 (111, hereafter) model, the energies of 3.08, 3.19, and 3.55 eV, respectively, are in close agreement with the isolated cluster model in vacuum, particularly for S_2_ (3.19 eV) and S_3_ (3.57 eV). Notably, the S_1_ energy (2.95 eV) is slightly underestimated in vacuum. Given the excellent agreement of the other states, this is likely an artifact of a nonoptimal capping atom at the QM:QM’ boundary rather than the level of theory. Nevertheless, this discrepancy is small (0.13 eV). The generally good agreement between the vacuum and cluster models is perhaps explained by the breaking of π-conjugation on the linker at the organic–inorganic interface. Essentially, the hydrogen atom lies in the nodal plane of each ππ* and does not contribute explicitly to each excited state. For S_1_, in the terminal alkyne group, the excitation occurs between the pair of π-orbitals. Furthermore, the NTO analysis shows that the electron and hole orbitals are centered on the same linker for each excitation, indicating that the excitation is ligand-centered (LC) in character. Moreover, the well-separated energy levels suggest that excitonic coupling is not substantial in QMOF-d29cec2, reflecting that the cluster model is an excellent approximation of the solid state.

**Figure 7:**
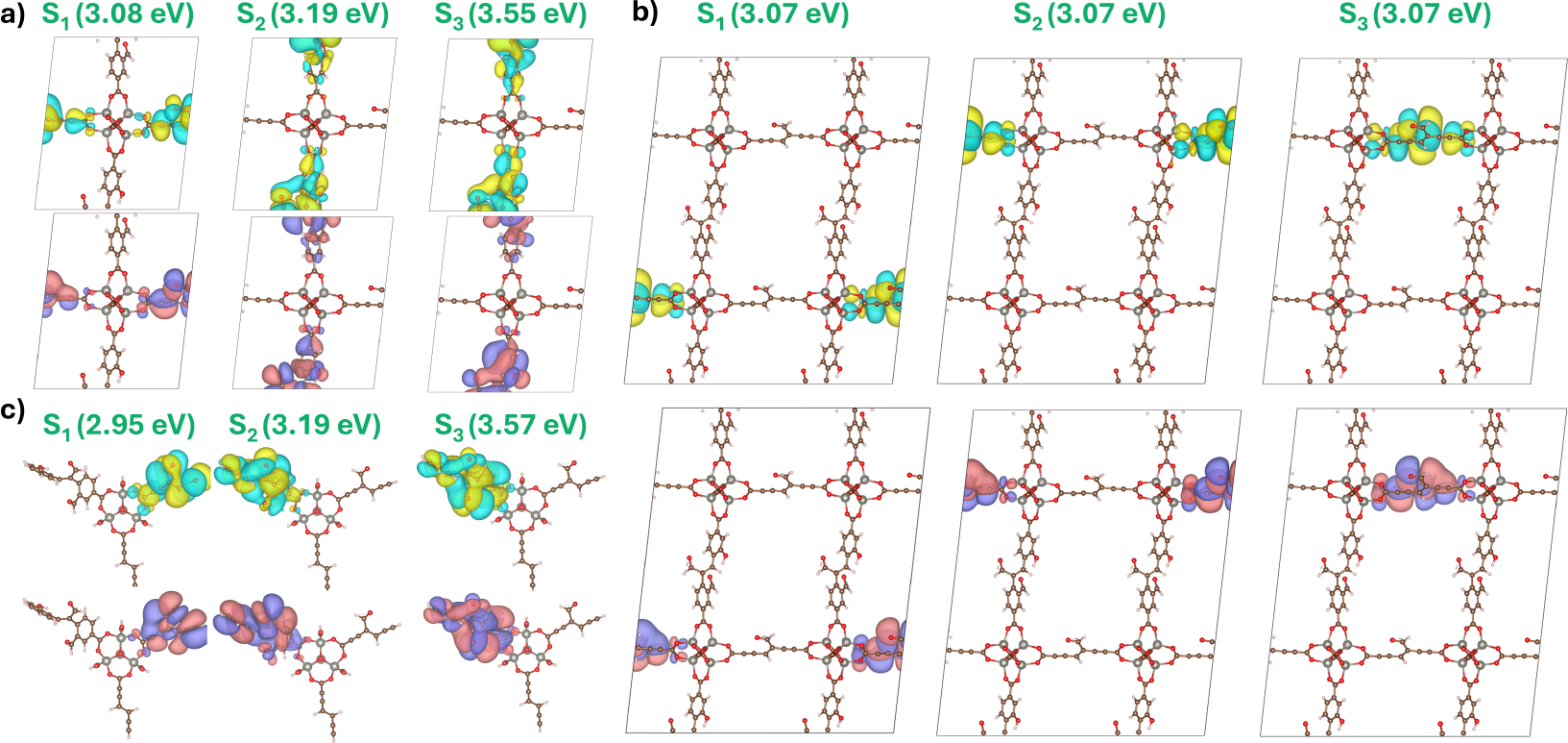
Comparison of natural transition orbital (NTO) analysis from a periodic TDA-ωB97X-D calculation on a) the conventional unit cell, b) the 2 × 2 × 2 supercell (right), and c) a cluster model in a 27.1 × 27.1 × 27.1 Å^3^ box. The dominant hole (yellow/green) and electron (purple/pink) orbitals are shown for the first three excited-state transitions. The TZVP-MOLOPT/admm-dzp basis set with the GTH-PBE pseudopotentials was used for each TDA-ωB97X-D calculations.

Increasing the supercell dimensions is a common convergence check for Γ-point-only calculations,^61^ and in this case, it is preferred over denser *k*-grids due to the better scaling of the method. Figure 7 shows that the first three TDDFT singlet states on the 2 × 2 × 2 (222) supercell are degenerate (3.07 eV), and indeed differ in energy from the S_1_ energy of the 111 calculation by less than 0.01 eV. Interestingly, visualization of the NTOs shows that the orbitals are very similar to those of the 111 S_1_ state, but localized on different, but equivalent, linkers. Indeed, the interpretation is that there are eight near-degenerate vertical excitations with essentially no excitonic coupling between states. This has a profound impact on how model construction is perceived. To gain an understanding of the key excitation bands, a significantly larger number of states must be calculated to capture the characteristics of each band. In this case, to understand up to S_3_ in 222, excitations up to S_24_ would be required-three excitations for each of the eight linkers. In practical terms, this is a significantly larger calculation on a much larger number of atoms, leading to a large increase in cost, which, in reality, offers no further insight into the excited states. This is important for modeling absorption spectra, particularly if one is interested in excitations beyond the first absorption band. Of course, caution is required when using a truncated cell, but for QMOF-d29cec2, the 111 model offers a good reference point. In polythiophene, this truncation may be unacceptable. Moreover, the localized electronic structure of MOFs, and the ability of both models to capture the character of the low-lying excited states, make it unsurprising that small cluster models can perform so well against experimental MOF studies.^11,13^

Finally, we introduce Ewald embedding in the cluster model, performing the TDA calculations at the same common geometry as the vacuum calculation. Figure 8 shows the excited states (S_1_ to S_3_) of the embedded TDA-ωB97X-D cluster calculations. The vacuum calculation in the GPW method and molecular code are very close in energy, indicating that ωB97X-D/TZVP is reasonable compared to the fully periodic reference. Figure 8 shows the vertical excitations with embedding, which has the strongest influence on the Z1 scheme, reducing the energies of the excited states, with the largest deviations (0.1–0.5 eV) for S_2_ and S_3_ with respect to the reference. Similarly, Z3 is also affected by the embedding, influencing only S_2_ and S_3_. In the RESP/Ewald embedding, the M_1_ charges in the Zn node (Table S8, †ESI) are large (0.44 to 0.60 e) and overpolarizing, while the M_1_ charges in the linker are nearly neutral. In principle, this should make these excited states, localized on the linkers, more susceptible to polarization by the larger M_1_ charge, as was shown for diC_4_–BTBT. However, Z0 shows improved agreement with the reference. The case is similar for RC and RCD. This is once again explained by the position of the link atom within the node of the ππ* states. Visualization of the density differences (Figure S5, †ESI) shows that in the best performing models (Z0, Z2, RC, and RCD), there is no density localized on the linker, but a region of electron density is present in Z1 and Z3. Because of this, one would cautiously select Z2 going forward to avoid issues when the nuclei are allowed to move, for instance in geometry optimization or dynamical studies. Similar results are observed for the REPEAT and Mulliken calculations (Figure S6, †ESI), where the Mulliken scheme shows the smallest effect because the sizes of the charges in the distribution are much lower. This highlights the importance of carefully selecting the charge distribution, where ESP-based charges are most chemically justified.

**Figure 8:**
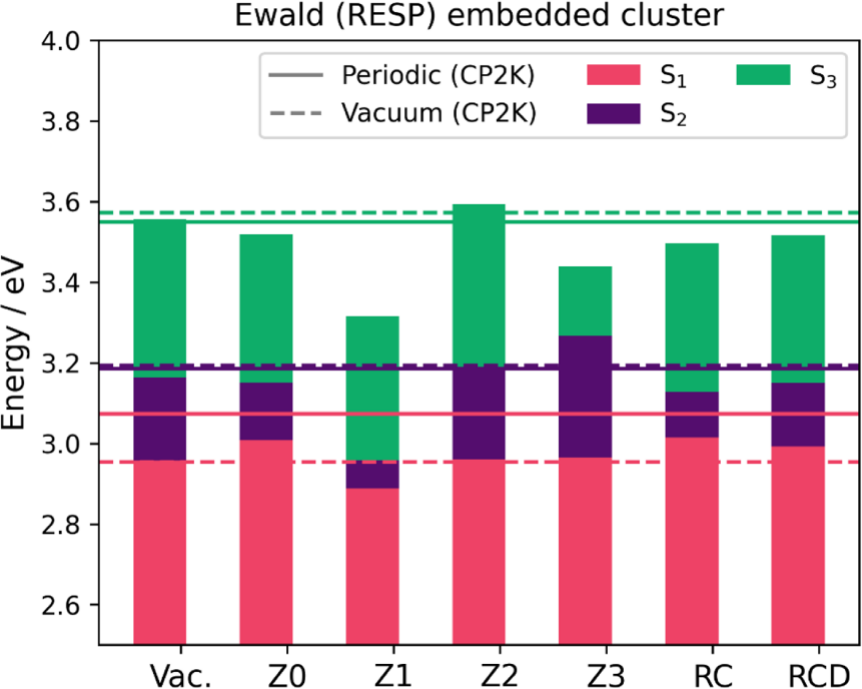
Energy of S_1_–S_3_ from TDA-ωB97X-D/TZVP RESP-Ewald-embedded cluster for QMOF-d29cec2, and under each redistribution method. The isolated cluster calculation is also shown. The solid lines refer to the periodic TDA-ωB97X-D of the isolated cluster (dashed) and the 1 × 1 × 1 cell (solid).

Overall, this highlights a key difference between molecular crystals and MOFs. While molecular crystals often have close packing, and are strongly influenced by electrostatics, the large pores of MOFs clearly reduce this effect. Especially in the case of QMOF-d29cec2, point charges are situated far from the QM wave function. Given the $\frac{1}{r}$ scaling of the Coulomb potential, the evaluation is low and the model is not polarized by the environment, resulting in excellent performance of the vacuum model. Indeed, only those lying close to the QM:QM’ boundary ought to have a large effect. After all, the error cancellation inherent to quantum chemical simulations, particularly density functional methods, merits a larger degree of benchmarking on the choice of redistribution.

### 3.4 MOF-5

#### 3.4.1 Absorption

In our final section, we investigate absorption and emission in MOF-5, going beyond theoretical benchmarks to directly compare the results with experimental data. This demonstrates the utility of cluster and embedded cluster models in reproducing the photophysical properties of MOFs. First, we examine three isolated cluster models of increasing size: H_2_BDC, the organic linker, Fragment A, and Fragment B (Figure 9). These MOF clusters have previously been used to investigate exciton binding in MOF-5.^62^ Focusing first on absorption, our results suggest that model size, DFT functional, and vibrational effects play key roles in the accuracy of the simulated spectrum.

**Figure 9:**
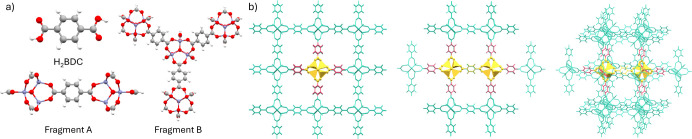
a) Visualizations of the three models (Zn: purple, C: gray, O: red, H: white) used for MOF-5: the H_2_BDC linker, Fragment A (1 linker and 2 nodes), and Fragment B (3 linkers and 4 nodes). b) Visualizations of the embedded cluster model used for the ONIOM calculations of MOF-5, generated with fromage. Atoms in the QM region are yellow, and the QM’ are pink and green. The pink region indicates the atoms that are allowed to move during the optimization. All models are capped with hydrogen link atoms where bond cutting was necessary, except for the H_2_BDC model where the model is capped with a carbonyl group.

Figure 10 shows the absorption spectra obtained in vacuum for H_2_BDC, Fragment A, and Fragment B, as well as for the embedded models ONIOM­(CAM-B3LYP:xTB)-EE obtained for Fragment A, considering both the periodic PBE-D3 geometry and the geometry optimized at ONIOM­(CAM-B3LYP:xTB)-EE. This model is embedded in a large cluster and electrostatically embedded using the Z-*N* schemes outlined earlier. We consider simple Gaussian and vibrational broadening, generated by the nuclear ensemble approach (NEA) using stochastic sampling of a Wigner distribution based on the frequencies obtained for the Franck–Condon (FC) geometry, as implemented in Newton-X.^63,64^ The experimental absorption spectrum is shown between 220 and 320 nm to capture the first absorption band.^65^ The spectrum shows an intense absorption maximum at 241 nm, with a long tail extending beyond 300 nm, a challenging feature to capture without considering the coupling of atomic vibrational motion to the excited states.

**Figure 10:**
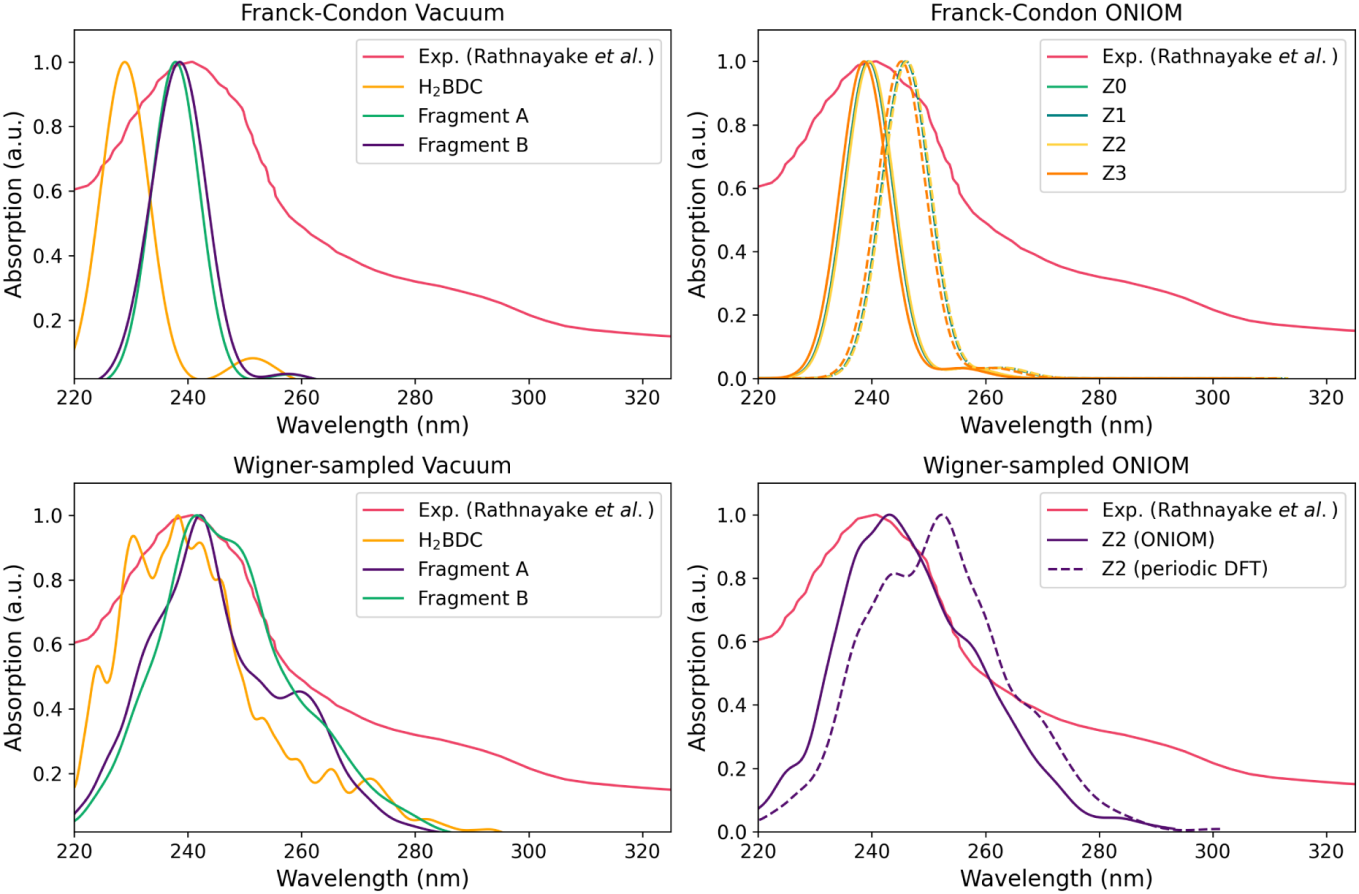
Absorption spectrum of MOF-5 (TD-CAM-B3LYP/cc-pVDZ) compared to the experimental absorption spectrum (ref 65 In each subplot, Franck–Condon refers to spectra computed by phenomenological broadening of the Franck–Condon (FC) vertical excitations (S_1_–S_15_) with Gaussian curves (fwhm = 10 nm). Wigner-sampled plots were obtained considering the NEA (200 configurations). Vacuum refers to the cluster models relaxed in the gas-phase. ONIOM refers to the embedded cluster models, both at the periodic DFT geometry (dashed) and the relaxed ONIOM geometry (solid). Z0, Z1, Z2, and Z3 refers to the charge redistribution scheme.

For the Gaussian-broadened vertical excitations, the intense bands of Fragments A and B (both at 238 nm) agree closely with the experimental value (241 nm), while the maximum is underestimated for H_2_BDC (229 nm). The intense band is only 0.04 eV lower in energy in Fragment A than in Fragment B. The close agreement between the line shape and peak position of Fragments A and B, and to some extent H_2_BDC, strongly indicates that the intense bands and low-lying states are contributed solely by the BDC linker in MOF-5. All three models have low oscillator strength excitations (*f* > 0.01) in the region of 251 to 277 nm (4.47 to 4.93 eV) for the low-lying excited states (see Table S10, †ESI), and narrow broadening was chosen to avoid obscuring them. The first intense band (*f* > 0.3) at 4.85 to 5.42 eV (229 to 256 nm) dominates the absorption maximum. Although these states contribute low oscillator strength at the Franck–Condon geometry, they may be vibrationally activated to better capture the long absorption tail. The low-strength excitations lie close in energy to the BSE-GW optical gap (4.5 eV) reported previously.^66^ For Fragment A, the low oscillator strength excitation (*f* = 0.025) arises from S_1_, while the intense band (*f* = 0.79) comes from S_2_. In contrast, for Fragment B, the first band (*f* = 0.035) arises from S_2_, and the bright band from S_4_ (*f* = 0.83). It therefore follows that absorption is dominated by a ligand-centered (LC) excitation.

This conclusion is supported by the visualization of the excited-state density difference (Figure S7, see †ESI). For each model, the excitations are localized on the BDC linker and are qualitatively similar in nature. This remains true for H_2_BDC, despite a different truncation procedure from the crystal (i.e., a terminal carboxyl group). In Fragment B, the only model with multiple BDC units, excitations are seen on two of the BDC units, with small density on the first for S_2_, indicating some exciton formation and concomitant stabilization of the state. The excitonic coupling between the BDC portions of the molecule is small (20 meV by the half-gap rule). The LC character of the excitations is unsurprising, given that MOF-5 is Zn-based (d^10^), and consequently, the metal center does not participate in the excited states of the framework. LMCT, MLCT, or MC excitations are unlikely to be significant.^62,67^ An LLCT mechanism also appears unlikely, as the introduction of new linkers in Fragment B does not produce a bright charge transfer excitation in any of the functionals studied, suggesting that the results are converged with respect to model size. The other functionals studied (*vide infra*) showed qualitatively similar absorption spectra, but only the other range-separated hybrid, ωB97X-D, showed similar agreement in peak position.^9^

In the vibrational-broadened spectra, the improvement from H_2_BDC to the experimental spectrum is most notable, with the absorption maximum shifted to 239 nm, much closer to that of the larger MOF fragment models. The spectra of Fragments A and B are also slightly shifted to 242 nm. In all models, the low-lying states show a significant increase in oscillator strength, contributing primarily to the long-wavelength tail. The line shape of Fragment B is broader due to the much wider range of displacements (i.e., the number of vibrational modes) present. The simulations of absorption spectra for the models in vacuum clearly show that the lower-energy excitations are localized on the BDC linker. Because of this, we used only Fragment A to assess the effect of the crystal environment on the absorption spectrum.

The absorption maximum at the periodic DFT geometry is red-shifted compared to the experimental data, whereas the ONIOM S_0_ structure shows excellent agreement, similar to the vacuum calculation. This holds regardless of charge redistribution. Across all PCE, the periodic DFT geometry absorption maxima range from 245 to 246 nm, whereas the ONIOM spectra are placed between 238 and 241 nm, in line with the experimental maximum of 241 nm. Interestingly, both structures capture the broadening well, and better than the Fragment A model in vacuum. The improvement following optimization is largely independent of the charge scheme. This likely stems from the nature of the bond at the C–C carbon in the carboxyl region of the BDC linker. Although charge density is delocalized over these sp^2^-hybridized carbons, the bond is very neutral, and the M_1_ charge is small, resulting in minimal overpolarization. The high porosity of the framework means the remaining charges do not provide a large perturbation to the QM wave function energy. This is similar to what was observed in models C_23_ and C_34_, where a small M_1_ charge can result in good accuracy, despite charges close to the boundary. This is akin to what was found in QMOF-d29cec2.

In MOFs with a much denser structure, both the restriction of torsional motion available to its secondary building units and proximity to the charge distribution would suggest a larger influence on the QM region by the mechanical and electrostatic embedding, respectively. However, in MOF-5, the breaking of conjugation at the organic–inorganic interface and the lack of strong excitonic coupling between subunits suggest that the local relaxation Franck–Condon geometry is a reasonable approximation. In particular, the high porosity of the framework means that many of these groups are relatively isolated from the rest of the framework. Consequently, the constraints imposed by the environment center around critical coordinates (i.e., the bond between the linker and metal node) at which the local and wider environments are joined. Previously, vacuum cluster models have been used to model the photoisomerization of the linker unit in PCN-123, a derivative of MOF-5 in which the BDC linker is functionalized with azobenzene. In PCN-123, the large pores do not significantly restrict the range of motion available to the linker, and therefore, an isolated cluster model is justified.^68^ Additionally, in models C_23_ and C_34_ in diC_4_–BTBT, a small M_1_ charge can result in minimal overpolarization and good performance of a model independent of charge redistribution. Moreover, in the case of MOF-5, mechanical embedding dominates, and overpolarization is minimal. Consequently, MOFs with greater flexibility in the underlying scaffold, smaller pores, or large charge density on the building unit may show larger discrepancies between isolated cluster and embedded cluster calculations.

The success of the nuclear-ensemble approach highlights the molecular nature of MOFs. In conventional (ionic) solids, vibrational modes in crystals are characterized by the collective vibration of the lattice (phonons).^69^ Although phonons are crucial to many MOF processes,^70,71^ such as the breathing of pores in the low-frequency range, our results indicate that high-frequency vibrations (bond-stretching, wagging, etc.) can profoundly influence the excited states and be leveraged in local cluster models. Mapping protocols between phonon and local vibrational analysis are indeed interesting and applicable to molecular crystals. For instance, certain propeller-shaped blue emitters, displaying AIE, have been effectively characterized in the past through local vibrations.^72^ Consequently, we have demonstrated that cluster models can provide critical insight into the vibronic coupling of MOF solids.

#### 3.4.2 S_1_ Minimum

Finally, we investigate the emission spectrum of MOF-5 by relaxing the S_1_ state. Following Kasha’s rule,^73^ radiative fluorescent emission (S_1_ → S_0_) is expected to occur from the lowest-energy singlet state, S_1_. MOF-5 is Zn-based, suggesting that spin–orbit coupling is not significant, and therefore, phosphorescence is not expected to contribute to the photoluminescence spectrum. The localization of excited states on SBUs suggests that characteristics similar to molecular crystals should be observed, where embedded cluster models have previously shown the localization of the emissive excited state on a single molecule, even if absorption is initially delocalized.^30^ Experimentally, the maximum has been reported at 375 nm^65^ (3.31 eV) and 355 nm^67^ (3.49 eV), with the peak position varying depending on the quality of the MOF-5 crystal, which is notoriously difficult to obtain. We ensure that the activated crystal is modeled as per Villemot et al., and plot both spectra for clarity.

Table 1 reports S_1_ minima calculated at a range of levels of theory (TDDFT, MRSF-TDDFT, ADC(2), ONIOM) for our cluster models, as well as periodic TDA calculations on the MOF crystal. In the TDDFT/cc-pVDZ calculations, a strong dependence on the functional is observed, with significant differences between the range-separated and global hybrid functionals. For the H_2_BDC ligand, TD-PBE0 and TD-B3LYP optimizations predict S_1_ minima at 3.10 and 3.28 eV, respectively, which are red-shifted by more than 0.5 eV compared to the range-separated hybrids which place the S_1_ minimum at 3.77 eV. Visualization of the S_1_–S_0_ density differences (Figure 11) reveals that the lower energy S_1_ minima have *n*π* character about the carbonyl group; the state is dark because the transition is symmetry-forbidden.

**Table 1:** Vertical Excitation Energies (eV) and Oscillator Strengths in Parentheses (a.u.) at the S_1_ Minimum Following Excited-State Geometry Optimization^a^

System	B3LYP	PBE0	ωB97X-D	CAM-B3LYP	ADC(2)
H_2_BDC	3.10 (0.000)	3.22 (0.000)	3.77 (0.000)	3.77 (0.000)	4.37 (0.031)
	3.15 (0.000)	3.20 (0.000)	–	3.90 (0.130)	–
Fragment A	4.07 (0.024)	4.07 (0.027)	4.41 (0.031)	4.42 (0.031)	4.33*
	3.67 (0.160)	3.83 (0.148)	–	3.82 (0.137)	–
ONIOM-Z3	4.16 (0.023)	4.29 (0.026)	4.49 (0.030)	4.48 (0.032)	4.48**
Fragment B	2.89 (0.000)	3.22 (0.000)	4.36 (0.030)	4.40 (0.029)	-
Periodic	4.18 (0.010)	4.30 (0.009)	–	4.48 (0.004)	–

a Where calculated, MRSF-TDDFT values are given in the row below in italics. TDDFT calculations used the cc-pVDZ basis set. *ADC(2)/def-SVP for Fragment A. **ADC(2)/def2-SVP at PBE0S_1_ min.

**Figure 11:**
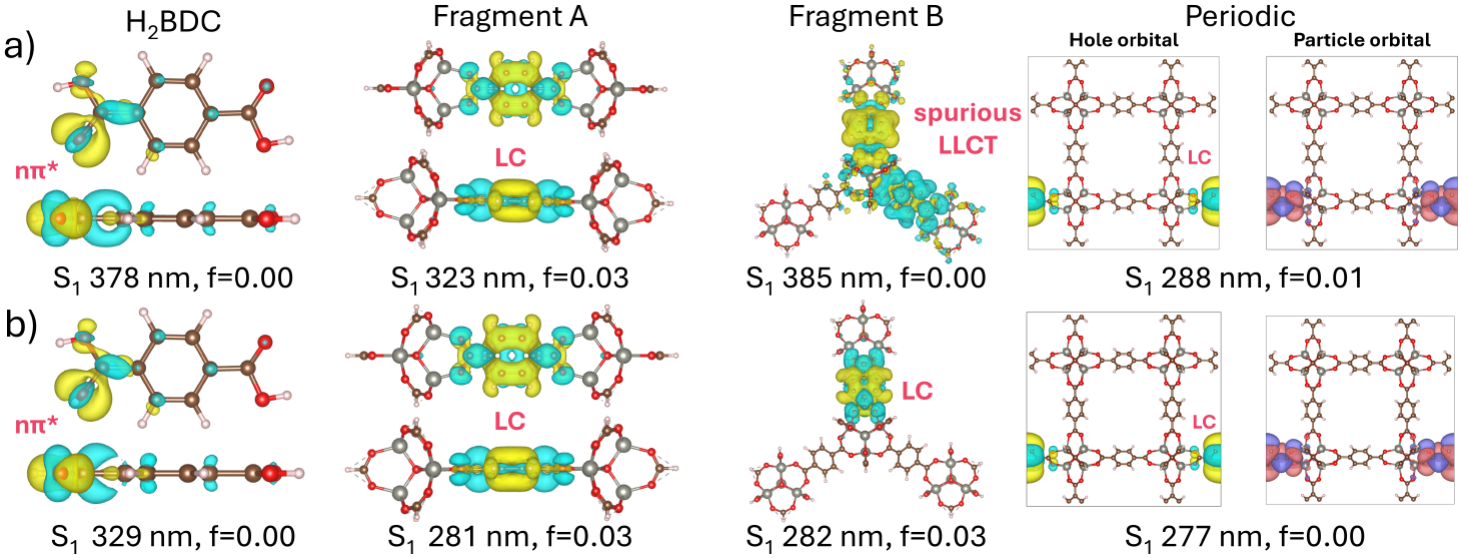
Visualizations of S_1_–S_0_ density difference at the S_1_-optimized geometry for a) PBE0 and b) CAM-B3LYP for each cluster model. The cc-pVDZ/LANL2DZ basis set was used for the TDDFT calculations. For the periodic TDA-PBE0 and TDA-CAM-B3LYP S_1_ minimizations, the DZVP-MOLOPT-SR/admm-dzp basis set and GTH-PBE pseudopotential were used. The dominant hole and electron NTOs are shown for the minimized structures.

In contrast, TDDFT on Fragment A indicates S_1_ is an LC excitation with small oscillator strength and ππ* character, at 4.07 eV for the global hybrids and 4.41–4.42 eV for the range-separated functionals. For Fragment B, a significant split is observed: TD-B3LYP and TD-PBE0 predict a dark LLCT state at 2.89 and 3.22 eV, respectively, while CAM-B3LYP and ωB97X-D weakly predict a bright LC (ππ*) state at 4.36 and 4.40 eV. The demanding size of Fragment B made converging a single-point ADC(2)/def2-SVP challenging, however a preoptimized calculation (i.e., an uncertainty of 0.02–0.03 eV) placed S_1_ at 4.37 eV at the B3LYP geometry, suggestive of the spurious nature of the LLCT state. It is well established that global hybrid functionals tend to underestimate the energies of charge-transfer (CT) states.^16^ Previously, TDDFT calculations with BLYP (i.e., a GGA functional with no exchange whatsoever) reported an LLCT emission mechanism in MOF-5.^74^ Finally, a fourth cluster model (Fragment C, see †ESI) containing the square pore structure (4 nodes + 4 linkers), and therefore retaining some of the geometric restraints from the actual framework, gives energies in the range 4.08–4.42 confirming that the size of our models is converged with respect to the crystal.

Indeed, the S_1_–S_0_ density differences are qualitatively similar to those obtained for absorption (Figure S6, see †ESI) However, these S_1_ minima are approximately 0.4 eV more stable than the FC geometry and much higher in energy than experiment, suggesting that the emissive S_1_ geometry may not have been reached and that the optimized structure may correspond to a different local minimum on the surface. In all cases, absorption was delocalized but largely uncoupled. For the range-separated hybrids, this result is consistent with observations for emissive states in molecular crystals, where the excitonic state localizes on a single molecule during relaxation.^31^ This corroborates the predictions of higher quality functionals and suggests that the contribution of double excitations is small, a conclusion also supported by experimental evidence for an LC mechanism.^67^ Villemot et al. showed that MOF-5 solvated in DMF, DMSO, and DCM exhibits similar peak positions (342–363 nm) and vibronic structure to H_2_BDC dissolved in these solutions, strongly suggesting that emission arises from a single excitation on the linker, regardless of the solvation environment.^67^

The ADC(2) minima for all models lie in the range 4.33–4.39 eV, suggesting a common excited state (LC) has been obtained in all cases. For H_2_BDC, the ADC(2) S_1_ state is higher in energy with respect to the TDDFT results, however, the small oscillator strength (*f* = 0.03) suggests this S_1_ state has different character. For Fragments A and B the ADC(2) calculations exclusively corroborate the range-separated hybrid results, indicating results from these functionals, and indeed these S_1_ minima, are the most reliable, at least when calculating using a single reference method, despite the blue-shift with respect to experiment. Notably, the energies of the LC states agree very well with the periodic TDA results, which place weakly bright (*f* = 0.004–0.01) emission minima at 4.30 eV for TDA-PBE0 and 4.48 eV and TDA-CAM-B3LYP. An NTO analysis shows the minimum is LC in nature. These results lie in markedly close in agreement with our excited-state ONIOM-EE optimizations at the TDDFT:xTB level for each functional. This consistency is a strong indicator that the ONIOM model is accurate. Indeed, the closer agreement with the periodic reflects that the ONIOM model, unlike the vacuum clusters, is mechanically and electrostatically coupled to the crystal. Charge transfer is inherently related to the proximity of the interacting fragments,^75^ and the restriction of the geometry optimization by the periodic arrangement is critical in the accessibility of these states. This is especially true for LLCT states, where the linkers are most spatially separated. As a result, LLCT is not accessible with global hybrids in the periodic calculations either, as the constraints of the solid prevent the ligands from approaching closely enough to stabilize the LLCT state. Moreover, this confirms that any disagreement with the experiments is not related to the use of cluster models or the quality of the embedding, but to the level of theory itself.

Consequently, to account for multireference character of the wave function, we consider Mixed Reference Spin-Flip TDDFT (MRSF-TDDFT), a method derived from SF-TDDFT with the significant advantage that the response states are spin-pure due to the use of a mixed reference, providing a good balance of dynamical and nondynamical correlation.^76,77^ MRSF-TDDFT calculations for H_2_BDC predict that the minima for PBE0 and B3LYP (3.15–3.20 eV) are similar to TDDFT. However, CAM-B3LYP shows a minimum at 3.90 eV with much larger oscillator strengths (0.130), suggesting this state is no longer *n*π*. For fragment A, MRSF-TDDFT predicts S_1_ minima in the range 3.72 to 3.82 eV with appreciable oscillator strength, much closer to the experimental value of 3.49 eV. The main transition (around 90% across all levels of theory) is associated with a HOMO–1 to LUMO electronic excitation, with a minor contribution from HOMO to LUMO+1, all of which lie predominantly on the linker. This suggests that multireference effects may influence the prediction of emission in MOF-5, and the triplet reference state used in MRSF-TDDFT provides a better reference for the excited states of MOF-5. This is also consistent with previous calculations on the H_2_BDC ligand in the crystalline phase, where CASPT2 showed better agreement with experiments than TD-ωB97X-D.^78^ Additionally, this excitation is localized on a portion of the molecule absent in MOF-5, raising doubts about the validity of this emissive state in the MOF crystal. Indeed, H_2_BDC, shows remarkable agreement with experiment, despite being, in principle, the least faithful approximation to the true MOF-5 crystal. We considered whether, due to the complexity of MOF synthesis, the error could lie in the characterization of the material, for instance, the presence of defects or unreacted H_2_BDC in the MOF-5 cages. However, our experimental reference comes from studies specifically focusing on the purity and quality of the crystals.^65,67^

#### 3.4.3 Emission Spectra

Having extensively characterized the emissive minimum, we use the NEA to compute the emission spectrum for H_2_BDC, Fragment A, and Fragment B with TDDFT and MRSF-TDDFT (Figures 12 and S11, see †ESI). A new interface between Newton-X^63^ and OpenQP^79^ was implemented to enable the simulation of the electronic spectra using the NEA, including multireference character. Vibrational broadening is particularly important, as displacement along normal modes allows the selection rules to be relaxed by breaking symmetry, enabling dark states to become bright.

**Figure 12:**
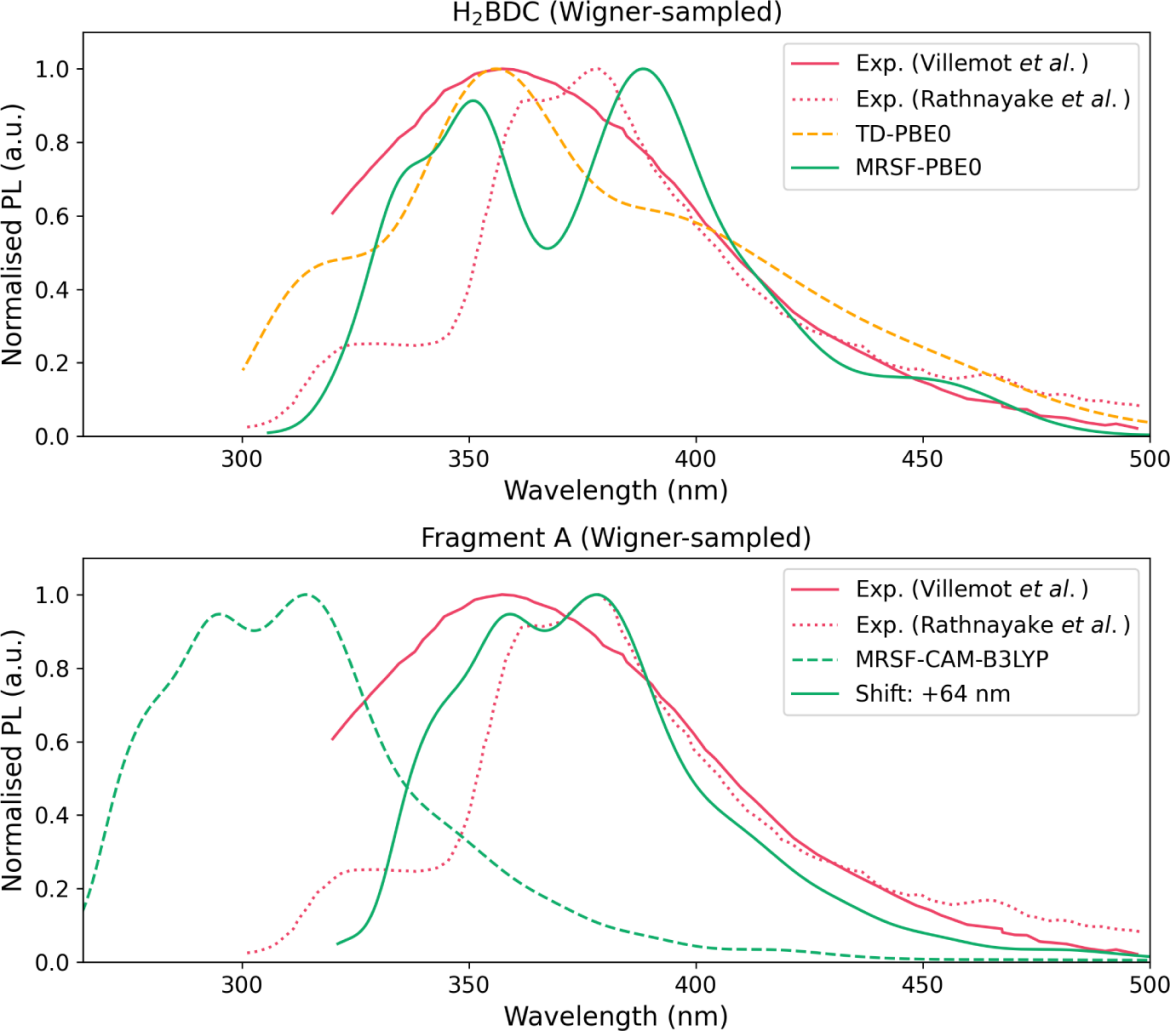
Emission spectra computed from the NEA: (top) H_2_BDC using TD-PBE0.cc-pVDZ and MRSF-PBE0/cc-pVDZ; (bottom) Fragment A with MRSF-CAM-B3LYP/def2-SVP. The shifted spectrum is also shown Two reference spectra from experiments are also plotted (refs 67 and 65).

First, for H_2_BDC, the TD-PBE0 and MRSF-PBE0 spectra once again show remarkable agreement with the experimental spectra, reflecting the fact that emission is attributable to the organic linker rather than the metal node. The emission peaks at 367–369 nm coincide very well with the experimental reference at 375 nm, although they are slightly blue-shifted compared to the broader reference at 355 nm. The CAM-B3LYP emission spectra for H_2_BDC (Figure S11, see †ESI) are blue-shifted, similar to the MOF fragments.

The spectrum for Fragment A with MRSF-CAM-B3LYP are the most interesting. The emission energy is overestimated by around 0.6 eV, however, the spectrum shows exceptional agreement in the line shape, capturing the broad emission tail to longer wavelengths, as well as the shoulder at the higher end of the spectrum. Consequently, we are confident that the emission in MOF-5 follows an LC mechanism, although some refinements in the level theory (e.g., larger basis, DFT functional, many-body effects) are likely required for better agreement in the vertical excitation energies. Indeed, we also computed the emission for Fragment B at the TDDFT level, as MRSF-TDDFT was prohibitively expensive for such a large cluster. Both TD-CAM-B3LYP and TD-PBE0 exhibit sharp emission peaks from the LC state, at 281 and 301 nm. However, TD-PBE0 also shows a second broad emission peak from 330 to 450 nm, corresponding to the LLCT excitation. This ostensibly shows remarkable agreement with experiment, however, this is an artifact of the functional, and is unlikely to be a dominant radiative pathway in the true MOF-5 crystal.

## 4 Conclusions

In this work, we have systematically studied the cluster and embedded cluster approaches for simulating the excited states of crystalline materials, spanning weakly- to strongly bound crystals, from molecular crystals to MOFs. Through this survey, we have illustrated the tremendous complexity of excited states in the condensed phase, as well as the frequent ability of very small models to capture the key characteristics. In particular, we have leveraged electrostatic embedding and implemented a new ONIOM­(QM:QM’)-EE method in fromage, enabling rapid deployment in crystal studies requiring bond cutting. This has allowed us to analyze the long-range dependency on the crystal environment.

First, diC_4_–BTBT illustrated the extent of the perturbation by the environment, depending on the electrostatic treatment near the QM:QM’ boundary. It is well-known in hybrid QM:QM’ studies that overpolarization by unphysical electrostatics at the boundary critically influence the excited-state energies. By cutting along the aliphatic group, we showed both the model itself but also the size of the M_1_ charge closest to the boundary, both play critical roles in the accuracy of the calculated excited-state energies and oscillator strengths. In all cases, electrostatic embedding was imperative to achieve results comparable to the full ONIOM reference. The well-established Z-*N* charge redistribution schemes were most effective for excited-states to remove overpolarizing point charges at the QM:QM’ boundary. The RC and RCD schemes performed less well than we expected for excited states, likely due to a lack of tuning in the redistribution point, here fixed as the midpoint of the M_1_-M_2_ bond. These results generalized across a range of common wave function (HF, CC(2)) and density functional (GGA, PBE0) methods, and basis sets (STO-3G, 6–31G**, TZVP, def2-SVP).

Second, polythiophene demonstrated the limits of electrostatic embedding, focusing on model construction over the perturbation by the environment. In highly delocalized systems, ensuring a model contains the relevant electronic structure is challenging. Cluster models are best suited to systems with coherent hopping transport, and therefore polythiophene, a semiconductor, illustrated the point at which the cluster model approach breaks down. At this limit, intramolecular effects strongly stabilize the singlet excited states due to through-bond conjugation. Additionally, incorporation of intermolecular effects via adjacent chains influenced the state, but our large aggregate model showed that the dominance of intra-over intermolecular effects outweighed the effect of PCE. This has strong implications for studies on (band-)­conductive MOFs.

Third, QMOF-d29cec2 extended the study to MOFs, facilitating direct comparison between periodic TDA-DFT on the conventional cell, 2 × 2 × 2 supercell, and a molecule-in-a-box. We revealed that in this hypothetical material, there is virtually no excitonic coupling. This enables excellent agreement between our truncated molecular models and the fully periodic solid, reflecting findings in the field of MOF excited states.^11,12^ Indeed, the agreement is such that periodic embedding provides only a small perturbation to the excited states. For highly porous MOFs, the benefits of the embedded cluster model, relative to the time taken to build such a model, are limited. However, in MOFs with a denser unit cell and smaller pores than QMOF-d29cec2, a larger effect is anticipated. Diabatization schemes for direct calculation of these couplings may prove an exciting avenue for future research.^75,80^

Finally, with the understanding of cluster and embedded cluster simulations built thus far, we investigated the absorption and emission spectra of MOF-5. Obtaining pristine MOF-5 crystals is experimentally challenging (e.g., defect concentration, purity, and solvation effects), introducing ambiguity in both experimental and theoretical characterization of absorption and emission. In this work, we have shown through three models that at least three linkers were required for good recovery of the first absorption band. We report absorption spectra of MOF-5 with vibrational broadening from Wigner-sampling, using both clusters in vacuum and in the crystalline phase through the use of ONIOM embedded cluster calculations, which also reproduce the first absorption band well. From these results, we draw a unique comparison to molecular crystals, where the localization of excited states and dominance of local high-frequency vibrations lead to excellent agreement with both our calculated spectra and the experimental data. Similarly, the relationship between phonon and molecular vibrations will be the subject of future work.

Additionally, we report S_1_ minima to investigate the emission spectra for MOF-5. Periodic TDA and ONIOM calculations show close agreement, providing strong evidence that we have built a consistent and accurate framework for modeling emission in MOF-5, and that it indeed follows an LC mechanism. However, vertical excitation energies at the emissive state are consistently overestimated, with ADC(2) calculations corroborating our range-separated functional calculations, suggesting that double excitations do not play a significant role in the emission process. In our larger models, TD-PBE0 and TD-B3LYP predict a spurious LLCT state in Fragment B, emphasizing the need for using range-separated functionals in MOF calculations, something that is essential in fundamental band gap predictions.^9,62^ Our periodic and ONIOM calculations show that mechanical constraints of the crystal environment, render this CT state inaccessible. Quantum chemical calculations will always critically depend on error cancellation, and in MOFs, the high degree of complexity merits careful selection of the DFT functional. From this perspective, we recommend the use of long-range corrected functionals, where possible, in the study of MOFs, but, as usual in density functional studies, it is imperative to benchmark the functional. Best results were obtained when including multireference effects. Good agreement with the experiment was found in just the H_2_BDC linker using MRSF-TDDFT calculations, and in our optimizations of fragment A.

Moreover, these final endeavors into emission in MOF-5 highlight the challenges and benefits of using cluster and embedded cluster models in condensed-phase excited-state studies. Across all of the systems studied, we have built a heuristic framework illustrating the challenges and solutions to recover these excited states in molecular crystals, conjugated polymers, and MOFs. In future work, we intend to apply this protocol to MOFs with more exotic photophysical and photochemical properties.

## Supplementary Material



## Notes

Additional theoretical descriptions and embedding excited-state calculations are provided. The dependence of point charge embedding on the basis set and level of theory, as well as the energy gaps for all employed schemes, is included. Specific results are presented for polythiophene embedded cluster calculations, QMOF-d29cec2, and MOF-5, along with additional results for the S_1_ minimum of H_2_BDC and a squared MOF-5 model (PDF)

## References

[1] Rivera M., Dommett M., Crespo-Otero R. (2019). ONIOM (QM: QM’) ElectrostaticEmbedding
Schemes for Photochemistry in Molecular Crystals. J. Chem. Theory Comput..

[2] Rivera M., Dommett M., Sidat A., Rahim W., Crespo-Otero R. (2020). fromage: Alibrary
for the study of molecular crystal excited states at the aggregatescale. J. Comput. Chem..

[3] Viglianti L., Leung N. L. C., Xie N., Gu X., Sung H. H. Y., Miao Q., Williams I. D., Licandro E., Tang B. Z. (2017). Aggregation-induced
emission: mechanistic study of the clusteroluminescence of tetrathienylethene. Chem. Sci..

[4] Liu J., Zhang H., Hu L., Wang J., Lam J. W. Y., Blancafort L., Tang B. Z. (2022). Through-Space Interaction of Tetraphenylethylene:
What,Where, and How. J. Am. Chem. Soc..

[5] Gomes A. S. P., Jacob C. R. (2012). Quantum-chemical
embedding methods for treatinglocal
electronic excitations in complex chemical systems. Annu. Rep. Prog. Chem., Sect. C: Phys. Chem..

[6] Hendon C. H., Rieth A. J., Korzyński M. D., Dincă M. (2017). Grand Challengesand
Future Opportunities for Metal-Organic Frameworks. ACS Cent. Sci..

[7] Böckmann M., Doltsinis N. L. (2015). Can Excited Electronic States ofMacromolecules
with
Extended Pi-Systems be Reliably Predicted?A Case Study on P3HT. Front. Mater..

[8] Panda J., Singha D., Panda P. K., Chandra Tripathy B., Rana M. (2022). K.Experimental and DFT Study of Transition
Metal Doping in aZn-BDC
MOF to Improve Electrical and Visible Light AbsorptionProperties. J. Phys. Chem. C.

[9] Ingham M., Aziz A., Di Tommaso D., Crespo-Otero R. (2023). Simulating
excitedstates in metal organic frameworks: from light-absorption to
photochemicalCO2 reduction. Mater. Adv..

[10] Mancuso J. L., Mroz A. M., Le K. N., Hendon C. H. (2020). Electronic StructureModeling
of Metal-Organic Frameworks. Chem. Rev..

[11] Ortega-Guerrero A., Fumanal M., Capano G., Tavernelli I., Smit B. (2020). Insightsinto the Electronic Properties
and Charge Transfer Mechanism
of aPorphyrin Ruthenium-Based Metal-Organic Framework. Chem. Mate.r.

[12] Ortega-Guerrero A., Fumanal M., Capano G., Smit B. (2020). From IsolatedPorphyrin
Ligands to Periodic Al-PMOF: A Comparative Study ofthe Optical Properties
Using DFT/TDDFT. J. Phys. Chem. C.

[13] Fumanal M., Corminboeuf C., Smit B., Tavernelli I. (2020). Optical absorptionproperties
of metal-organic frameworks: solid state versus molecularperspective. Phys. Chem. Chem. Phys..

[14] Li J., Ott S. (2024). The Molecular Nature of Redox-ConductiveMetal-Organic Frameworks. Acc. Chem. Res..

[15] Hernández F. J., Crespo-Otero R. (2021). Excited state mechanisms in crystallinecarbazole:
the
role of aggregation and isomeric defects. J.
Mater. Chem. C.

[16] Tsuneda T., Hirao K. (2014). Long-range correction
for density functional theory. Wiley Interdiscip.
Rev.: Comput. Mol. Sci..

[17] Sidat A., Ingham M., Rivera M., Misquitta A. J., Crespo-Otero R. (2023). Performance of point charge embedding schemes for excited
states in molecularorganic crystals. J. Chem.
Phys..

[18] Hernández F. J., Crespo-Otero R. (2023). Modeling Excited
States of MolecularOrganic Aggregates
for Optoelectronics. Annu. Rev. Phys. Chem..

[19] Hidalgo-Rosa Y., Mena-Ulecia K., Treto-Suárez M.
A., Schott E., Páez-Hernández D., Zarate X. (2022). Expanding the Knowledge
of theSelective-Sensing Mechanism of Nitro Compounds by LuminescentTerbium
Metal-Organic Frameworks through Multiconfigurational abInitio Calculations. J. Phys. Chem. A.

[20] Kühne T. D., Iannuzzi M., Ben M. D., Rybkin V. V., Seewald P., Stein F., Laino T., Khaliullin R. Z., Schütt O., Schiffmann F. (2020). CP2K: An electronic
structure and moleculardynamics software package - Quickstep: Efficient
and accurate electronicstructure calculations. J. Chem. Phys..

[21] Chai J.-D., Head-Gordon M. (2008). Long-range
corrected hybrid density functionalswith
damped atom-atom dispersion corrections. Phys.
Chem. Chem. Phys..

[22] Levine B. G., Coe J. D., Martínez T. J. (2008). Optimizing ConicalIntersections without
Derivative Coupling Vectors: Applicationto Multistate Multireference
Second-Order Perturbation Theory­(MS-CASPT2). J. Phys. Chem. B.

[23] Crespo-Otero R., Barbatti M. (2018). Recent Advances and Perspectives onNonadiabatic Mixed
Quantum-Classical Dynamics. Chem. Rev..

[24] Hourahine B., Aradi B., Blum V., Bonafé F., Buccheri A., Camacho C., Cevallos C., Deshaye M. Y., Dumitrică T., Dominguez A. (2020). DFTB+, a software package
for efficientapproximate density functional theory based atomistic
simulations. J. Chem. Phys..

[25] Bannwarth C., Ehlert S., Grimme S. (2019). GFN2-xTB-An Accurate andBroadly Parametrized
Self-Consistent Tight-Binding QuantumChemical Method with Multipole
Electrostatics andDensity-Dependent Dispersion Contributions. J. Chem. Theory.Comput..

[26] Dapprich S., Komáromi I., Byun K. S., Morokuma K., Frisch M. J. (1999). A newONIOM
implementation in Gaussian98. Part I. The calculation ofenergies,
gradients, vibrational frequencies and electric field derivatives. J. Mol. Struct.:THEOCHEM.

[27] Chung L. W., Hirao H., Li X., Morokuma K. (2012). The ONIOM method: itsfoundation
and applications to metalloenzymes and photobiology. WIREs Comput. Mol. Sci..

[28] Plett C., Katbashev A., Ehlert S., Grimme S., Bursch M. (2023). ONIOM meets
xtb: efficient, accurate, and robust multi-layer simulations across
the periodictable. Phys. Chem. Chem. Phys..

[29] Dommett M., Crespo-Otero R. (2017). Excited state
proton transfer in2’-hydroxychalcone
derivatives. Phys. Chem. Chem. Phys..

[30] Dommett M., Rivera M., Crespo-Otero R. (2017). How Inter-
and IntramolecularProcesses
Dictate Aggregation-Induced Emission in CrystalsUndergoing Excited-State
Proton Transfer. J. Phys. Chem. Lett..

[31] Dommett M., Rivera M. H., Smith M. T., Crespo-Otero R. (2020). Molecular
andcrystalline requirements for solid state fluorescence exploiting
excitedstate intramolecular proton transfer. J. Mater. Chem. C.

[32] Sidat A. J., Hernández F., Stojanović L.
J., Misquitta A., Crespo-Otero R. (2022). Competition between ultralong organic phosphorescence
andthermally activated delayed fluorescence in dichloro derivatives
of9-benzoylcarbazole. Phys. Chem. Chem. Phys..

[33] Sharma M., Sierka M. (2024). Optical Gaps of Ionic
Materials fromGW/BSE-in-DFT and
CC2-in-DFT. J. Chem. Theory Comput..

[34] Senn H. M., Thiel W. (2009). QM/MM Methods for Biomolecular
Systems. Angew.
Chem., Int. Ed..

[35] Chung L. W., Sameera W. M. C., Ramozzi R., Page A. J., Hatanaka M., Petrova G. P., Harris T. V., Li X., Ke Z., Liu F., Li H.-B., Ding L., Morokuma K. (2015). The ONIOM Method and
Its Applications. Chem. Rev..

[36] Lin H., Truhlar D. G. (2005). Redistributed Charge
and Dipole Schemes forCombined
Quantum Mechanical and Molecular MechanicalCalculations. J. Phys. Chem. A.

[37] Mayhall N. J., Raghavachari K. (2010). Charge Transfer Across ONIOM QM: QMBoundaries: The
Impact of Model System Preparation. J. Chem.
Theory Comput..

[38] Vreven T., Morokuma K. (2000). On the application
of the IMOMO (integratedmolecular
orbital + molecular orbital) method. J. Comput.
Chem..

[39] Mayhall N. J., Raghavachari K., Hratchian H. P. (2010). ONIOM-based QM: QMelectronic embedding
method using Löwdin atomic charges: Energies andanalytic gradients. J. Chem. Phys..

[40] Zhang K., Ren S., Caricato M. (2020). Multistate
QM/QM Extrapolation ofUV/Vis Absorption
Spectra with Point Charge Embedding. J. Chem.
Theory Comput..

[41] Biancardi A., Barnes J., Caricato M. (2016). Point charge embedding
for ONIOMexcited
states calculations. J. Chem. Phys..

[42] Seeber P., Seidenath S., Steinmetzer J., Gräfe S. (2023). Growing SpicyONIOMs:
Extending and generalizing concepts of ONIOM and many body expansions. Wiley Interdiscip. Rev.: Comput. Mol. Sci..

[43] Singh U. C., Kollman P. A. (1986). A combined ab initio quantum mechanical andmolecular
mechanical method for carrying out simulations on complex molecularsystems:
Applications to the CH3Cl + Cl- exchange reaction and gasphase protonation
of polyethers. J. Comput. Chem..

[44] Wu X.-P., Gagliardi L., Truhlar D. G. (2019). Multilink F* Method for CombinedQuantum
Mechanical and Molecular Mechanical Calculations ofComplex Systems. J. Chem. Theory Comput..

[45] Zhang J., Lu T. (2021). Efficient evaluation of electrostatic
potential withcomputerized
optimized code. Phys. Chem. Chem. Phys..

[46] Derenzo S. E., Klintenberg M. K., Weber M. J. (2000). Determining point chargearrays that
produce accurate ionic crystal fields for atomic clustercalculations. J. Chem. Phys..

[47] Klintenberg M., Derenzo S. E., Weber M. J. (2000). Accurate crystal fields forembedded
cluster calculations. Comput. Phys. Commun..

[48] Rivera M., Stojanović L., Crespo-Otero R. (2021). Role of Conical Intersectionson the
Efficiency of Fluorescent Organic Molecular Crystals. J. Phys. Chem. A.

[49] Wilbraham L., Adamo C., Labat F., Ciofini I. (2016). Electrostatic
Embedding
ToModel the Impact of Environment on Photophysical Properties ofMolecular
Crystals: A Self-Consistent Charge AdjustmentProcedure. J. Chem. Theory Comput..

[50] Presti D., Wilbraham L., Targa C., Labat F., Pedone A., Menziani M. C., Ciofini I., Adamo C. (2017). Understanding
Aggregation-Induced
Emission inMolecular Crystals: Insights from Theory. J. Phys.Chem. C.

[51] Luise D., D’Alterio M. C., Talarico G., Ciofini I., Labat F. (2022). Modeling thespectral
properties of poly­(x-phenylenediamine) conducting polymers using acombined
TD-DFT and electrostatic embedding approach. J. Comput. Chem..

[52] Su J., Luise D., Ciofini I., Labat F. (2023). Modeling the Electronic
andOptical Properties of Lead-Based Perovskite Materials: Insightsfrom
Density Functional Theory and Electrostatic Embedding.*The*. J. Phys. Chem. C.

[53] Minemawari H., Tanaka M., Tsuzuki S., Inoue S., Yamada T., Kumai R., Shimoi Y., Hasegawa T. (2017). Enhanced Layered-Herringbone
Packing due toLong Alkyl Chain Substitution in Solution-Processable
OrganicSemiconductors. Chem. Mater..

[54] Treß R. S., Hättig C., Höfener S. (2022). Employing Pseudopotentials toTackle
Excited-State Electron Spill-Out in Frozen DensityEmbedding Calculations. J. Chem. Theory Comput..

[55] Elliott P., Goldson S., Canahui C., Maitra N. T. (2011). Perspectives
ondouble-excitations
in TDDFT. Chem. Phys..

[56] Kobayashi M., Chen J., Chung T. C., Moraes F., Heeger A. J., Wudl F. (1984). Synthesis and properties
of chemically coupled poly­(thiophene). Synth.
Met..

[57] Fazzi D., Barbatti M., Thiel W. (2015). Modeling ultrafast
exciton deactivation
inoligothiophenes via nonadiabatic dynamics. Phys. Chem. Chem. Phys..

[58] Rosen A. S., Fung V., Huck P., O’Donnell C. T., Horton M. K., Truhlar D. G., Persson K. A., Notestein J. M., Snurr R. Q. (2022). High-throughput predictions of metal-organic framework
electronicproperties: theoretical challenges, graph neural networks,
and dataexploration. Npj Comput. Mater..

[59] Rosen A. S., Iyer S. M., Ray D., Yao Z., Aspuru-Guzik A., Gagliardi L., Notestein J. M., Snurr R. Q. (2021). Machine learning
the quantum-chemicalproperties of metal-organic frameworks for accelerated
materials discovery. Matter.

[60] Iannuzzi M., Chassaing T., Wallman T., Hutter J. (2005). Ground and ExcitedState
Density Functional Calculations with the Gaussian andAugmented-Plane-Wave
Method. Chimia.

[61] Prentice J. C. A., Mostofi A. A. (2021). Accurate and Efficient
Computation ofOptical Absorption
Spectra of Molecular Crystals: The Case ofthe Polymorphs of ROY. J. Chem. Theory Comput..

[62] Kshirsagar A. R., Blase X., Attaccalite C., Poloni R. (2021). Strongly BoundExcitons
in Metal-Organic Framework MOF-5: A Many-BodyPerturbation Theory Study. J. Phys. Chem. Lett..

[63] Barbatti M. (2022). Newton-X Platform: New SoftwareDevelopments
for Surface Hopping and
Nuclear Ensembles. J. Chem. Theory Comput..

[64] Crespo-Otero R., Barbatti M. (2012). Spectrum simulation
and decomposition withnuclear ensemble:
formal derivation and application to benzene, furan and2-phenylfuran. Theor. Chem. Acc..

[65] Rathnayake H., Saha S., Dawood S., Loeffler S., Starobin J. (2021). AnalyticalApproach
to Screen Semiconducting MOFs Using Bloch ModeAnalysis and Spectroscopic
Measurements. J. Phys. Chem. Lett..

[66] Kshirsagar A. R., D’Avino G., Blase X., Li J., Poloni R. (2020). AccuratePrediction
of the S1 Excitation Energy in Solvated AzobenzeneDerivatives via
Embedded Orbital-Tuned Bethe-SalpeterCalculations. J. Chem. Theory Comput..

[67] Villemot V., Hamel M. B., Pansu R., Leray I. V., Bertrand G. (2020). H.Unravelling
the true MOF-5 luminescence. RSC Adv..

[68] Kshirsagar A. R., Attaccalite C., Blase X., Li J., Poloni R. (2021). Bethe-Salpeter
Study of the Optical Absorption of trans and cisAzobenzene-Functionalized
Metal-Organic Frameworks UsingMolecular and Periodic Models. J. Phy. Chem. C.

[69] Togo A., Tanaka I. (2015). First principles phonon calculations
in materials science. Scr. Mater..

[70] Kamencek T., Bedoya-Martínez N., Zojer E. (2019). Understanding phonon
propertiesin isoreticular metal-organic frameworks from first principles. Phys. Rev. Mater..

[71] Hoffman A. E. J., Senkovska I., Abylgazina L., Bon V., Grzimek V., Dominic A. M., Russina M., Kraft M. A., Weidinger I., Zeier W. G. (2023). The role of phonons in switchable MOFs: Amodel
material perspective. J. Mater. Chem. A.

[72] Stojanović L., Crespo-Otero R. (2019). Understanding
Aggregation InducedEmission in a Propeller-Shaped
Blue Emitter. ChemPhotochem.

[73] Kasha M. (1950). Characterization
of electronic transitions in complex molecules. Discuss. Faraday Soc..

[74] Feng P. L., Perry J. J. I., Nikodemski S., Jacobs B. W., Meek S. T., Allendorf M. D. (2010). Assessing the Purity of Metal-Organic FrameworksUsing
Photoluminescence: MOF-5, ZnO Quantum Dots, and FrameworkDecomposition. J. Am. Chem. Soc..

[75] Green J. A., Asha H., Santoro F., Improta R. (2021). Excitonic Model forStrongly
Coupled Multichromophoric Systems: The ElectronicCircular Dichroism
Spectra of Guanine Quadruplexes as TestCases. J. Chem. Theory Comput..

[76] Park W., Komarov K., Lee S., Choi C. H. (2023). Mixed-Reference
Spin-FlipTime-Dependent Density Functional Theory: MultireferenceAdvantages
with the Practicality of Linear Response Theory.*The*. J. Phys. Chem. Lett..

[77] Horbatenko Y., Sadiq S., Lee S., Filatov M., Choi C. H. (2021). Mixed-ReferenceSpin-Flip
Time-Dependent Density Functional Theory­(MRSF-TDDFT) as a Simple yet
Accurate Method for Diradicals andDiradicaloids. J. Chem. Theory Comput..

[78] Stojanović L., Dommett M., Crespo-Otero R. (2025). Origins ofcrystallisation-induced
dual emission of terephthalic and isophthalic acidcrystals. Phys. Chem. Chem. Phys..

[79] Mironov V., Komarov K., Li J., Gerasimov I., Nakata H., Mazaherifar M., Ishimura K., Park W., Lashkaripour A., Oh M. (2024). OpenQP: A Quantum Chemical
Platform FeaturingMRSF-TDDFT with an Emphasis on Open-Source Ecosystem. J. Chem. Theory Comput..

[80] Green J. A., Yaghoubi Jouybari M., Asha H., Santoro F., Improta R. (2021). Fragment Diabatization
Linear Vibronic Coupling Model for Quantum Dynamics of Multichromophoric
Systems: Population of theCharge-Transfer State in the Photoexcited
Guanine-CytosinePair. J. Chem. Theory Comput..

