# Supplementary material for: Describing Excited States of Covalently Connected Crystals with Cluster and Embedded Cluster Approaches: Challenges and Solutions
Source: J Chem Theory Comput. 2025 Jul 24;21(15):7576–92. doi: 10.1021/acs.jctc.5c00539 (PMC12355689; doi:10.1021/acs.jctc.5c00539)
Supplement: Supplementary file 1 [file ct5c00539_si_001.pdf]

Supporting Information: Describing excited states of covalently connected crystals with cluster and embedded cluster approaches: Challenges and solutions

Michael Ingham, M. Brady, and Rachel Crespo-Otero
Department of Chemistry, University College London, 20 Gordon Street, London, WC1H 0AJ

1 Theoretical details

1.1 ONIOM equations

The position of link atoms are defined in terms of positions of the inner atom, the link atom connect (LAC), and outer atom, the link atom host (LAH), of the bond cut,

$$\mathbf{r}_{\mathrm{LA}} = \mathbf{r}_{\mathrm{LAC}} + g(\mathbf{r}_{\mathrm{LAH}} - \mathbf{r}_{\mathrm{LAC}}) \tag{1}$$

where $\mathbf{r}_{\mathrm{LA}}$ are the xyz-coordinates of the link atom, $\mathbf{r}_{\mathrm{LAC}}$ are the coordinates of the LAC (link atom centre), $\mathbf{r}_{\mathrm{LAH}}$ are the coordinates of the LAH (link atom hydrogen), and g is the scaling factor,

$$g = \frac{\mathrm{d}_1}{\mathrm{d}_2} \tag{2}$$

where d_1 is the distance between the LA and LAC, and d_2 is the distance between the LAC. We use the parameters from Ref. 1 to select d_1. By using Equation. 1 to define link atom positions, the ONIOM gradients are straightforward to calculate from each sub-calculation,

$$\frac{\partial E_{\mathrm{ONIOM}}(\mathbf{r}_{\mathrm{real}})}{\partial \mathbf{r}_{\mathrm{real}}} = \frac{\partial E_{\mathrm{QM}}(\mathbf{r}_{\mathrm{model}} + L)}{\partial(\mathbf{r}_{\mathrm{model}} + L)} \cdot \mathbf{J}(\mathbf{r}_{\mathrm{model}}+L; \mathbf{r}_{\mathrm{real}}) + \frac{\partial E_{\mathrm{QM'}}(\mathbf{r}_{\mathrm{real}})}{\partial \mathbf{r}_{\mathrm{real}}} - \frac{\partial E_{\mathrm{QM'}}(\mathbf{r}_{\mathrm{model}} + L)}{\partial(\mathbf{r}_{\mathrm{model}} + L)} \cdot \mathbf{J}(\mathbf{r}_{\mathrm{model}}+L; \mathbf{r}_{\mathrm{real}}) \tag{3}$$

Where J is a Jacobian matrix that projects the artificial link atom gradients on atoms in the real region. This must be performed to locate a true minimum in the PES, and we follow the standard definition,[1–3]

$$\frac{\partial \mathbf{r}_{\mathrm{LA}}}{\partial \mathbf{r}_{\mathrm{LAH}}} = g \tag{4}$$

$$\frac{\partial \mathbf{r}_{\mathrm{LA}}}{\partial \mathbf{r}_{\mathrm{LAC}}} = g - 1 \tag{5}$$

which holds when the link atom positions are defined according to Equation 1. Note, in our previous ONIOM studies on molecular crystals,[4–8] the QM:QM' boundary does not cut through covalent bonds, therefore the Jacobian was trivially taken as the unit matrix.[3]

2 Methods and computational details

2.1 Periodic geometry optimisations

The crystallographic atomic coordinates of each crystal were relaxed with a periodic DFT calculation to avoid errors in the cluster and embedded cluster models. For diC_4-BTBT (Space group: $P\bar{1}$),[9] the periodic geometry optimisation of S_0 was performed using the Vienna Ab initio Simulation Package (VASP),[10] with the PBE exchange-correlation functional, under fixed cell parameters. A GGA functional is used for reduced cost, and PBE gives acceptable coordinates for geometry optimisations at substantially lower cost than a hybrid. The plane-wave basis set had a cut-off energy of 520 eV. A Monkhorst-Pack k-point mesh of $3 \times 2 \times 1$ was used with Gaussian smearing of 0.01 eV. The DFT-D3 dispersion correction was used. The same settings were used for polythiophene (space group: $P\bar{1}$), but under a $2 \times 3 \times 2$ Monkhorst-Pack grid due to the different shape of the unit cell.

For MOF-5, periodic KS-DFT was used to relax the crystallographic coordinates for MOF-5 (Space group: Fm-3m) using the GPW method in CP2K.[11] The GPW method provides a better cost-accuracy trade-off required to study the much larger MOF unit cells. The PBE exchange correlation was used, and the TZVP-MOLOPT-PBE-GTH basis set for Zn atoms and DZVP-MOLOPT-SR-GTH for O, C, and H atoms. The D3 dispersion correction was added. The GTH-PBE pseudopotentials were used for all calculations. A primitive cell was used to reduce computational cost. An energy cutoff of 450 Ry was used and a relative cutoff of 60 Ry, and calculations were performed at the Γ-point only. The input files were generated using Multiwfn.[12]

2.2 diC_4-BTBT

First, diC_4-BTBT was used to gauge the influence of model size and charge distribution on the magnitude of overpolarisation in the embedded cluster models. Using single-point calculations on a common geometry, we tested a series of truncated models against the full ONIOM calculation. Firstly, the *real* region comprising 15 diC_4-BTBT molecules was generated from the relaxed unit cell using `fromage` including all molecules within the first coordinates sphere. The environment is modest in size (672 atoms), and serves as the exact theoretical reference against which all error deviations are calculated. We refer to it as the full ONIOM calculation as no link atoms were required. Taking this as a reference, rather than experiment, circumvents inherent limitations of the excited-state method (in this case TDDFT) and uncertainty in the experimental setup. The model region, a single diC_4 molecule was embedded in S_0 RESP charges, following the S_0 charge assignment scheme detailed in Section 2.2. Using this model, single-point TD-HF, TD-PBE, and TD-PBE0 calculations were performed in the STO-3G, 6-31G and TZVP basis sets to provide a set of exact theoretical references against which we could benchmark our ONIOM results. Additionally, CC(2)/def2-SVP calculations were performed. Of course, in the case of the standard crystal calculation, no charge redistribution is required as no bond cuts are made. By varying the content of HF exchange in the levels of theory (HF: 100%, PBE: 0%, PBE0: 25%) we cover a broad spectrum of exchange-correlation treatments, allowing us to assess the performance of different approaches in describing excitation energies and oscillator strengths on a single-reference, up to the inclusion of double excitations in CC(2).

Next, four truncated models were constructed by truncating the model region symmetrically at each alkyl chain. Bond cuts were made at the C_{12}, C_{23}, C_{34}, and C_{45} positions, thus creating systematically larger models (See Figure 1 in main text). The same electronic embedding was used as before. Point charges were redistributed according to the Z0, Z1, Z2, Z3, RC, and RCD schemes. Calculations were performed using TD-HF, TDDFT, CC(2) and basis sets STO-3G, 6-31G**, TZVP, def-SVP basos sets, as per the theoretical references. Single-point calculations indicate the quality of electronic embedding, as it decouples the electrostatic contribution to the excited states from the nuclear coordinates. Therefore, this comparison indicates the influence of charge redistribution. Moreover, this set of test models allows us to systematically investigate the influence of the size of the model region, the magnitude of the M_1 charge, and the charge scheme on the excited-state energies and oscillator strengths.

2.3 Polythiophene

Next, polythiophene is used to demonstrate the limitations of cluster models in the case of extended conjugation. Polythiophene is an interesting intermediate between molecular crystals and MOFs, as it has expanded connectivity in one dimension but does not divide the organic chain with metal nodes. Polythiophene is a low-band-gap semiconductor making cluster models challenging to use, although tetramer models have previously been used to describe excited states in nonadiabatic dynamics simulations.[13] As such we systematically investigate the polythiophene embedded cluster model by increasing the number of polythiophene units in the model region: 1, 2, 4, 5, and 8. The real region was taken to be the 10-unit monomer, extracted from the relaxed unit cell. In all cases, the models were extracted from the real region, and both real and model regions were saturated using hydrogen link atoms. Crucially, the QM/QM' boundary necessarily cuts C=C double bonds between neighbouring thiophene units, making it highly unfavourable, compared to the cuts in diC$_4$-BTBT. Point charges were obtained from obtained from a single-point PBE0/cc-pVDZ calculation on the real region. On these structures, single-point TD-ωB97X-D/cc-pVDZ (real-high) calculations were performed in the presence of both S_0 RESP and Mulliken charges using each charge redistribution scheme. Moreover, this study serves as an example where the underlying electronic structure of the model cannot be improved through electronic embedding alone.

To investigate the intermolecular effects, an aggregate model of four thiophene chains containing 9 units (Figure 4b in main text) was extracted from the unit cell. Similar to the previous model, subsets of this real region were extracted from the centre of each chain and according to the indices shown in Figure 4b. For example, the (3-5) model indicates that the embedded cluster model region contains 3 chains of five thiophene units each. This approach ensures a balanced distribution of point charges either side of the model. For each embedded cluster, the first 5 singlet excited states were calculated using TD-ωB97X-D/cc-pVDZ. Calculations were performed in vacuum and with Z2 charge redistribution.

2.3.1 QMOF-d29cec2

Next, the effect of electronic embedding on MOFs was studied under Ewald embedding.[5] As materials, MOFs are characterised in terms of their secondary building units (SBUs), metal nodes and multivalent organic

linkers, which combine to form an extended mesoporous framework.[14] Consequently, MOFs are similar to molecular crystals in that a MOF can be decomposed into combinations of SBUs, but are different in that these sub-units are connected by chemical bonds. Indeed, the modularity of the framework means truncated cluster models are often a very good approximation to the periodic crystal.[15,16] Like in molecular crystals, where dimer models (or higher-order oligomer) models might be required to describe some aggregation effects like charge-transfer, MOF cluster models *must* contain all relevant SBUs. In this respect, benchmarking the model against periodic calculations is useful. In a previous study,[17] we performed this analysis for QMOF-d29cec2, a theoretical MOF, where we showed that a cluster model containing a single Zn node, and three linkers was sufficiently large capture the fundamental band gap, and to recover the low-lying excited states. Here, we extend this by also considering Ewald embedding in our ONIOM implementation.

To generate the Ewald embedding, Mulliken, RESP, and REPEAT population analyses were performed from a single-point periodic DFT calculation using the PBE and PBE0 functionals, from the PBE-D3(BJ)-relaxed unit cell. The relaxed crystal structure was obtained from the QMOF database (space group: $P\bar{1}$). On this geometry, single-point periodic DFT calculations were performed using the GPW method. A plane wave cut-off of 450 eV and a relative cut-off of 50 eV and GTH-PBE pseudopotentials were used. The DZVP-MOLOPT-SR basis sets were used for all atoms. The PBE0 calculations were accelerated using the auxiliary density matrix method (ADMM), where the admm-dzp auxiliary basis set was used for all atoms. Following assignment of the periodic charges to the unit cell, $\approx$ 10,000 Ewald charges were generated to embed the QMOF-29cec2 cluster model. On this model, single-point TDA-ωB97X-D/cc-pVDZ calculations were performed. Here, the Tamm-Dancoff approximation (TDA) was used, rather than the full Casida,[18] to provide optimal agreement with our periodic calculations, where only TDA is available.[19]

Finally, to provide an exact comparison between periodic and cluster models, single-point periodic TDA-ωB97X-D calculations were performed at the crystal geometry on the $1 \times 1 \times 1$ unit cell, a $2 \times 2 \times 2$, and on the cluster model (without PCE) in a $27.1 \times 27.1 \times 27.1$ Å^3 using CP2K. The TZVP-MOLOPT basis set was used, except for Zn which employed DZVP-MOLOPT-SR. All calculations used the admm-dzp auxiliary basis set. For the latter, periodic boundary conditions were turned off and the wavelet Poisson solver was used to ensure zero electron density at the box edge, thereby providing a molecule-like electron density within the periodic TDA implementation. Otherwise, the same cut-offs as the population analysis were used, and the calculation was accelerated with ADMM.

2.3.2 MOF-5

In the final study, TDDFT was used to calculate vertical absorption energies and S_1 minima in MOF-5 in an isolated and embedded cluster model. First, three cluster models of MOF-5 that have been previously used in a study on absorption by Kshirsagar *et al.*: 1) the benzenedicarboxylic (BDC) acid linker (H_2-BDC); 2) 1 BDC linker unit and 2 metal nodes (Zn_4O); and 3) 4 nodes and 3 linkers.[20] In their study, many-body perturbation theory (MBPT) calculations showed the strong excitonic binding in MOF-5, resulting in a large difference in the optical and fundamental gaps than might be predicted with DFT alone. Notably, the PBE0/GW/BSE optical gap of 4.8 eV (257 nm) for Fragment B is in good agreement with the experimental

thin-film UV-vis absorption spectra,[21] with the other cluster models and periodic models 4.49 eV to 4.52 eV being shifted to longer wavelengths. As such, despite the poor performance of density functional methods in reproducing the fundamental gap, it is valuable to understand how TDDFT performs in modelling the optical gap of MOF-5, due to its significantly more favourable cost-accuracy trade-off.

From the relaxed periodic DFT geometry, the three cluster models were extracted: the H_2-BDC linker, Fragment A (1 linker + 2 nodes) and Fragment B (4 nodes + 3 linkers), capped with link atoms. For the vacuum calculations, the S_0 and S_1 states were optimised using Gaussian16 at the B3LYP, PBE0, CAM-B3LYP, and ωB97X-D in the cc-pVDZ basis set. For the B3LYP, PBE0 and CAM-B3LYP functionals, D3(BJ) dispersion was added. The LANL2DZ effective core potential (ECP) was used for Zn atoms to reduce computational cost. Tight threshold criteria were used to ensure a true minimum was identified. Subsequently, a high-precision frequency calculation was performed. For both S_0- and S_1-optimised structures, density difference plots were generated using `cubman`, to subtract ground- and excited-state electron densities. This was performed for S_1 to S_3. For each S_0 structure, a single-point TDDFT calculation was performed for the first 15 excited states to produce a stick spectrum for absorption.

For the embedded cluster calculations on MOF-5, an ONIOM model was constructed about fragment A. To obtain the real region, a much larger cluster centered at fragment A containing 20 metal nodes and 59 linkers (Figure 10b) was generated using an in-house `fromage` script. Point charge embedding was generated from a Mulliken population analysis provided by the real-low xTB calculation. The absorption spectrum was computed from: a) single-point TDDFT/cc-pVDZ calculations at the periodic DFT geometry using the first 15 excitations, embedding each calculation with charges redistributed according to each scheme. Next, S_0 optimisations were performed using ONIOM(DFT:xTB)-EE for the same functionals and basis, followed by a single-point ONIOM(TDDFT:xTB) calculation to compute the first 15 excitations. During the geometry optimisations, the values of each point charges were fixed but the positions were allowed to move. To incorporate vibrational broadening the same approach based on Wigner sampling was used as in vacuum and were performed for CAM-B3LYP at both the ONIOM-S_0 and relaxed crystal (i.e. non local optimisation) coordinates. For these few calculations, the single-point frequency calculation was performed essentially in the presence of point charges was performed was not being performed at a local minimum of the PES, therefore imaginary modes where necessarily found. Nevertheless, Wigner serves as an effective means to explore configurational space about the FC point, therefore we proceeded, neglecting contributions of any imaginary modes. Finally, S_1 optimisations at the ONIOM(TDDFT:xTB)-EE were performed for each functional in the cc-pVDZ basis set.

For absorption, two approaches were used to broaden the absorption spectra. Firstly, Gaussian broadening was used on the vertical excitations calculated at the Frank-Condon geometry for the single-point TDDFT calculation. The height of the peak was determined from the normalised oscillator strength. Secondly, the S_0 frequency calculation was used to generate 200 configurations for each vacuum structure according to a Wigner distribution (i.e., the nuclear ensemble approach, NEA), as implemented in Newton-X,[22,23] to ensure that each model is at the proper zero point level.[24] This analysis was performed for CAM-B3LYP as its Gaussian-broadened absorption spectrum showed best agreement with experiment. For each model,

Newton-X was used to calculate the vibrationally-broadened spectrum.

Finally, for the S_1 minimisation, MRSF-TDDFT optimisations were formed using OpenQP on H_2-BDC and Fragment A using the B3LYP, PBE0, and CAM-B3LYP functionals.[25,26] MRSF-TDDFT calculates the singlet states (including S_0) as response states of a mixed high- and low-spin triplet state calculated from a restricted open-shell calculation, thereby introducing multireference character into the TDDFT method. Consequently, multiconfigurational features, such as the topology of the surface at conical intersections, of the PES are correct. Unlike traditional spin-flip TDDFT, the singlet response states are spin-pure. Fragment B could not be studied with MRSF due to the demanding computational cost. Additionally, from the S_1 minima, we generate the MRSF-PBE0 emission spectra for H_2BDC using an interface with Newton-X. The S_1 vibrational modes used for the Wigner sampling were calculated numerically from finite-differences method, as implemented in the PyOQP Python wrapper. The details of this interface will be the subject of a forthcoming publication. The MRSF-CAM-B3LYP/def2-SVP (200 configurations) emission spectrum was also calculated via the NEA. The displacements at the MRSF minima were computed using normal modes from CAM-B3LYP-cc-pVDZ calculation.

Periodic TDA-TDFT (PBE0, B3LYP, CAM-B3LYP) geometry optimisations were performed at the crystal geometry on the $1 \times 1 \times 1$ unit cell using CP2K. Energies and gradients were calculated for S_1 at each optimisation step. Calclations were at the Γ-point only. The DZVP-MOLOPT-SR/admm-dzp basis set was used with the GTH-PBE pseudopotentials. The grid cut-off of 400 Ry with a relative cut-off of 55 Ry. All calculations were accelerated with ADMM. On the converged geometry, NTOs were calculated on grid for the dominant transitions of S_1 to S_3.

3 diC$_4$-BTBT embedded cluster models

3.1 Dependence of point charge embedding on basis set and level of theory

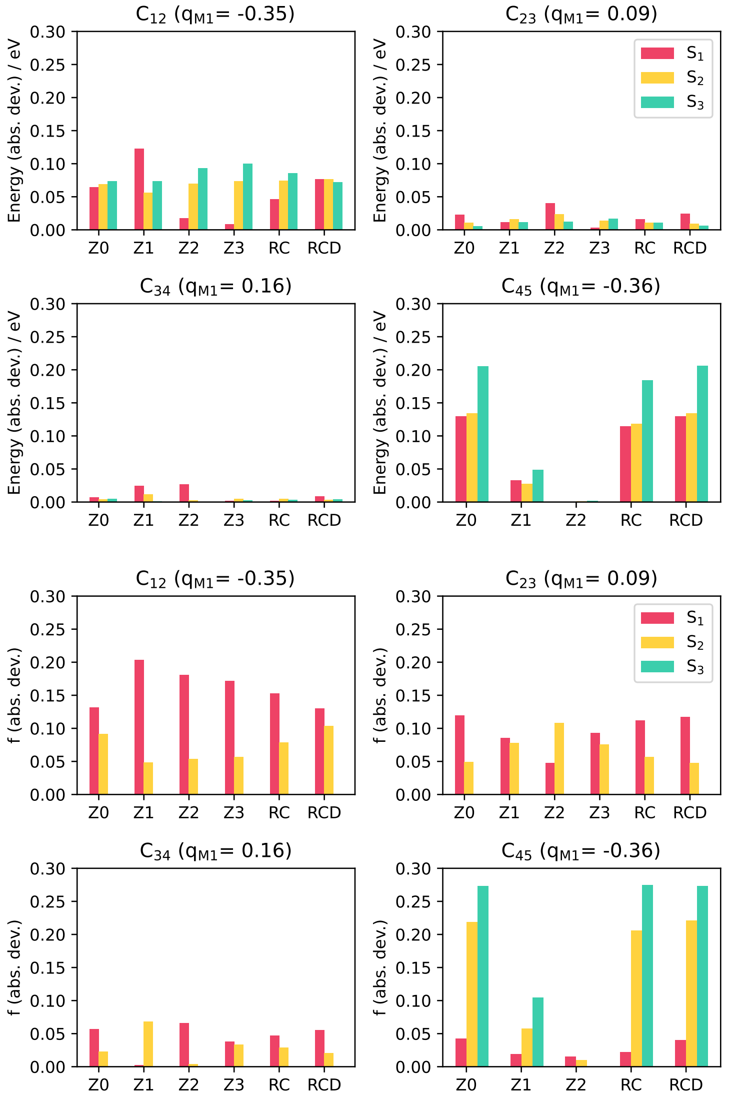

Figure S1: Top) Absolute deviation in S_1-S_3 energies (TD-PBE0/TZVP) for models C_{12}-C_{45} with the theoretical TD-PBE0/TZVP reference for each charge redistribution scheme. Bottom) Absolute deviation in S_1-S_3 oscillator strengths (PBE0/TZVP) for each truncated model compared to the full molecule embedding in RESP charges at the periodic DFT geometry and redistributed via each charge scheme.

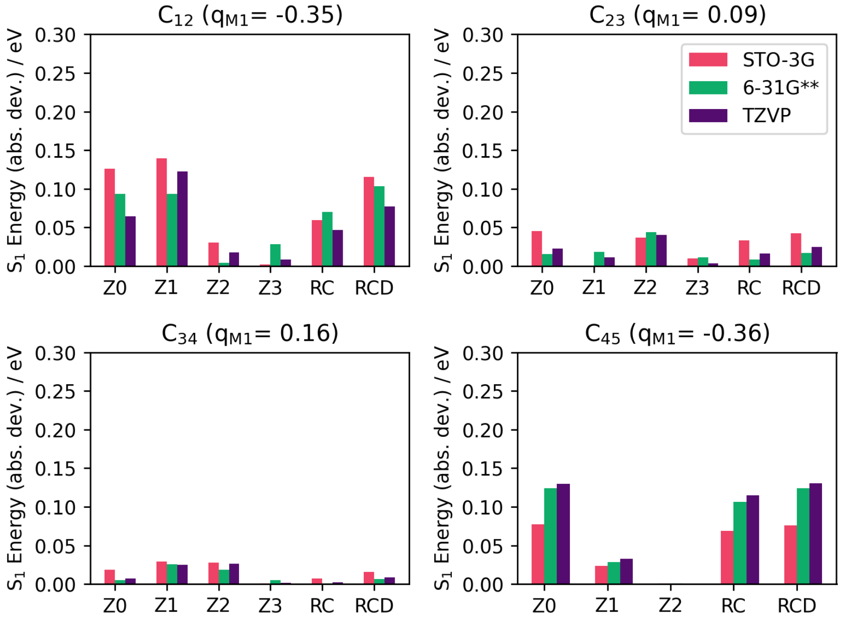

Figure S2: S_1 energy for TD-PBE0 in the STO-3G, 6-31G** and TZVP basis sets.

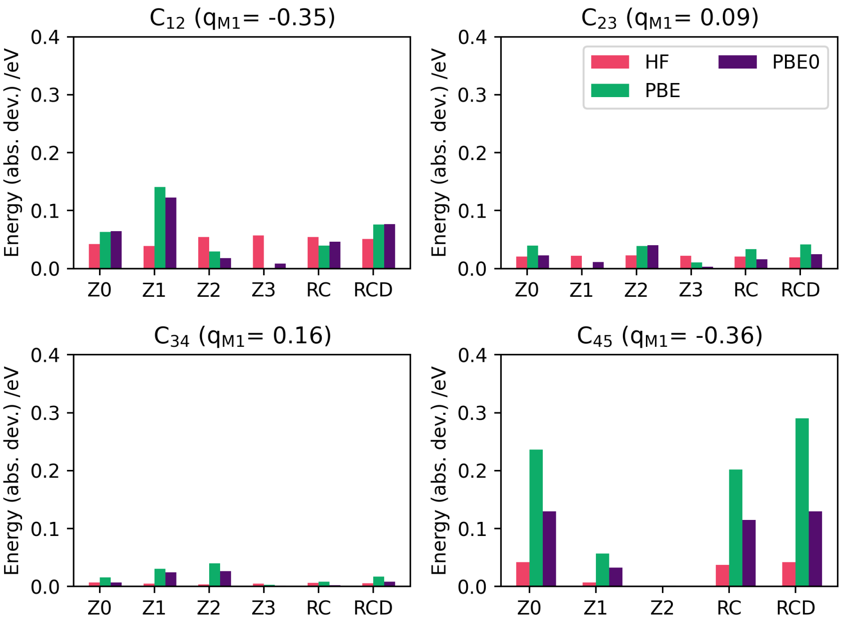

Figure S3: Deviation of the S_1 energy for TD-HF, TD-PBE, TD-PBE0 in the TZVP basis set, compared to the respective full ONIOM calculation.

3.2 Excited-state data

Table S1: diC_4-BTBT full ONIOM references.

Method	Basis set	S_1 (eV)	S_1 (f)	S_2 (eV)	S_2 (f)	S_3 (eV)	S_3 (f)
HF	STO-3G	5.637	0.73	6.421	0.12	6.862	0.00
	6-31G**	4.556	0.63	5.379	0.05	5.554	0.00
	TZVP	4.500	0.60	5.288	0.04	5.397	0.00
PBE	STO-3G	3.786	0.05	4.486	0.14	4.525	0.00
	6-31G**	3.547	0.13	3.902	0.39	3.968	0.00
	TZVP	3.469	0.12	3.826	0.01	3.844	0.34
PBE0	STO-3G	4.514	0.12	5.116	0.54	5.487	0.00
	6-31G**	4.067	0.37	4.356	0.41	4.626	0.00
	TZVP	3.998	0.33	4.298	0.40	4.482	0.00
CC(2)	def2-SV(P)	4.312	0.10	4.601	0.69	4.725	0.00

Table S2: diC$_{12}$-BTBT

Method	Basis set	Scheme	S_1 (eV)	S_1 (f)	S_2 (eV)	S_2 (f)	S_3 (eV)	S_3 (f)
HF	STO-3G	Z0	5.753	0.52	6.510	0.10	6.932	0.00
		Z1	5.685	0.48	6.330	0.15	6.819	0.00
		Z2	5.716	0.50	6.394	0.13	6.862	0.00
		Z3	5.722	0.51	6.410	0.13	6.871	0.00
		RC	5.737	0.51	6.458	0.11	6.903	0.00
		RCD	5.750	0.52	6.501	0.10	6.927	0.00
HF	6-31G**	Z0	4.628	0.41	5.420	0.04	5.602	0.00
		Z1	4.621	0.40	5.299	0.09	5.581	0.00
		Z2	4.631	0.40	5.362	0.07	5.604	0.00
		Z3	4.632	0.41	5.376	0.07	5.608	0.00
		RC	4.634	0.41	5.406	0.05	5.609	0.00
		RCD	4.634	0.41	5.429	0.04	5.605	0.00
HF	TZVP	Z0	4.542	0.41	5.302	0.04	5.431	0.00
		Z1	4.539	0.40	5.193	0.09	5.445	0.00
		Z2	4.554	0.40	5.259	0.07	5.460	0.00
		Z3	4.557	0.40	5.275	0.06	5.463	0.00
		RC	4.554	0.40	5.297	0.05	5.450	0.00
		RCD	4.551	0.40	5.312	0.03	5.433	0.00
PBE	STO-3G	Z0	3.906	0.05	4.584	0.07	4.606	0.00
		Z1	3.654	0.04	4.454	0.00	4.495	0.03
		Z2	3.760	0.04	4.532	0.00	4.543	0.04
		Z3	3.788	0.05	4.552	0.03	4.558	0.02
		RC	3.845	0.05	4.568	0.06	4.581	0.00
		RCD	3.897	0.05	4.581	0.07	4.603	0.00
PBE	6-31G**	Z0	3.634	0.08	4.015	0.24	4.056	0.00
		Z1	3.428	0.06	3.996	0.17	4.007	0.03
		Z2	3.532	0.07	4.015	0.23	4.047	0.00
		Z3	3.559	0.07	4.019	0.24	4.059	0.00
		RC	3.605	0.07	4.019	0.24	4.058	0.00
		RCD	3.645	0.08	4.018	0.23	4.056	0.00
PBE	TZVP	Z0	3.533	0.08	3.909	0.00	3.944	0.22
		Z1	3.329	0.06	3.867	0.00	3.926	0.17
		Z2	3.440	0.07	3.909	0.00	3.948	0.21
		Z3	3.469	0.07	3.923	0.00	3.954	0.22
		RC	3.509	0.07	3.915	0.00	3.950	0.22

Table S2: diC_{12}-BTBT

Method	Basis set	Scheme	S_1 (eV)	S_1 (f)	S_2 (eV)	S_2 (f)	S_3 (eV)	S_3 (f)
		RCD	3.545	0.08	3.907	0.00	3.947	0.22
PBE0	STO-3G	Z0	4.640	0.10	5.226	0.35	5.555	0.00
		Z1	4.375	0.09	5.186	0.28	5.457	0.00
		Z2	4.484	0.10	5.207	0.32	5.500	0.00
		Z3	4.512	0.10	5.212	0.33	5.510	0.00
		RC	4.574	0.10	5.219	0.34	5.534	0.00
		RCD	4.630	0.10	5.224	0.35	5.552	0.00
PBE0	6-31G**	Z0	4.160	0.21	4.442	0.30	4.696	0.00
		Z1	3.973	0.13	4.424	0.36	4.681	0.00
		Z2	4.071	0.16	4.435	0.35	4.702	0.00
		Z3	4.095	0.17	4.437	0.35	4.706	0.00
		RC	4.137	0.19	4.442	0.32	4.703	0.00
		RCD	4.170	0.22	4.448	0.29	4.694	0.00
PBE0	TZVP	Z0	4.062	0.20	4.368	0.31	4.556	0.00
		Z1	3.875	0.13	4.354	0.35	4.556	0.00
		Z2	3.980	0.15	4.368	0.34	4.575	0.00
		Z3	4.007	0.16	4.372	0.34	4.582	0.00
		RC	4.044	0.18	4.373	0.32	4.568	0.00
		RCD	4.075	0.20	4.375	0.29	4.554	0.00
CC(2)	def2-SV(P)	Z0	4.395	0.08	4.735	0.48	4.801	0.00
		Z1	4.194	0.11	4.725	0.41	4.770	0.00
		Z2	4.295	0.09	4.741	0.44	4.790	0.00
		Z3	4.320	0.09	4.745	0.45	4.796	0.00
		RC	4.367	0.08	4.743	0.46	4.802	0.00
		RCD	4.408	0.07	4.740	0.47	4.805	0.00

Table S3: diC$_{23}$-BTBT

Method	Basis set	Scheme	S$_1$ (eV)	S$_1$ (f)	S$_2$ (eV)	S$_2$ (f)	S$_3$ (eV)	S$_3$ (f)
HF	STO-3G	Z0	5.652	0.62	6.392	0.13	6.848	0.00
		Z1	5.661	0.63	6.422	0.12	6.865	0.00
		Z2	5.666	0.64	6.443	0.11	6.875	0.00
		Z3	5.660	0.63	6.417	0.12	6.863	0.00
		RC	5.655	0.63	6.401	0.13	6.854	0.00
		RCD	5.653	0.62	6.394	0.13	6.850	0.00
HF	6-31G**	Z0	4.580	0.51	5.358	0.05	5.555	0.00
		Z1	4.581	0.52	5.378	0.04	5.553	0.00
		Z2	4.582	0.52	5.393	0.04	5.550	0.00
		Z3	4.582	0.51	5.375	0.05	5.552	0.00
		RC	4.580	0.51	5.363	0.05	5.553	0.00
		RCD	4.578	0.51	5.358	0.05	5.554	0.00
HF	TZVP	Z0	4.521	0.48	5.262	0.04	5.395	0.00
		Z1	4.522	0.49	5.277	0.04	5.384	0.00
		Z2	4.522	0.49	5.290	0.03	5.377	0.00
		Z3	4.522	0.49	5.273	0.04	5.382	0.00
		RC	4.520	0.49	5.265	0.04	5.390	0.00
		RCD	4.519	0.49	5.261	0.04	5.394	0.00
PBE	STO-3G	Z0	3.741	0.05	4.486	0.07	4.497	0.01
		Z1	3.785	0.05	4.498	0.09	4.520	0.00
		Z2	3.821	0.05	4.507	0.11	4.542	0.00
		Z3	3.775	0.05	4.493	0.08	4.511	0.00
		RC	3.752	0.05	4.489	0.07	4.501	0.01
		RCD	3.743	0.05	4.487	0.07	4.498	0.01
PBE	6-31G**	Z0	3.513	0.09	3.927	0.27	3.964	0.00
		Z1	3.556	0.10	3.927	0.27	3.966	0.00
		Z2	3.592	0.11	3.928	0.27	3.971	0.00
		Z3	3.546	0.10	3.925	0.26	3.961	0.00
		RC	3.522	0.09	3.925	0.26	3.962	0.00
		RCD	3.512	0.09	3.925	0.27	3.963	0.00
PBE	TZVP	Z0	3.430	0.08	3.810	0.00	3.860	0.22
		Z1	3.470	0.09	3.811	0.00	3.861	0.23
		Z2	3.508	0.10	3.818	0.00	3.862	0.23
		Z3	3.459	0.09	3.804	0.00	3.857	0.22
		RC	3.436	0.08	3.806	0.00	3.858	0.22

Table S3: diC_{23}-BTBT

Method	Basis set	Scheme	S_1 (eV)	S_1 (f)	S_2 (eV)	S_2 (f)	S_3 (eV)	S_3 (f)
		RCD	3.428	0.08	3.809	0.00	3.857	0.22
PBE0	STO-3G	Z0	4.469	0.11	5.139	0.41	5.476	0.00
		Z1	4.515	0.11	5.141	0.43	5.490	0.00
		Z2	4.551	0.12	5.143	0.44	5.501	0.00
		Z3	4.505	0.11	5.140	0.42	5.486	0.00
		RC	4.481	0.11	5.139	0.41	5.479	0.00
		RCD	4.472	0.11	5.139	0.41	5.477	0.00
PBE0	6-31G**	Z0	4.051	0.23	4.371	0.37	4.631	0.00
		Z1	4.085	0.27	4.378	0.34	4.628	0.00
		Z2	4.110	0.31	4.385	0.31	4.627	0.00
		Z3	4.078	0.26	4.377	0.34	4.625	0.00
		RC	4.058	0.24	4.372	0.37	4.628	0.00
		RCD	4.050	0.23	4.370	0.38	4.630	0.00
PBE0	TZVP	Z0	3.975	0.21	4.309	0.35	4.477	0.00
		Z1	4.009	0.25	4.314	0.32	4.471	0.00
		Z2	4.038	0.29	4.322	0.29	4.470	0.00
		Z3	4.001	0.24	4.312	0.32	4.465	0.00
		RC	3.982	0.22	4.309	0.34	4.472	0.00
		RCD	3.973	0.22	4.307	0.35	4.476	0.00
CC(2)	def2-SV(P)	Z0	4.287	0.09	4.645	0.52	4.735	0.00
		Z1	4.327	0.09	4.643	0.54	4.737	0.00
		Z2	4.362	0.08	4.643	0.56	4.740	0.00
		Z3	4.319	0.09	4.642	0.53	4.735	0.00
		RC	4.296	0.09	4.643	0.52	4.734	0.00
		RCD	4.287	0.09	4.643	0.52	4.734	0.00

Table S4: diC_{34}-BTBT

Method	Basis set	Scheme	S_1 (eV)	S_1 (f)	S_2 (eV)	S_2 (f)	S_3 (eV)	S_3 (f)
HF	STO-3G	Z0	5.641	0.68	6.409	0.12	6.856	0.0
		Z1	5.647	0.69	6.438	0.11	6.871	0.0
		Z2	5.640	0.67	6.405	0.13	6.854	0.0
		Z3	5.644	0.68	6.421	0.12	6.862	0.0
		RC	5.643	0.68	6.416	0.12	6.860	0.0
		RCD	5.641	0.68	6.411	0.12	6.857	0.0
HF	6-31G**	Z0	4.563	0.57	5.373	0.05	5.556	0.0
		Z1	4.563	0.57	5.391	0.04	5.553	0.0
		Z2	4.562	0.57	5.366	0.05	5.555	0.0
		Z3	4.563	0.57	5.379	0.05	5.555	0.0
		RC	4.563	0.57	5.376	0.05	5.555	0.0
		RCD	4.562	0.57	5.372	0.05	5.556	0.0
HF	TZVP	Z0	4.507	0.54	5.283	0.04	5.403	0.0
		Z1	4.505	0.54	5.297	0.03	5.393	0.0
		Z2	4.503	0.55	5.275	0.04	5.402	0.0
		Z3	4.505	0.54	5.287	0.04	5.399	0.0
		RC	4.506	0.54	5.285	0.04	5.400	0.0
		RCD	4.506	0.54	5.282	0.04	5.402	0.0
PBE	STO-3G	Z0	3.767	0.05	4.487	0.11	4.516	0.0
		Z1	3.815	0.05	4.497	0.14	4.542	0.0
		Z2	3.758	0.05	4.483	0.10	4.507	0.0
		Z3	3.787	0.05	4.491	0.12	4.526	0.0
		RC	3.778	0.05	4.489	0.12	4.521	0.0
		RCD	3.770	0.05	4.488	0.11	4.517	0.0
PBE	6-31G**	Z0	3.534	0.11	3.913	0.33	3.970	0.0
		Z1	3.579	0.13	3.913	0.33	3.975	0.0
		Z2	3.517	0.11	3.910	0.33	3.964	0.0
		Z3	3.548	0.12	3.913	0.33	3.971	0.0
		RC	3.542	0.11	3.913	0.33	3.970	0.0
		RCD	3.533	0.11	3.912	0.34	3.970	0.0
PBE	TZVP	Z0	3.454	0.10	3.828	0.00	3.853	0.3
		Z1	3.500	0.12	3.833	0.01	3.854	0.3
		Z2	3.429	0.10	3.820	0.01	3.846	0.3
		Z3	3.466	0.11	3.829	0.01	3.852	0.3
		RC	3.461	0.11	3.828	0.01	3.853	0.3

Table S4: diC$_{34}$-BTBT

Method	Basis set	Scheme	S_1 (eV)	S_1 (f)	S_2 (eV)	S_2 (f)	S_3 (eV)	S_3 (f)
		RCD	3.452	0.10	3.828	0.01	3.852	0.3
PBE0	STO-3G	Z0	4.496	0.12	5.124	0.48	5.483	0.0
		Z1	4.544	0.12	5.124	0.50	5.497	0.0
		Z2	4.487	0.11	5.123	0.47	5.479	0.0
		Z3	4.515	0.12	5.125	0.49	5.488	0.0
		RC	4.507	0.12	5.124	0.48	5.486	0.0
		RCD	4.498	0.12	5.124	0.48	5.484	0.0
PBE0	6-31G**	Z0	4.061	0.30	4.361	0.39	4.630	0.0
		Z1	4.092	0.36	4.370	0.34	4.627	0.0
		Z2	4.048	0.29	4.357	0.40	4.627	0.0
		Z3	4.072	0.32	4.363	0.37	4.629	0.0
		RC	4.067	0.31	4.362	0.38	4.629	0.0
		RCD	4.060	0.30	4.360	0.39	4.630	0.0
PBE0	TZVP	Z0	3.990	0.28	4.302	0.37	4.487	0.0
		Z1	4.023	0.33	4.310	0.33	4.484	0.0
		Z2	3.971	0.27	4.295	0.39	4.483	0.0
		Z3	4.000	0.30	4.303	0.36	4.485	0.0
		RC	3.996	0.29	4.303	0.37	4.485	0.0
		RCD	3.989	0.28	4.301	0.38	4.487	0.0
CC(2)	def2-SV(P)	Z0	4.302	0.10	4.617	0.61	4.730	0.0
		Z1	4.344	0.09	4.614	0.63	4.733	0.0
		Z2	4.285	0.10	4.614	0.61	4.727	0.0
		Z3	4.315	0.09	4.615	0.62	4.730	0.0
		RC	4.309	0.09	4.616	0.62	4.730	0.0
		RCD	4.301	0.10	4.616	0.61	4.730	0.0

Table S5: diC_{45}-BTBT

Method	Basis set	Scheme	S_1 (eV)	S_1 (f)	S_2 (eV)	S_2 (f)	S_3 (eV)	S_3 (f)
		Z0	5.602	0.73	6.407	0.12	6.849	0.00
		Z1	5.630	0.71	6.413	0.12	6.857	0.00
HF	STO-3G	Z2	5.639	0.71	6.421	0.12	6.862	0.00
		RC	5.606	0.73	6.407	0.12	6.850	0.00
		RCD	5.602	0.73	6.407	0.12	6.850	0.00
		Z0	4.515	0.64	5.357	0.04	5.522	0.00
		Z1	4.549	0.61	5.371	0.05	5.547	0.00
HF	6-31G**	Z2	4.557	0.61	5.379	0.05	5.554	0.00
		RC	4.520	0.63	5.358	0.04	5.525	0.00
		RCD	4.515	0.64	5.356	0.04	5.521	0.00
		Z0	4.458	0.61	5.256	0.03	5.363	0.01
		Z1	4.492	0.58	5.279	0.04	5.390	0.00
HF	TZVP	Z2	4.501	0.58	5.287	0.04	5.398	0.00
		RC	4.462	0.61	5.258	0.03	5.366	0.01
		RCD	4.457	0.61	5.256	0.03	5.362	0.01
		Z0	3.663	0.05	4.205	0.11	4.379	0.37
		Z1	3.752	0.05	4.399	0.05	4.566	0.17
PBE	STO-3G	Z2	3.785	0.05	4.488	0.14	4.525	0.00
		RC	3.678	0.05	4.250	0.05	4.488	0.52
		RCD	3.666	0.05	4.219	0.08	4.415	0.46
		Z0	3.300	0.23	3.504	0.07	3.621	0.03
		Z1	3.498	0.12	3.826	0.14	3.943	0.36
PBE	6-31G**	Z2	3.546	0.13	3.906	0.37	3.970	0.00
		RC	3.372	0.16	3.636	0.08	3.745	0.38
		RCD	3.313	0.22	3.527	0.09	3.630	0.04
		Z0	3.233	0.17	3.278	0.01	3.468	0.11
		Z1	3.412	0.11	3.724	0.03	3.851	0.45
PBE	TZVP	Z2	3.468	0.12	3.828	0.01	3.847	0.33
		RC	3.267	0.08	3.290	0.05	3.545	0.02
		RCD	3.179	0.01	3.245	0.16	3.486	0.09
		Z0	4.437	0.13	4.978	0.46	5.317	0.34
		Z1	4.491	0.12	5.091	0.49	5.450	0.13
PBE0	STO-3G	Z2	4.514	0.12	5.118	0.52	5.487	0.00

Table S5: diC$_{45}$-BTBT

Method	Basis set	Scheme	S_1 (eV)	S_1 (f)	S_2 (eV)	S_2 (f)	S_3 (eV)	S_3 (f)
		RC	4.445	0.13	5.001	0.45	5.338	0.33
		RCD	4.438	0.13	4.983	0.46	5.321	0.34
		Z0	3.943	0.43	4.236	0.26	4.420	0.16
		Z1	4.038	0.35	4.334	0.38	4.579	0.06
PBE0	6-31G**	Z2	4.067	0.35	4.358	0.40	4.627	0.00
		RC	3.960	0.40	4.253	0.28	4.443	0.16
		RCD	3.943	0.42	4.237	0.26	4.420	0.16
		Z0	3.868	0.38	4.164	0.18	4.277	0.27
		Z1	3.965	0.32	4.271	0.34	4.434	0.10
PBE0	TZVP	Z2	3.997	0.32	4.299	0.39	4.484	0.00
		RC	3.883	0.36	4.179	0.19	4.298	0.28
		RCD	3.868	0.37	4.164	0.18	4.276	0.27
		Z0	4.261	0.15	4.462	0.53	4.667	0.15
		Z1	4.294	0.10	4.574	0.61	4.717	0.05
CC(2)	def2-SV(P)	Z2	4.312	0.10	4.605	0.67	4.727	0.00
		RC	4.265	0.14	4.477	0.53	4.675	0.15
		RCD	4.261	0.15	4.462	0.53	4.667	0.15

4 Polythiophene embedded cluster calculations

4.1 1-D model

This was true for both ESP and Mulliken charges, for both the S_0- and S_1-electron densities. For example, compared to the real-high reference (10 thiophene units), the 1-unit model overestimates the first five singlet excited states by nearly 3.3 eV across all embedding schemes. In the largest possible model containing 8 units, this error is reduced to ≈ 0.22 eV. Nevertheless, the 8-unit model contains only 10 fewer atoms than the real-high calculation, meaning the savings in computational cost are very limited. Clearly, cutting at an sp^2-hybridised C=C bond is not a fruitful approach due to the significance of the extended π-system in polythiophene.[3] This has important implications for studies on semiconducting MOFs, if transport occurs through electronic band conduction.[14]

Clearly, the perturbation by PCE is very small, with the embedded cluster models generally reproducing the vertical excitations calculated in vacuum. The small difference in behaviour between the S_0- and S_1-Mulliken embedded cluster models is reflected in the relatively small values of the M_1 charges, which range from -0.11 to -0.12 e. Interestingly, despite having a smaller average M_1 charge of 0.06 e, the electrostatic effect is larger in the ESP-embedded clusters containing one and two thiophene units. This arises from slightly larger charges on the neighbouring M_2 and M_3 atoms, which are nearly neutral in the Mulliken populations but range from -0.21 to 0.22 e in the ESP analysis. Of course, the sulphur (M_2) atoms have larger charges in the Mulliken analysis ($\sim 0.14\,e$) than in the ESP analysis ($\sim -0.10\,e$). However, the large C–S bond length ($\sim 1.748\,\text{Å}$) compared to C=C ($\sim 1.397\,\text{Å}$) places these M_2 charges farther from the QM wavefunction, thereby reducing their capacity to polarise. Moreover, this explains why the Z2 and Z3 schemes are often required, beyond Z1, despite the large interaction distance. The modest difference in the S_0 and S_1 charges arises because both ground- and excited-state ωB97X-D/cc-pVDZ calculations were performed at the Franck-Condon geometry, a measure taken to decouple nuclear and electronic effects. Besides, the real-high reference itself is too small to faithfully reproduce the true excited-state bands of the polythiophene crystal, particularly as it includes only intramolecular effects. Specifically, the 10-unit model places a bright S_1 state at 2.34 eV, slightly higher than the experimental optical gap previously reported at ≈ 2 eV.[27] Previous DFT studies have obtained the optical gap of the infinite polythiophene chain by linear extrapolation of 1-D models up to 30 thiophene units. However, this approach also neglects the mechanical and electrostatic coupling to the environment.[28]

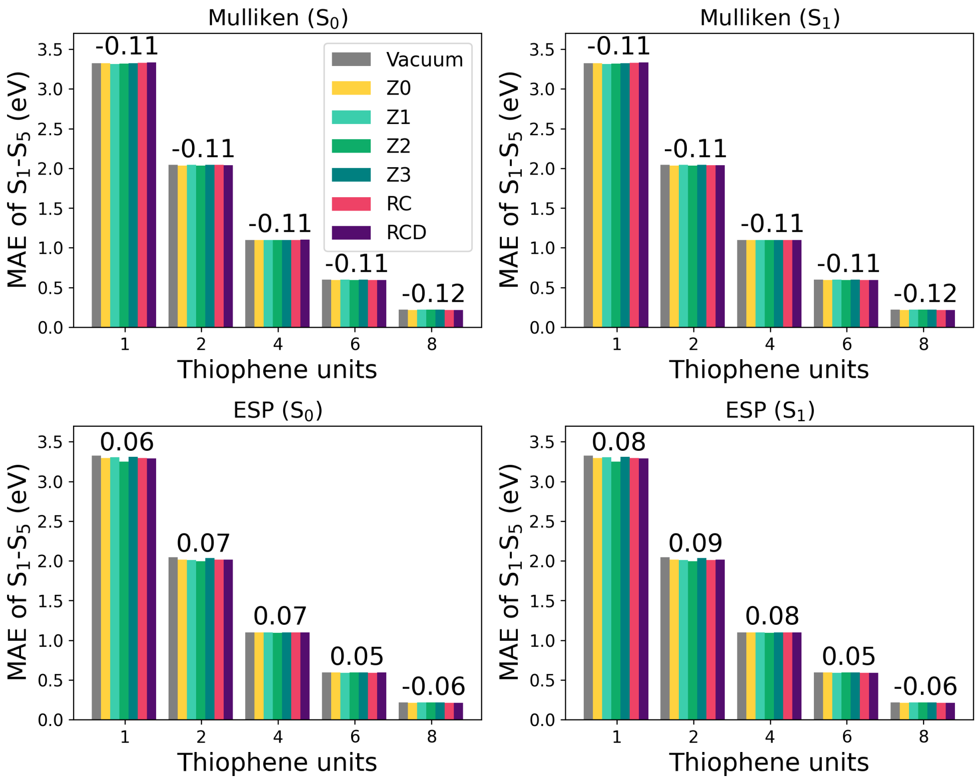

Figure S4: MAE of the TD-ωB97X-D/cc-pVDZ (bars) energy (S_1-S_5) with respect to the 10-unit real-high calculation is shown for each polythiophene model and charge redistribution scheme, embedded in ESP charges and Mulliken charges obtained from both the S_0 and S_1 charge densities. The average M_1 charge (in e) is shown above the bar for each model.

Table S6: TD-ωB97X-D/cc-pVDZ calculations for polythiophene with electronic embedding in Mulliken-S_0 charges.

Thiophene units	Scheme	S_1 (eV)	S_1 (f)	S_2 (eV)	S_2 (f)	S_3 (eV)	S_3 (f)
10 (real-high)	Vac.	2.342	4.07	2.799	0.00	3.242	0.28
1	Vac.	5.673	0.10	5.888	0.08	6.566	0.00
	Z0	5.655	0.10	5.939	0.08	6.485	0.00
	Z1	5.683	0.10	5.867	0.09	6.576	0.00
	Z2	5.666	0.10	5.898	0.08	6.525	0.00
	Z3	5.671	0.10	5.890	0.08	6.560	0.00
	RC	5.670	0.10	5.913	0.08	6.540	0.00
	RCD	5.663	0.10	5.938	0.08	6.505	0.00
2	Vac.	3.985	0.44	5.240	0.00	5.438	0.10
	Z0	3.974	0.44	5.272	0.00	5.470	0.10
	Z1	3.993	0.45	5.226	0.00	5.422	0.10
	Z2	3.980	0.44	5.246	0.00	5.445	0.10

Table S6: TD-ωB97X-D/cc-pVDZ calculations for polythiophene with electronic embedding in Mulliken-S_0 charges.

Thiophene units	Scheme	S_1 (eV)	S_1 (f)	S_2 (eV)	S_2 (f)	S_3 (eV)	S_3 (f)
	Z3	3.984	0.44	5.241	0.00	5.439	0.10
	RC	3.982	0.44	5.255	0.00	5.453	0.10
	RCD	3.978	0.44	5.270	0.00	5.469	0.10
4	Vac.	2.974	1.28	4.029	0.00	4.626	0.00
	Z0	2.968	1.28	4.025	0.00	4.616	0.00
	Z1	2.977	1.29	4.030	0.00	4.636	0.00
	Z2	2.972	1.28	4.027	0.00	4.622	0.00
	Z3	2.973	1.28	4.029	0.00	4.625	0.00
	RC	2.972	1.28	4.029	0.00	4.622	0.00
	RCD	2.970	1.28	4.027	0.00	4.617	0.00
6	Vac.	2.616	2.20	3.372	0.00	4.035	0.13
	Z0	2.612	2.20	3.368	0.00	4.033	0.14
	Z1	2.618	2.20	3.374	0.00	4.036	0.13
	Z2	2.615	2.20	3.371	0.00	4.034	0.13
	Z3	2.615	2.20	3.372	0.00	4.035	0.13
	RC	2.615	2.20	3.371	0.00	4.035	0.13
	RCD	2.613	2.19	3.369	0.00	4.034	0.13
8	Vac.	2.441	3.13	3.019	0.00	3.550	0.20
	Z0	2.438	3.13	3.014	0.00	3.546	0.20
	Z1	2.442	3.13	3.020	0.00	3.551	0.20
	Z2	2.441	3.13	3.018	0.00	3.549	0.20
	Z3	2.441	3.13	3.019	0.00	3.550	0.20
	RC	2.440	3.13	3.017	0.00	3.549	0.20
	RCD	2.438	3.12	3.015	0.00	3.547	0.20

Table S7: TD-ωB97X-D/cc-pVDZ calculations for polythiophene with electronic embedding in Mulliken-S_1 charges.

Thiophene units	Scheme	S_1 (eV)	S_1 (f)	S_2 (eV)	S_2 (f)	S_3 (eV)	S_3 (f)
10 (real-high)	Vac.	2.342	4.07	2.799	0.00	3.242	0.28
1	Vac.	5.673	0.10	5.888	0.08	6.566	0.00
	Z0	5.653	0.10	5.939	0.08	6.477	0.00
	Z1	5.681	0.10	5.869	0.09	6.568	0.00
	Z2	5.666	0.10	5.898	0.08	6.523	0.00
	Z3	5.672	0.10	5.890	0.08	6.560	0.00
	RC	5.669	0.10	5.914	0.08	6.532	0.00
	RCD	5.662	0.10	5.938	0.08	6.498	0.00
2	Vac.	3.985	0.44	5.240	0.00	5.438	0.10
	Z0	3.973	0.44	5.272	0.00	5.471	0.10
	Z1	3.992	0.45	5.227	0.00	5.423	0.10
	Z2	3.980	0.44	5.246	0.00	5.445	0.10
	Z3	3.984	0.44	5.241	0.00	5.439	0.10
	RC	3.982	0.44	5.255	0.00	5.454	0.10
	RCD	3.977	0.44	5.271	0.00	5.470	0.10
4	Vac.	2.974	1.28	4.029	0.00	4.626	0.00
	Z0	2.968	1.28	4.025	0.00	4.615	0.00
	Z1	2.977	1.29	4.030	0.00	4.636	0.00
	Z2	2.972	1.28	4.027	0.00	4.622	0.00
	Z3	2.973	1.28	4.029	0.00	4.625	0.00
	RC	2.972	1.28	4.028	0.00	4.622	0.00
	RCD	2.970	1.28	4.027	0.00	4.617	0.00
6	Vac.	2.616	2.20	3.372	0.00	4.035	0.13
	Z0	2.612	2.20	3.368	0.00	4.032	0.14
	Z1	2.618	2.20	3.374	0.00	4.036	0.13
	Z2	2.615	2.20	3.371	0.00	4.034	0.13
	Z3	2.615	2.20	3.372	0.00	4.035	0.13
	RC	2.615	2.20	3.371	0.00	4.035	0.13
	RCD	2.613	2.19	3.369	0.00	4.034	0.13
8	Vac.	2.441	3.13	3.019	0.00	3.550	0.20
	Z0	2.438	3.13	3.014	0.00	3.546	0.20
	Z1	2.442	3.13	3.020	0.00	3.551	0.20
	Z2	2.441	3.13	3.018	0.00	3.549	0.20

Table S7: TD-ωB97X-D/cc-pVDZ calculations for polythiophene with electronic embedding in Mulliken-S_1 charges.

Thiophene units	Scheme	S_1 (eV)	S_1 (f)	S_2 (eV)	S_2 (f)	S_3 (eV)	S_3 (f)
	Z3	2.441	3.13	3.019	0.00	3.550	0.20
	RC	2.440	3.13	3.017	0.00	3.549	0.20
	RCD	2.438	3.12	3.015	0.00	3.547	0.20

Table S8: TD-ωB97X-D/cc-pVDZ calculations for polythiophene with electronic embedding in ESP-S_0 charges.

Thiophene units	Scheme	S_1 (eV)	S_1 (f)	S_2 (eV)	S_2 (f)	S_3 (eV)	S_3 (f)
10 (real-high)	Vac.	2.342	4.07	2.799	0.00	3.242	0.28
1	Vac.	5.673	0.10	5.888	0.08	6.566	0.00
	Z0	5.661	0.10	5.910	0.08	6.419	0.00
	Z1	5.642	0.10	5.958	0.08	6.356	0.00
	Z2	5.652	0.10	5.895	0.09	6.317	0.00
	Z3	5.671	0.10	5.880	0.09	6.538	0.00
	RC	5.651	0.10	5.928	0.08	6.382	0.00
	RCD	5.655	0.10	5.912	0.08	6.406	0.00
2	Vac.	3.985	0.44	5.240	0.00	5.438	0.10
	Z0	3.977	0.44	5.253	0.00	5.452	0.10
	Z1	3.964	0.44	5.284	0.00	5.485	0.10
	Z2	3.978	0.44	5.243	0.00	5.441	0.10
	Z3	3.986	0.45	5.234	0.00	5.432	0.10
	RC	3.971	0.44	5.264	0.00	5.465	0.10
	RCD	3.975	0.44	5.254	0.00	5.454	0.10
4	Vac.	2.974	1.28	4.029	0.00	4.626	0.00
	Z0	2.972	1.28	4.026	0.00	4.620	0.00
	Z1	2.964	1.27	4.020	0.00	4.606	0.00
	Z2	2.974	1.29	4.022	0.00	4.632	0.00
	Z3	2.975	1.28	4.027	0.00	4.630	0.00
	RC	2.969	1.28	4.023	0.00	4.615	0.00
	RCD	2.971	1.28	4.024	0.00	4.619	0.00
6	Vac.	2.616	2.20	3.372	0.00	4.035	0.13
	Z0	2.614	2.19	3.369	0.00	4.032	0.13
	Z1	2.610	2.19	3.365	0.00	4.029	0.13
	Z2	2.617	2.20	3.370	0.00	4.031	0.13
	Z3	2.616	2.20	3.372	0.00	4.035	0.13
	RC	2.612	2.19	3.367	0.00	4.031	0.13
	RCD	2.613	2.20	3.368	0.00	4.032	0.13
8	Vac.	2.441	3.13	3.019	0.00	3.550	0.20
	Z0	2.437	3.13	3.012	0.00	3.543	0.20
	Z1	2.439	3.13	3.015	0.00	3.547	0.20
	Z2	2.440	3.13	3.017	0.00	3.547	0.20

Table S8: TD-ωB97X-D/cc-pVDZ calculations for polythiophene with electronic embedding in ESP-S_0 charges.

Thiophene units	Scheme	S_1 (eV)	S_1 (f)	S_2 (eV)	S_2 (f)	S_3 (eV)	S_3 (f)
	Z3	2.441	3.13	3.018	0.00	3.550	0.20
	RC	2.438	3.13	3.014	0.00	3.545	0.20
	RCD	2.437	3.13	3.012	0.00	3.544	0.20

Table S9: TD-ωB97X-D/cc-pVDZ calculations for polythiophene with electronic embedding in ESP-S_1 charges.

Thiophene units	Scheme	S_1 (eV)	S_1 (f)	S_2 (eV)	S_2 (f)	S_3 (eV)	S_3 (f)
10 (real-high)	Vac.	2.342	4.07	2.799	0.00	3.242	0.28
1	Vac.	5.673	0.10	5.888	0.08	6.566	0.00
	Z0	5.662	0.10	5.906	0.08	6.421	0.00
	Z1	5.639	0.10	5.963	0.08	6.346	0.00
	Z2	5.653	0.10	5.893	0.09	6.317	0.00
	Z3	5.671	0.10	5.880	0.09	6.539	0.00
	RC	5.649	0.10	5.927	0.08	6.378	0.00
	RCD	5.655	0.10	5.907	0.08	6.407	0.00
2	Vac.	3.985	0.44	5.240	0.00	5.438	0.10
	Z0	3.978	0.44	5.250	0.00	5.450	0.10
	Z1	3.962	0.44	5.286	0.00	5.489	0.10
	Z2	3.979	0.44	5.242	0.00	5.439	0.10
	Z3	3.986	0.45	5.234	0.00	5.432	0.10
	RC	3.971	0.44	5.264	0.00	5.465	0.10
	RCD	3.974	0.44	5.252	0.00	5.452	0.10
4	Vac.	2.974	1.28	4.029	0.00	4.626	0.00
	Z0	2.972	1.28	4.026	0.00	4.620	0.00
	Z1	2.963	1.27	4.020	0.00	4.604	0.00
	Z2	2.975	1.29	4.022	0.00	4.632	0.00
	Z3	2.975	1.28	4.027	0.00	4.630	0.00
	RC	2.969	1.28	4.023	0.00	4.615	0.00
	RCD	2.970	1.28	4.024	0.00	4.619	0.00
6	Vac.	2.616	2.20	3.372	0.00	4.035	0.13
	Z0	2.613	2.19	3.369	0.00	4.032	0.13
	Z1	2.610	2.19	3.364	0.00	4.029	0.13
	Z2	2.617	2.20	3.370	0.00	4.030	0.13
	Z3	2.616	2.20	3.372	0.00	4.035	0.13
	RC	2.612	2.19	3.367	0.00	4.031	0.13
	RCD	2.613	2.20	3.368	0.00	4.032	0.13
8	Vac.	2.441	3.13	3.019	0.00	3.550	0.20
	Z0	2.437	3.13	3.011	0.00	3.543	0.20
	Z1	2.439	3.13	3.015	0.00	3.547	0.20
	Z2	2.440	3.13	3.017	0.00	3.548	0.20

Table S9: TD-ωB97X-D/cc-pVDZ calculations for polythiophene with electronic embedding in ESP-S_1 charges.

Thiophene units	Scheme	S_1 (eV)	S_1 (f)	S_2 (eV)	S_2 (f)	S_3 (eV)	S_3 (f)
	Z3	2.441	3.13	3.018	0.00	3.550	0.20
	RC	2.438	3.13	3.013	0.00	3.545	0.20
	RCD	2.437	3.13	3.012	0.00	3.544	0.20

Table S10: TD-ωB97X-D/cc-pVDZ calculations for polythiophene with electronic embedding in Z2 ESP charges at the periodic DFT geometry.

Thiophene chains	Thiophene units	S_1 (eV)	S_1 (f)	S_2 (eV)	S_2 (f)	S_3 (eV)	S_3 (f)	S_4 (eV)	S_4 (f)
4 (real-high)	9	2.221	0.00	2.224	0.01	2.283	0.00	2.561	12.08
	1	5.651	0.10	5.902	0.09	6.334	0.00	6.679	0.00
	2	3.980	0.45	5.219	0.01	5.445	0.10	5.557	0.00
	3	3.324	0.84	4.611	0.01	5.056	0.00	5.162	0.00
1	5	2.748	1.74	3.639	0.00	4.321	0.02	4.385	0.08
	7	2.516	2.67	3.175	0.00	3.765	0.16	4.046	0.00
	1	5.638	0.00	5.670	0.17	5.818	0.15	5.883	0.05
	2	3.831	0.02	4.024	0.73	4.588	0.01	5.027	0.00
	3	3.111	0.00	3.413	1.43	3.858	0.02	4.279	0.00
2	5	2.586	0.00	2.843	3.12	3.385	0.04	3.478	0.01
	7	2.357	0.00	2.605	4.89	2.946	0.00	3.253	0.04
	1	5.537	0.09	5.623	0.00	5.650	0.13	5.789	0.21
	2	3.805	0.02	3.840	0.01	4.062	0.96	4.431	0.00
	3	3.097	0.00	3.161	0.01	3.455	1.88	3.647	0.00
3	5	2.547	0.00	2.577	0.00	2.914	4.19	3.300	0.00
	7	2.336	0.00	2.354	0.00	2.664	6.77	2.915	0.00
	1	5.520	0.03	5.557	0.17	5.610	0.02	5.641	0.04
	2	3.787	0.02	3.802	0.01	3.896	0.02	4.088	1.15
	3	3.073	0.00	3.131	0.02	3.228	0.01	3.483	2.30
4	5	2.522	0.01	2.534	0.00	2.629	0.00	2.962	5.18
	7	2.319	0.00	2.326	0.01	2.401	0.00	2.707	8.55

Table S11: TD-ωB97X-D/cc-pVDZ calculations for polythiophene in vacuum at the periodic DFT geometry.

Thiophene chains	Thiophene units	S_1 (eV)	S_1 (f)	S_2 (eV)	S_2 (f)	S_3 (eV)	S_3 (f)	S_4 (eV)	S_4 (f)
4 (real-high)	9	2.221	0.00	2.224	0.01	2.283	0.00	2.561	12.08
1	1	5.673	0.10	5.888	0.08	6.566	0.00	6.847	0.00
	2	3.985	0.44	5.240	0.00	5.438	0.10	5.709	0.00
	3	3.324	0.84	4.602	0.01	5.076	0.00	5.152	0.00
	5	2.759	1.74	3.643	0.00	4.334	0.00	4.395	0.09
	7	2.515	2.66	3.172	0.00	3.762	0.17	4.043	0.00
2	1	5.659	0.01	5.693	0.17	5.811	0.15	5.874	0.03
	2	3.851	0.02	4.039	0.73	4.598	0.01	5.059	0.00
	3	3.126	0.00	3.426	1.42	3.807	0.03	4.369	0.00
	5	2.588	0.01	2.845	3.08	3.239	0.06	3.476	0.01
	7	2.354	0.01	2.602	4.82	2.943	0.00	2.999	0.10
3	1	5.553	0.09	5.645	0.00	5.677	0.13	5.773	0.28
	2	3.821	0.02	3.853	0.00	4.074	0.97	4.582	0.00
	3	3.108	0.00	3.171	0.01	3.461	1.89	3.852	0.00
	5	2.551	0.00	2.586	0.00	2.920	4.20	3.220	0.01
	7	2.337	0.00	2.358	0.00	2.665	6.79	2.917	0.00
4	1	5.543	0.01	5.568	0.18	5.640	0.01	5.654	0.07
	2	3.804	0.02	3.807	0.01	3.903	0.01	4.095	1.18
	3	3.080	0.00	3.137	0.02	3.231	0.00	3.486	2.32
	5	2.530	0.01	2.544	0.00	2.636	0.00	2.969	5.16
	7	2.321	0.00	2.328	0.01	2.403	0.00	2.709	8.57

5 QMOF-d29cec2 embedded cluster calculations

Initially, we analyse the Ewald charge distribution close to the QM:QM' which, as was also shown for diC$_4$-BTBT, determines the extent of overpolarisation. Table S12 contains important charges from the periodic population analyses, that were used to generate the Ewald embedding. In the table, 'node' that indicates the point charge lies within the node, as the carbon atom (M$_1$) is directly bonded to the Zn-oxo group. Accordingly, 'linker' refers to the carbon atom (M$_1$) lying within the linker, and is directly bonded to the 'node' carbon M$_1$ (See Figure 7 in main text). The M$_1$ atoms obtained from the Mulliken population analysis are smaller than the RESP and REPEAT analysis for both the linker and node. The linker M$_1$ charges are much larger, ranging from 0.13 to 0.28 for the Mulliken analysis, and 0.44 to 0.72 e for the ESP-based analysis, at which point overpolarisation is anticipated. This is interesting considering these M$_1$ atoms are bonded to electronegative oxygen atoms, and is likely due to the Zn atoms compensating for additional electron population. For instance, the charges on the Zn atoms of the node are very large, ranging from 1.27-1.43 e in the ESP-based Ewald embedding and 0.55 to 0.56 e in the Mulliken. Consequently, the M$_1$ carbons within the organic linkers are close to neutral, ranging from -0.03 to 0.09 e in the Mulliken analysis, and -0.08 to 0.24 in the ESP-based analyses. The slighter larger charges arise in the larger aromatic linker. Interestingly, the difference between PBE and PBE0 is not large for all analyses, lying in the range of 0.01-0.10 e. This indicates that, similar to geometry optimisations, a ground-state population is likely suitable to generate embedding. This is important for MOFs where hybrid DFT can be prohibitively expensive.[14] Overall, these results suggest the C-C bond is nearly neutral and therefore a good place to make the QM/QM' cut rather than neighbouring dative M-L bond, which requires more sophisticated tuning with pseudopontentials.[29]

Identity	Location	Type	Mulliken (PBE)	Mulliken (PBE0)	RESP (PBE)	RESP (PBE0)	REPEAT (PBE)	REPEAT (PBE0)
Zn	Node	M_3	0.55	0.66	1.33	1.37	1.27	1.32
Zn			0.55	0.66	1.30	1.33	1.26	1.31
Zn			0.54	0.65	1.35	1.38	1.26	1.31
Zn			0.55	0.65	1.39	1.43	1.29	1.34
C	Node	M_1	0.13	0.20	0.65	0.71	0.60	0.67
C			0.21	0.28	0.48	0.52	0.48	0.54
C			0.12	0.18	0.66	0.72	0.59	0.66
C			0.12	0.19	0.63	0.69	0.58	0.66
C			0.21	0.28	0.50	0.54	0.44	0.50
C			0.12	0.19	0.70	0.76	0.57	0.63
C	Linker	M_1	0.09	0.09	0.13	0.11	-0.05	-0.08
C			0.05	0.04	0.01	-0.02	-0.03	-0.07
C			-0.03	-0.03	0.15	0.14	0.21	0.20
C			0.06	0.06	0.05	0.02	-0.03	-0.06
C			-0.03	-0.03	0.11	0.10	0.24	0.23
C			0.05	0.05	0.12	0.10	0.05	0.03

Table S12: Charges obtained from Mulliken, RESP, and REPEAT population analyses from a periodic GPW with CP2K at the PBE/DZVP-MOLOPT-SR and PBE0/DZVP-MOLOPT-SR/admm-dzvp levels of theory. The M$_1$ charge is the most important charge to be redistributed. Only the PBE0 charges are used to generate Ewald-embedded clusters.

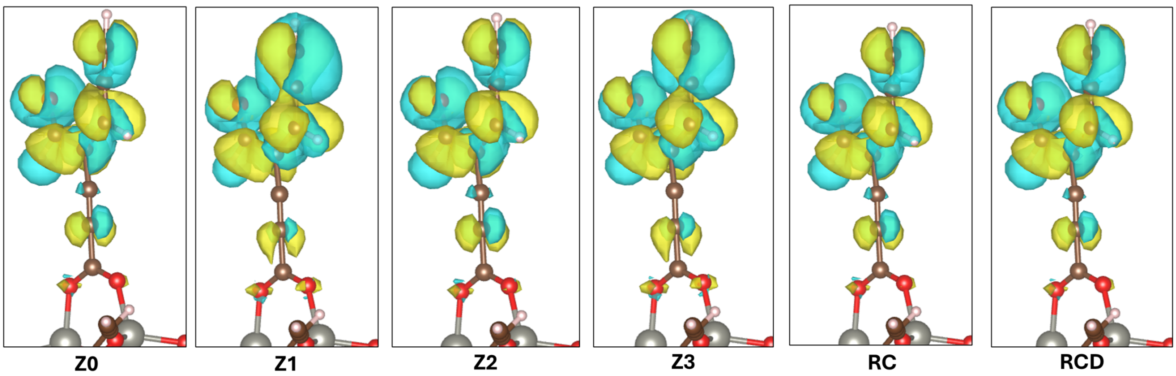

Figure S5: S_1-S_0 density differences for RESP/Ewald embedded clusters (TD-ωB97X-D/TZVP).

Table S13: Embedded cluster model and cluster model calculations on QMOF-d29cec2. All TDDFT calculations were carried out in the TZVP basis set and with the ωB97X-D functional.

Embedding	Method	Scheme	S_1 (eV)	S_1 (f)	S_2 (eV)	S_2 (f)	S_3 (eV)	S_3 (f)
RESP	TDDFT	Z0	2.98	0.00	3.15	0.10	3.52	0.10
		Z1	2.91	0.00	3.00	0.24	3.36	0.05
		Z2	2.94	0.00	3.17	0.05	3.58	0.05
		Z3	2.88	0.00	3.00	0.24	3.31	0.04
		RC	2.99	0.00	3.14	0.13	3.51	0.12
		RCD	2.97	0.00	3.15	0.10	3.52	0.11
REPEAT	TDDFT	Z0	2.98	0.00	3.17	0.08	3.56	0.08
		Z1	2.94	0.00	3.02	0.23	3.38	0.06
		Z2	2.94	0.00	3.18	0.05	3.59	0.04
		Z3	2.88	0.00	3.01	0.23	3.32	0.05
		RC	2.99	0.00	3.16	0.10	3.54	0.10
		RCD	2.96	0.00	3.17	0.08	3.55	0.08
Mulliken	TDDFT	Z0	2.98	0.00	3.15	0.09	3.53	0.09
		Z1	2.96	0.00	3.09	0.18	3.45	0.11
		Z2	2.96	0.00	3.18	0.06	3.58	0.06
		Z3	2.94	0.00	3.12	0.15	3.48	0.12
		RC	2.97	0.00	3.15	0.11	3.52	0.10
		RCD	2.97	0.00	3.15	0.10	3.53	0.09

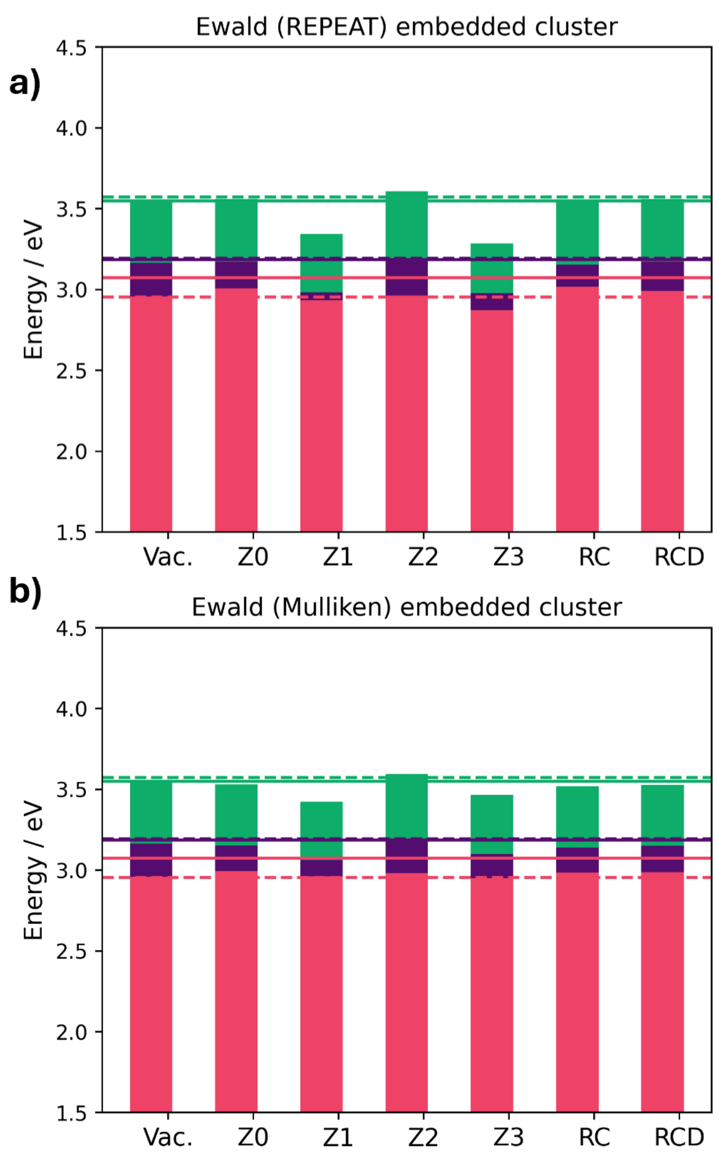

Figure S6: TD-ωB97X-D/TZVP calculations of QMOF-d29cec2 embedded with Ewald charges, using REPEAT and Mulliken charges on unit cell. The Ewald charges for the embedded cluster were redistributed according to the Z0-Z3, RC and RCD schemes. The periodic reference at the TD-ωB97X-D level is shown.

6 MOF-5 cluster and embedded cluster calculations

6.1 Discussion on choice of functional

The choice of exchange-correlation functional influences the position of the spectrum, but only slightly affects the lineshape. The absorption peak maximum increases in energy in the order ωB97X-D, CAM-B3LYP, PBE0, B3LYP. In this ordering, both ωB97X-D, CAM-B3LYP for the MOF-5 Fragment models match experiment better than H_2BDC, than in the global hybrids. The long-range correction results in proper treatment of the long-range exchange through a range-separation parameter, which corrects the long-range asymptotic behaviour of the functional.[30] This is important in MOFs, where LLCT, MLCT, and LMCT transitions are photochemically relevant and susceptible to incorrect predictions by global hybrids. In practice, range-separated hybrids predict higher energy excitations than global hybrids, particularly for CT states which are significantly overstabilised in the latter. As all the bright excitations are localised on the linker with know CT character, a red-shift in energy is therefore the key difference in the observed spectra for each class of functional. In terms of the excited-states, the bright state in Fragment B of the intense excitation is much larger at $S_1$0 with many more low-lying excitations becoming accessible, but adding negligible broadening to this region of the spectrum as the states are dark. Notably, visualisation of S_4 for the TD-B3LYP shows LLCT character ($f = 0.0009$), where the low change in transition dipole moment suggesting this does not contribute significantly to the absorption. This will be important when investigating minima of the singlet manifold in the emission properties of MOF-5. In regard to absorption, vibrationally-broadened spectra calculations were performed for CAM-B3LYP only, due to much larger computational cost in calculating 200 configurations.

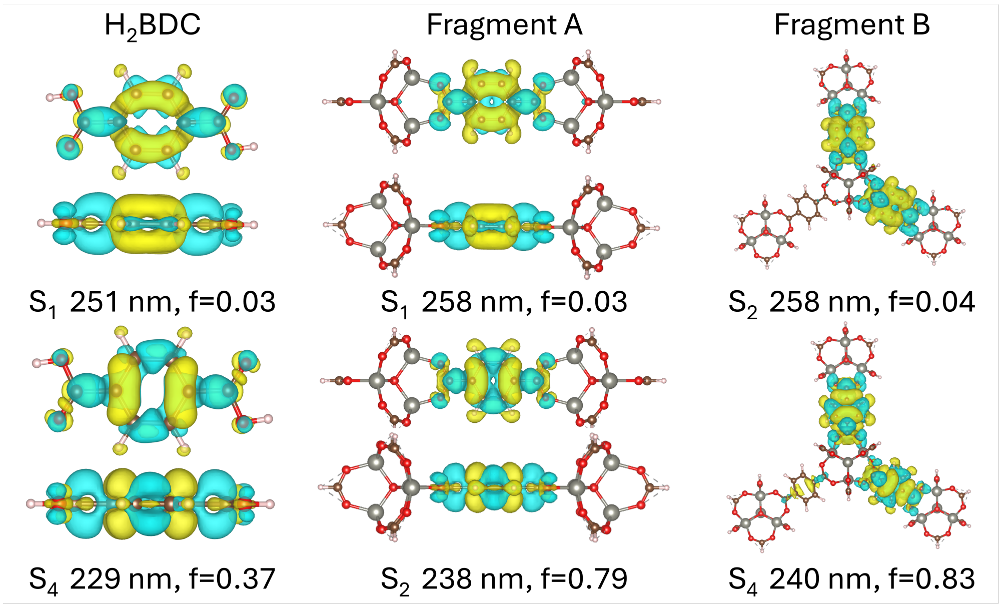

Figure S7: Visualisation of S_n-S_0 density difference plots of the first bright state (f>0.01) and first intense band (f>0.3) in H_2BDC, Fragment A and Fragment B using TD-CAM-B3LYP/cc-pvDZ. The view of the π nodal plane is shown.

Table S14: First bright state (f>0.01) and first intense absorption band (f>0.3) of each MOF-5 model in vacuum using TDDFT/cc-pVDZ cluster model.

Model	Functional	First Bright State (f>0.01)			First Intense Band (f>0.5)		
		S_n	Energy /eV	f	S_n	Energy /eV	f
H_2BDC	PBE0	S_3	4.76	0.029	S_4	5.27	0.39
	B3LYP	S_3	4.62	0.027	S_4	5.13	0.40
	CAM-B3LYP	S_1	4.93	0.031	S_4	5.42	0.38
	ωB97X-D	S_1	4.91	0.031	S_4	5.42	0.37
Fragment A	PBE0	S_1	4.62	0.023	S_2	5.05	0.84
	B3LYP	S_1	4.47	0.021	S_2	4.90	0.85
	CAM-B3LYP	S_1	4.81	0.025	S_2	5.21	0.79
	ωB97X-D	S_1	4.81	0.026	S_2	5.24	0.79
Fragment B	PBE0	S_2	4.62	0.032	S_4	5.01	0.87
	B3LYP	S_2	4.48	0.029	S_{10}	4.85	0.69
	CAM-B3LYP	S_2	4.81	0.035	S_4	5.17	0.83
	ωB97X-D	S_2	4.81	0.036	S_4	5.20	0.83

Table S15: First and second bright states (f>0.01) of Fragment A using TDDFT/cc-pVDZ embedded cluster models at the crystal (KS-DFT) geometry.

Functional	Scheme	First Bright State			Second Bright State		
		S_n	Energy /eV	f	S_n	Energy /eV	f
PBE0	Z0	S_1	4.535	0.024	S_2	4.910	0.840
	Z1	S_1	4.535	0.024	S_2	4.910	0.840
	Z2	S_1	4.528	0.024	S_2	4.906	0.842
	Z3	S_1	4.565	0.023	S_2	4.926	0.834
	RC	S_1	4.535	0.024	S_2	4.910	0.840
	RCD	S_1	4.535	0.024	S_2	4.910	0.840
B3LYP	Z0	S_1	4.426	0.021	S_2	4.804	0.839
	Z1	S_1	4.426	0.021	S_2	4.804	0.839
	Z2	S_1	4.418	0.021	S_2	4.800	0.841
	Z3	S_1	4.456	0.021	S_2	4.821	0.833
	RC	S_1	4.426	0.021	S_2	4.804	0.839
	RCD	S_1	4.426	0.021	S_2	4.804	0.839
CAM-B3LYP	Z0	S_1	4.715	0.027	S_2	5.040	0.802
	Z1	S_1	4.715	0.027	S_2	5.040	0.802
	Z2	S_1	4.709	0.027	S_2	5.036	0.804
	Z3	S_1	4.741	0.026	S_2	5.056	0.796
	RC	S_1	4.715	0.027	S_2	5.040	0.802
	RCD	S_1	4.715	0.027	S_2	5.040	0.802
ωB97X-D	Z0	S_1	4.717	0.027	S_2	5.065	0.801
	Z1	S_1	4.717	0.027	S_2	5.065	0.801
	Z2	S_1	4.711	0.028	S_2	5.061	0.803
	Z3	S_1	4.743	0.026	S_2	5.081	0.795
	RC	S_1	4.717	0.027	S_2	5.065	0.801
	RCD	S_1	4.717	0.027	S_2	5.065	0.801

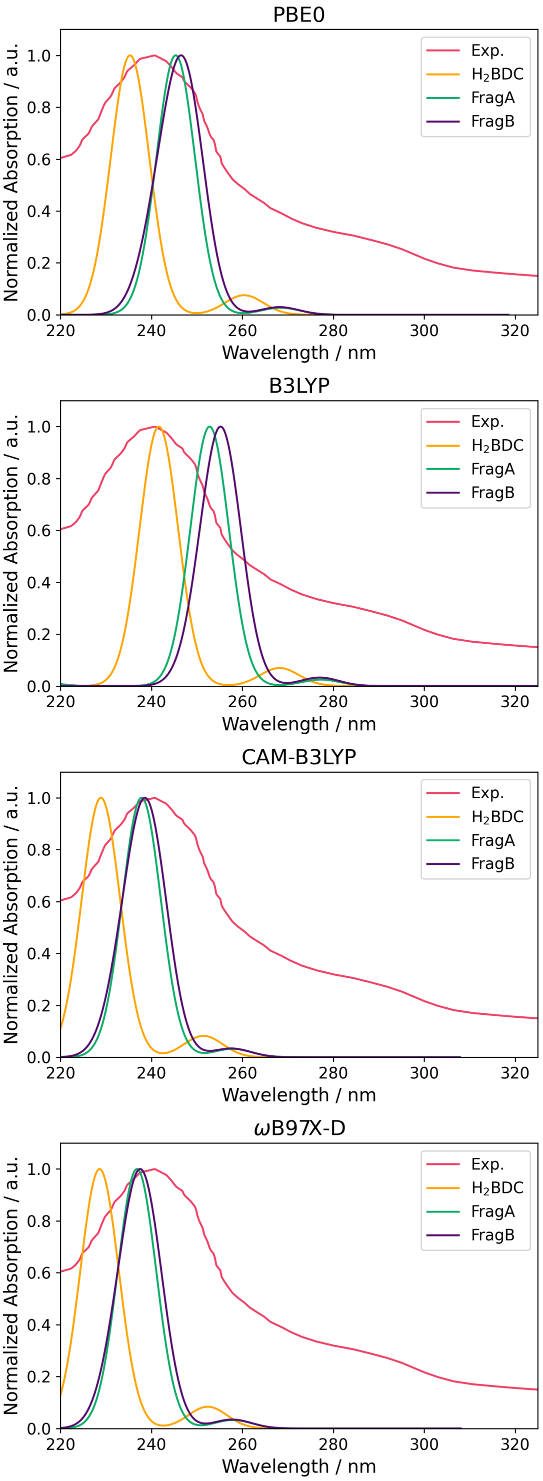

Figure S8: TDDFT calculations on clusters of BDC, Fragment A and Fragment B gas-phase for B3LYP, PBE0, CAM-B3LYP, and ωB97X-D in the cc-PVDZ basis set at the S_0-optimised geometry. Line spectra were broadened using Gaussian broadening (FWHM = 30 nm). Experimental spectrum plotted from Ref 21.

Table S16: First and second bright states (f>0.01) of Fragment A using TDDFT/cc-pVDZ embedded cluster models at the S_0-optimised ONIOM(DFT/cc-pVDZ:xTB)-EE geometry for the given functional.

Functional	Scheme	First Bright State			Second Bright State		
		S_n	Energy /eV	f	S_n	Energy /eV	f
PBE0	Z0	S_1	4.638	0.023	S_2	5.023	0.824
	Z1	S_1	4.637	0.023	S_2	5.022	0.824
	Z2	S_1	4.628	0.023	S_2	5.017	0.827
	Z3	S_1	4.668	0.022	S_2	5.040	0.817
	RC	S_1	4.637	0.023	S_2	5.022	0.824
	RCD	S_1	4.640	0.023	S_2	5.026	0.824
B3LYP	Z0	S_1	4.493	0.021	S_2	4.872	0.834
	Z1	S_1	4.492	0.021	S_2	4.872	0.834
	Z2	S_1	4.486	0.021	S_2	4.868	0.836
	Z3	S_1	4.523	0.020	S_2	4.889	0.826
	RC	S_1	4.492	0.021	S_2	4.872	0.834
	RCD	S_1	4.493	0.021	S_2	4.872	0.834
CAM-B3LYP	Z0	S_1	4.826	0.026	S_2	5.178	0.779
	Z1	S_1	4.826	0.026	S_2	5.178	0.779
	Z2	S_1	4.820	0.026	S_2	5.174	0.782
	Z3	S_1	4.849	0.025	S_2	5.195	0.772
	RC	S_1	4.826	0.026	S_2	5.178	0.779
	RCD	S_1	4.826	0.026	S_2	5.178	0.779
ωB97X-D	Z0	S_1	4.831	0.026	S_2	5.202	0.777
	Z1	S_1	4.829	0.026	S_2	5.199	0.777
	Z2	S_1	4.823	0.026	S_2	5.195	0.779
	Z3	S_1	4.851	0.025	S_2	5.215	0.769
	RC	S_1	4.826	0.026	S_2	5.200	0.777
	RCD	S_1	4.825	0.026	S_2	5.200	0.777

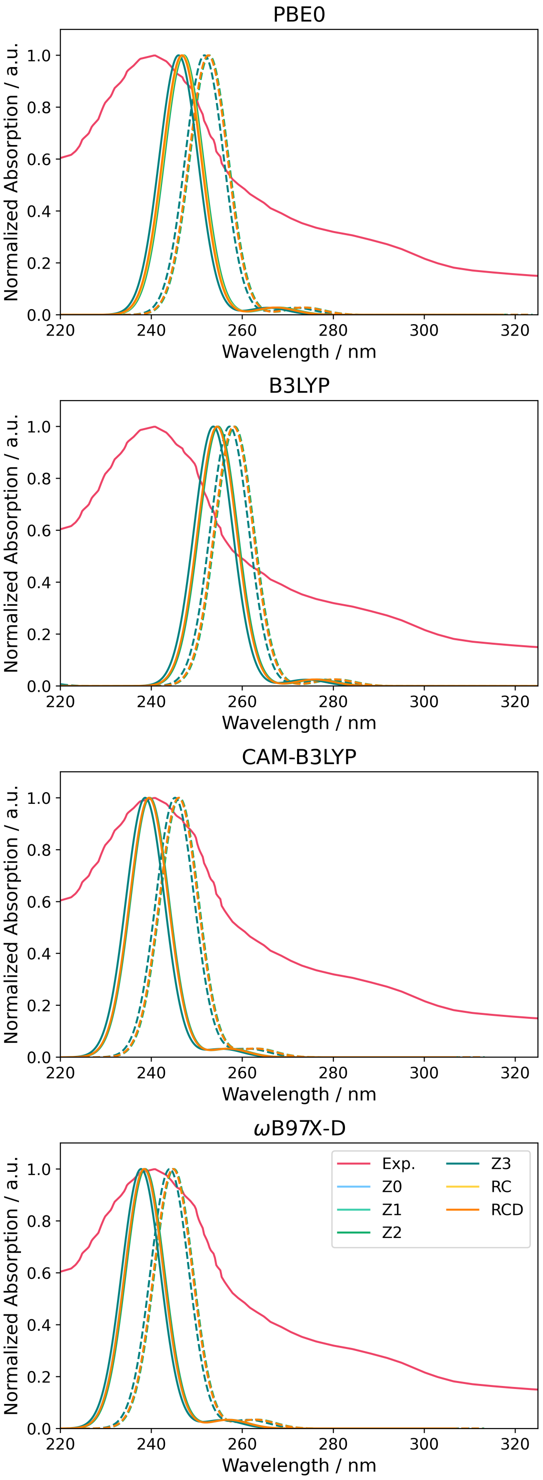

Figure S9: Calculated absorption spectrum of Fragment A using ONIOM(TDDFT/cc-pVDZ:xTB) at both relaxed periodic DFT geometry (dashed-line) and ONIOM-optimised S_0 geometry (solid-line) for the Z1, Z2, and Z3 redistribution schemes. The PBE0, B3LYP, CAM-B3LYP functionals were used, in the cc-pVDZ basis set. The experimental spectrum (Ref. 21) and PBE0/GW/BSE spectrum (Ref. 20) are also plotted.

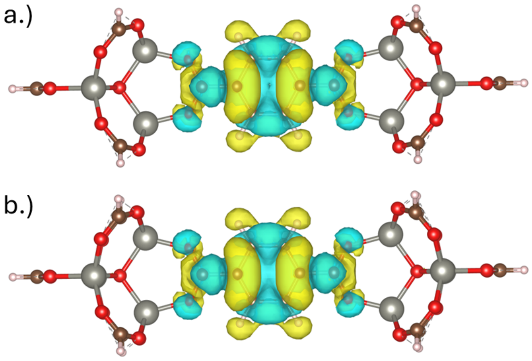

Figure S10: Visualisation of S_2-S_0 density difference for ONIOM(TD-B3LYP/cc-pVDZ:xTB)-EE at the a) periodic DFT geometry and b) the S_0-ONIOM geometry. In both cases, charges are redistributed with the Z3 scheme.

6.2 S_1 minima of embedded cluster calculations

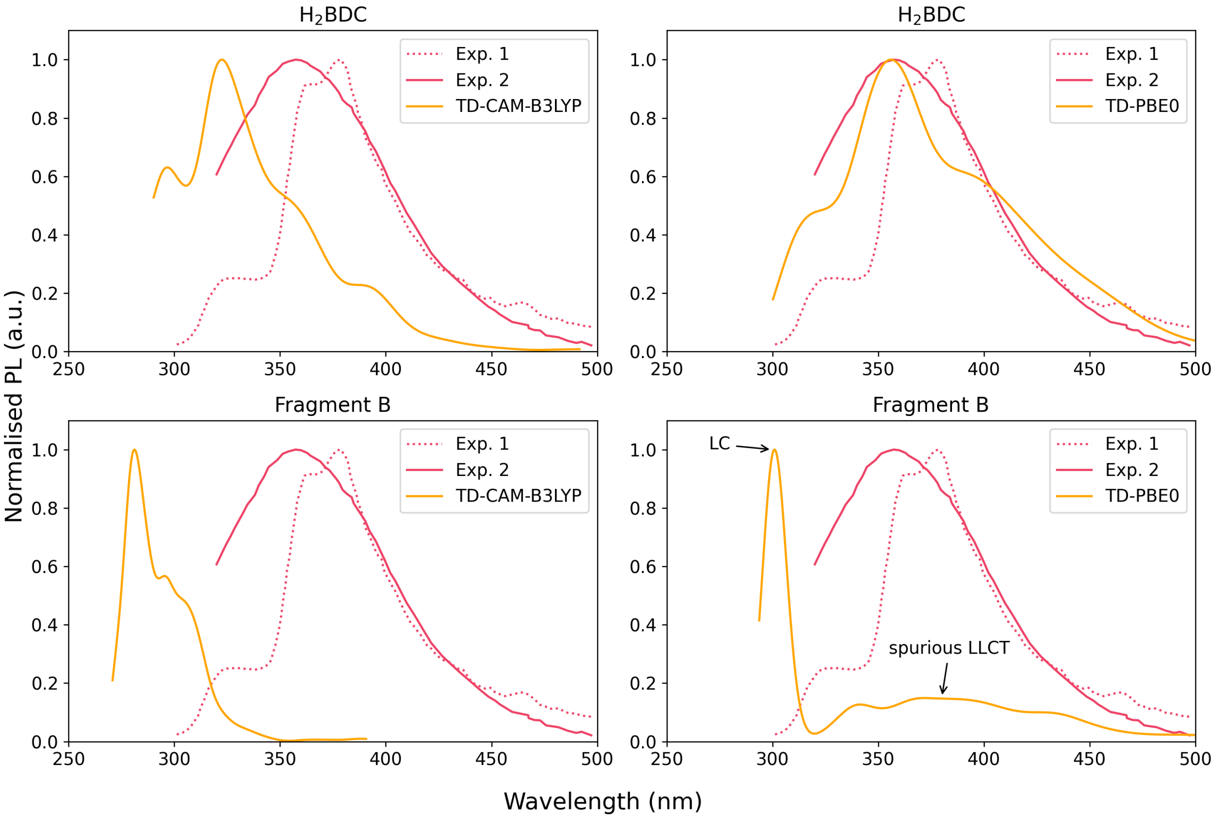

Figure S11: Emission spectra calculated from the NEA of H_2BDC (top) and Fragment B (bottom) for the CAM-B3LYP (left) and PBE0 (right) functionals. TDDFT in the cc-pVDZ basis set is used, with the LANLDZ2 basis set for Zn atoms. The experimental spectra are reproduced from Ref. 21 (Exp. 1) and Ref. 31 (Exp. 2).

6.3 Additional 'Square' MOF-5 cluster

To test the convergence with respect to model size, we ran some small exploratory minimizations on a square cluster model (Figure S12)

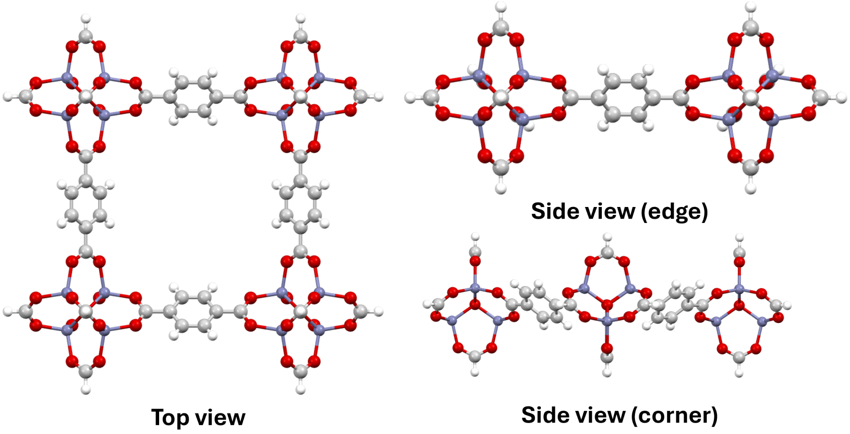

Figure S12: Square cluster model for MOF-5. The model is truncated from the periodic crystal, and saturated with hydrogen link atoms.

Functional	S_1-min (eV)	Osc. Strength
B3LYP	4.08	0.023
PBE0	4.22	0.025
ωB97X-D	4.41	0.030
CAM-B3LYP	4.42	0.029

Table S17: S_1 minimima in vacuum and oscillator strengths (TDDFT/cc-pVDZ).

References

[1] C. Plett, A. Katbashev, S. Ehlert, S. Grimme and M. Bursch, *Physical Chemistry Chemical Physics*, 2023, **25**, 17860–17868.

[2] S. Dapprich, I. Komáromi, K. S. Byun, K. Morokuma and M. J. Frisch, *J. Mol. Structure: Theochem*, 1999, 1–21.

[3] L. W. Chung, W. M. C. Sameera, R. Ramozzi, A. J. Page, M. Hatanaka, G. P. Petrova, T. V. Harris, X. Li, Z. Ke, F. Liu, H.-B. Li, L. Ding and K. Morokuma, *Chemical Reviews*, 2015, **115**, 5678–5796.

[4] M. Dommett, M. Rivera, M. T. H. Smith and R. Crespo-Otero, *Journal of Materials Chemistry C*, 2020, **8**, 2558–2568.

[5] M. Rivera, M. Dommett and R. Crespo-Otero, *Journal of Chemical Theory and Computation*, 2019, **15**, 2504–2516.

[6] M. Rivera, M. Dommett, A. Sidat, W. Rahim and R. Crespo-Otero, *Journal of Computational Chemistry*, 2020, **41**, 1045–1058.

[7] A. Sidat, M. Ingham, M. Rivera, A. J. Misquitta and R. Crespo-Otero, *The Journal of Chemical Physics*, 2023, **159**, 244108.

[8] A. Sidat, F. J. Hernández, L. Stojanović, A. J. Misquitta and R. Crespo-Otero, *Physical Chemistry Chemical Physics*, 2022, **24**, 29437–29450.

[9] H. Minemawari, M. Tanaka, S. Tsuzuki, S. Inoue, T. Yamada, R. Kumai, Y. Shimoi and T. Hasegawa, *Chemistry of Materials*, 2017, **29**, 1245–1254.

[10] G. Kresse and J. Furthmüller, *Physical Review B*, 1996, **54**, 11169–11186.

[11] T. D. Kühne, M. Iannuzzi, M. Del Ben, V. V. Rybkin, P. Seewald, F. Stein, T. Laino, R. Z. Khaliullin, O. Schütt, F. Schiffmann, D. Golze, J. Wilhelm, S. Chulkov, M. H. Bani-Hashemian, V. Weber, U. Borštnik, M. Taillefumier, A. S. Jakobovits, A. Lazzaro, H. Pabst, T. Müller, R. Schade, M. Guidon, S. Andermatt, N. Holmberg, G. K. Schenter, A. Hehn, A. Bussy, F. Belleflamme, G. Tabacchi, A. Glöß, M. Lass, I. Bethune, C. J. Mundy, C. Plessl, M. Watkins, J. VandeVondele, M. Krack and J. Hutter, *The Journal of Chemical Physics*, 2020, **152**, 194103.

[12] T. Lu and F. Chen, *Journal of Computational Chemistry*, 2012, **33**, 580–592.

[13] D. Fazzi, M. Barbatti and W. Thiel, *Physical Chemistry Chemical Physics*, 2015, **17**, 7787–7799.

[14] J. L. Mancuso, A. M. Mroz, K. N. Le and C. H. Hendon, *Chemical Reviews*, 2020, **120**, 8641–8715.

[15] A. Ortega-Guerrero, M. Fumanal, G. Capano, I. Tavernelli and B. Smit, *Chemistry of Materials*, 2020, **32**, 4194–4204.

[16] M. Fumanal, C. Corminboeuf, B. Smit and I. Tavernelli, *Physical Chemistry Chemical Physics*, 2020, **22**, 19512–19521.

[17] M. Ingham, A. Aziz, D. D. Tommaso and R. Crespo-Otero, *Materials Advances*, 2023, **4**, 5388–5419.

[18] M. E. Casida, in *Recent Advances in Density Functional Methods*, WORLD SCIENTIFIC, 1995, vol. Volume 1 of Recent Advances in Computational Chemistry, pp. 155–192.

[19] J. Strand, S. K. Chulkov, M. B. Watkins and A. L. Shluger, *The Journal of Chemical Physics*, 2019, **150**, 044702.

[20] A. R. Kshirsagar, X. Blase, C. Attaccalite and R. Poloni, *The Journal of Physical Chemistry Letters*, 2021, **12**, 4045–4051.

[21] H. Rathnayake, S. Saha, S. Dawood, S. Loeffler and J. Starobin, *The Journal of Physical Chemistry Letters*, 2021, **12**, 884–891.

[22] M. Barbatti, M. Ruckenbauer, F. Plasser, J. Pittner, G. Granucci, M. Persico and H. Lischka, *WIREs Computational Molecular Science*, 2014, **4**, 26–33.

[23] R. Crespo-Otero and M. Barbatti, *Theoretical Chemistry Accounts*, 2012, **131**, 1237.

[24] M. Barbatti and K. Sen, *International Journal of Quantum Chemistry*, 2016, **116**, 762–771.

[25] W. Park, K. Komarov, S. Lee and C. H. Choi, *The Journal of Physical Chemistry Letters*, 2023, **14**, 8896–8908.

[26] V. Mironov, K. Komarov, J. Li, I. Gerasimov, H. Nakata, M. Mazaherifar, K. Ishimura, W. Park, A. Lashkaripour, M. Oh, M. Huix-Rotllant, S. Lee and C. H. Choi, *Journal of Chemical Theory and Computation*, 2024.

[27] M. Kobayashi, J. Chen, T. C. Chung, F. Moraes, A. J. Heeger and F. Wudl, *Synthetic Metals*, 1984, **9**, 77–86.

[28] H.-W. Tsai, K.-L. Hsueh, M.-H. Chen and C.-W. Hong, *Crystals*, 2021, **11**, 1292.

[29] X.-P. Wu, L. Gagliardi and D. G. Truhlar, *Journal of Chemical Theory and Computation*, 2019, **15**, 4208–4217.

[30] T. Tsuneda and K. Hirao, *WIREs Computational Molecular Science*, 2014, **4**, 375–390.

[31] V. Villemot, M. Hamel, R. B. Pansu, I. Leray and G. H. V. Bertrand, *RSC Advances*, 2020, **10**, 18418–18422.
